# Sustainable Photocatalytic Synthesis of Glitazones via Riboflavin Tetraacetate

**DOI:** 10.1021/acs.joc.5c01306

**Published:** 2025-07-05

**Authors:** Sarah Jane Rezzi, Marco Koten, Rita Maria Concetta Di Martino, Gianluca Papeo, Tracey Pirali, Marina Caldarelli

**Affiliations:** † Department of Pharmaceutical Sciences, Università del Piemonte Orientale, Largo Donegani 2, 28100 Novara, Italy; ‡ Nerviano Medical Sciences Srl, viale Pasteur 10, 20014 Nerviano (Milano), Italy

## Abstract

A more sustainable and versatile method to access antidiabetic glitazones is demanded. Herein, we report the unprecedented photocatalytic coupling of *N*-protected methylene thiazolidinediones with radical precursors (organoborates or carboxylic acids) under riboflavin tetraacetate catalysis. The methodology accepts various functional groups and affords (Het)­Ar-(CH_2_)_n_-thiazolidinediones by transition-metal-free organic photoredox catalysis under mild conditions. The applicability of the developed protocol is showcased by the three-step preparation of the antidiabetic pioglitazone drug.

## Graphical abstract

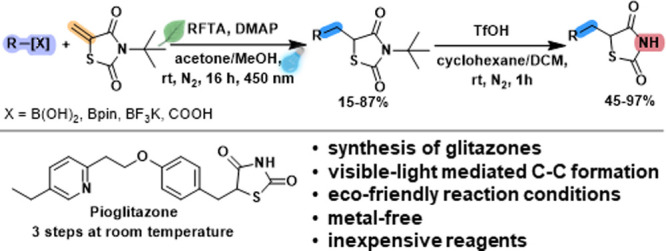

## Introduction

The peroxisome proliferator-activated receptors (PPARs) are a family of nuclear receptors that play critical roles in various metabolic processes, including glucose and lipid metabolism, inflammation, and cell differentiation.^1^ Among the three PPAR subtypes, PPARγ has gained significant attention due to its involvement in the pathophysiology of type 2 diabetes mellitus.^2,3^ Thiazolidinedione (TZD)-containing drugs, such as azemiglitazone, pioglitazone and rosiglitazone (Figure 1a), act as agonists of PPARγ, enhancing insulin sensitivity primarily in adipose tissue and thereby improving glycaemic control in diabetic patients.^4–6^ Of note, recent studies on deuterium-stabilized forms of pioglitazone have shown differences between the two enantiomers, leading to the development of *d*_1_-(*R*)-pioglitazone (PXL065, Figure 1a) for nonalcoholic steatohepatitis and X-linked adrenoleukodystrophy.^7^

**Figure 1:**
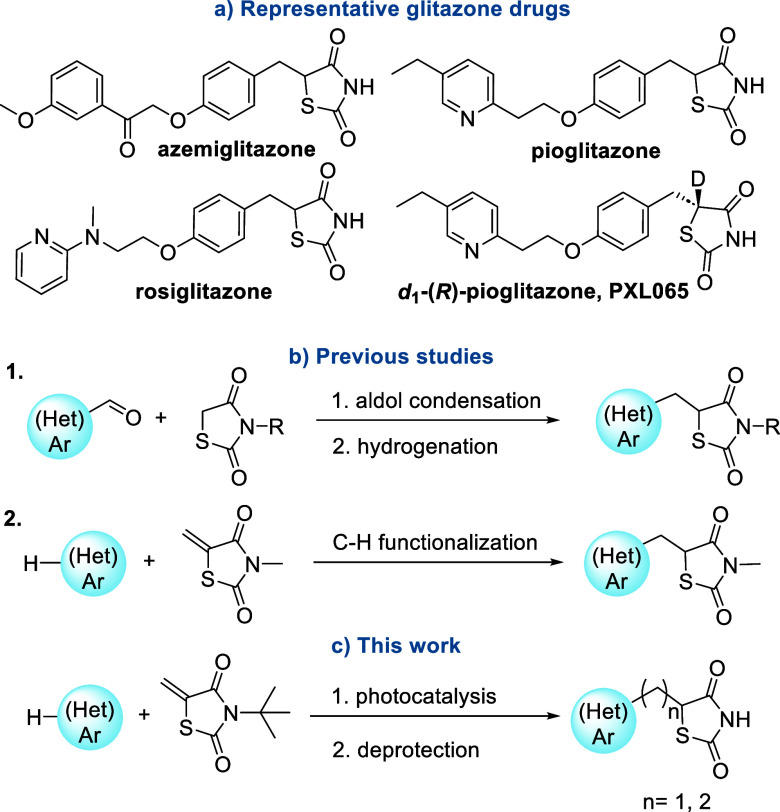
Representative glitazone drugs and synthetic approaches to (Het)­Ar–CH_2_–TZD.

The synthesis of (Het)­Ar–CH_2_–TZDs involves various chemical methodologies that have evolved over time and have been extensively reviewed.^8,9^ The most common method typically begins with the aldol condensation of appropriate aldehydes with TZD followed by olefin hydrogenation (Figure 1b1). One of the primary challenges is the reliance on prefunctionalized aldehydes, which can be costly, time-consuming to prepare and often display limited stability, resulting in undesired side reactions. Moreover, many functional groups are unstable under hydrogenation conditions, restricting the structural diversity of the final products and limiting the scope of the approach. Among the recently reported methods, Byun et al. described methylene TDZs as effective alkylating agents in catalytic C–H functionalization.^10^ Their study demonstrated *N-* and *O-*directed C-alkylation of various (hetero)­arenes using methylene TDZs under rhodium­(III) catalysis (Figure 1b2).

However, this work, while innovative, presents limitations that may hinder its broader application: (i) high cost of the rhodium-based catalyst, which also raises concerns about sustainability and potential metal contamination in the final active pharmaceutical ingredient; (ii) high temperatures; (iii) inability to access *N*-unsubstituted TZDs. Therefore, a different methodology for synthesizing the (Het)­Ar-(CH_2_)_*n*_-TZD framework capable of also generating N–H derivatives is needed. Indeed, the NH group is mandatory for the biological activity as it is involved as hydrogen bond donor in interactions with the receptor, as shown in crystal structures of PPARγ in complex with pioglitazone.^11^

## Results and Discussion

In this study, we investigated a Giese-type photoredox coupling reaction of methylene TZD with both organoborates and carboxylic acids as radical precursors, affording (Het)­Ar-(CH_2_)_*n*_-TZD (Figure 1c). Given that the photocatalytic reaction is not compatible with an *N*-unsubstituted 5-methylene TDZ, we first protected the nitrogen atom of the thiazolidinedione by using Boc anhydride in the presence of triethylamine (TEA) in dichloromethane (DCM). Contrary to our expectations and previously reported results,^12^ the reaction unexpectedly yielded 3-(*tert*-butyl)­thiazolidine-2,4-dione (**1**) and its regioisomer 2-(*tert*-butoxy)­thiazol-4­(5*H*)-one (**2**) rather than the *tert*-butyl 2,4-dioxothiazolidine-3-carboxylate (Scheme 1), which is however in agreement with Kotha et al.^13^ A plausible mechanistic hypothesis involves a concerted cyclic process (Scheme 2).^14^ Subsequent condensation of compound **1** with formaldehyde gave the required 3-(*tert*-butyl)-5- methylenethiazolidine-2,4-dione (**3**). Following the procedure described in a previously published work by our group, where α-haloacrylates behave as the olefin,^15^ we reacted **3** with biaryl boronic acid **4**, obtaining compound **5** in 43% yield. The reaction conditions involved the use of 10-(3,5-dimethoxyphenyl)-9-mesityl-1,3,6,8-tetramethoxyacridin-10-ium tetrafluoroborate (Mes-Acr) as the photocatalyst, DMAP as a Lewis base, and a solvent mixture of acetone/MeOH (1:1). The reaction was irradiated (λ = 450 nm) at room temperature for 16 h.

**Scheme 1:**
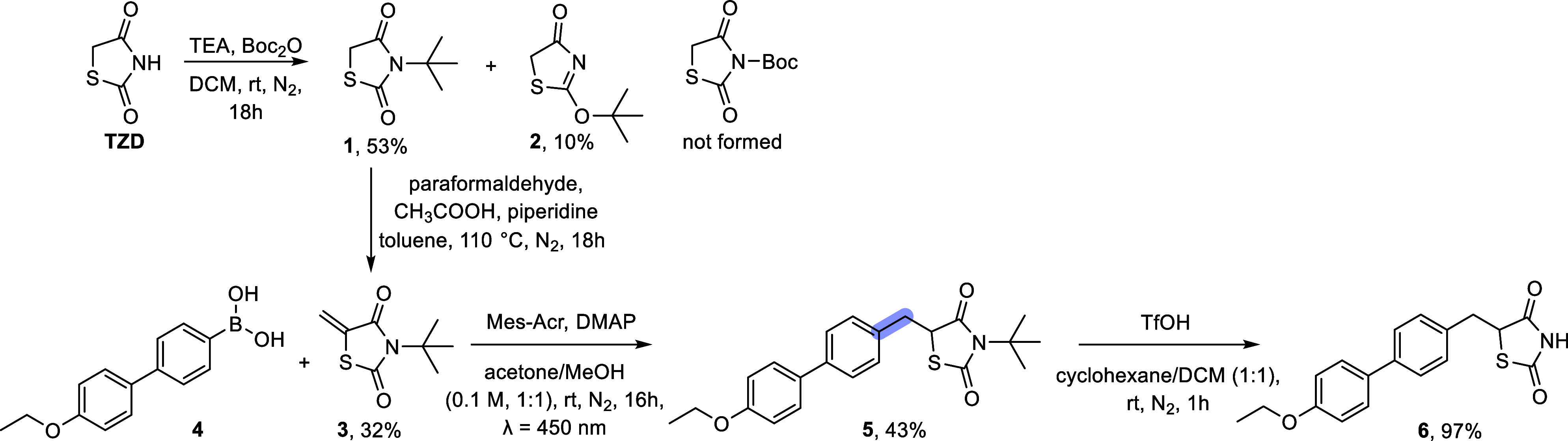
Proof-of-Concept Synthesis of Glitazone Derivative **6**

**Scheme 2:**
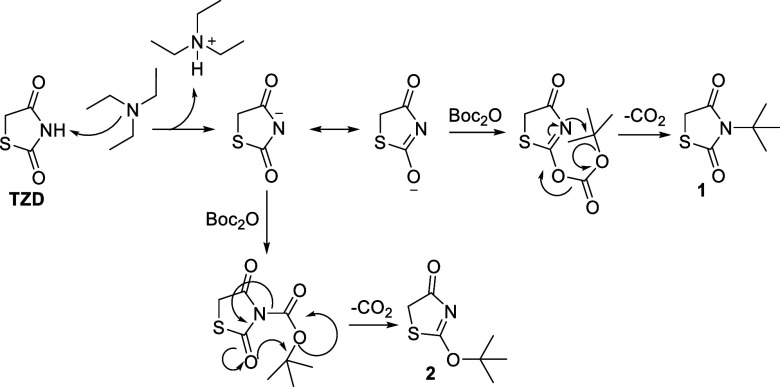
Possible Mechanism for the Formation of **1** and **2**

To explore the applicability of the *N-tert*-butyl moiety as a novel protecting group, an initial investigation was conducted on prototype compound **5** using trifluoroacetic acid (TFA), hydrochloric acid (HCl), and trifluoromethanesulfonic acid (TfOH) in various solvents. Based on these investigations, TfOH in cyclohexane/DCM (1:1) was found to be the most effective reagent for the *tert*-butyl group removal at room temperature. Of note, the free TDZ **6** was isolated with excellent yield after 1 h at room temperature, confirming the efficiency of the strategy (Scheme 1).

Having demonstrated the feasibility of our synthetic strategy, we moved on to the optimization of the photocatalytic procedure. Our investigation began using TZD **3** as the olefin acceptor and **4** as the radical precursor. A preliminary selection of the photocatalyst was performed by irradiating the reaction for 16 h at 450 nm. Among the conditions tested (Table 1, entry 1–10), we obtained the desired product **5** using acridinium-based photosensitizer Mes-Acr^16^ (entry 1), two Ir-based photocatalysts [Ir­{dFCF_3_ppy}_2_(dtbpy)]­PF_6_ and [Ir­{dFCF_3_ppy}_2_(bpy)]­PF_6_,^17^ (entries 2 and 3), 4CzIPN^18^ (entry 4), riboflavin and riboflavin tetraacetate (RFTA)^19^ (entries 5 and 6, respectively). The reaction failed without a photocatalyst (entry 7) or when using Ru­(bpy)_3_(PF_6_)_2_, Rose Bengal, and fluorescein (entries 8, 9, and 10, respectively). Among the catalysts which delivered **5**, riboflavin tetraacetate (RFTA) was selected for further investigation, as it provided the highest isolated yield and the advantage of being an inexpensive natural product-derived catalyst (entry 6, 65%).

**Table 1:**
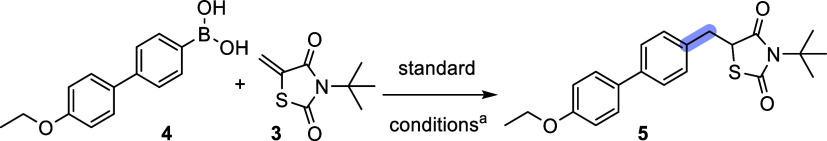
Reaction Optimization^a^

entry	PC	base	solvent	time (h)	light source (λ)	isolated yield (%)
**1**	Mes-Acr	DMAP	acetone/MeOH	16	450 nm	48
**2**	[Ir{dFCF_3_ppy}_2_(dtbpy)]PF_6_	DMAP	acetone/MeOH	16	450 nm	64
**3**	[Ir{dFCF_3_ppy}_2_(bpy)]PF_6_	DMAP	acetone/MeOH	16	450 nm	52
**4**	4CzIPN	DMAP	acetone/MeOH	16	450 nm	39
**5**	Riboflavin	DMAP	acetone/MeOH	16	450 nm	39
**6**	RFTA	DMAP	acetone/MeOH	16	450 nm	65
**7**	none	DMAP	acetone/MeOH	16	450 nm	nr
**8**	Ru(bpy)_3_(PF_6_)_2_	DMAP	acetone/MeOH	16	450 nm	nr
**9**	Rose Bengal	DMAP	acetone/MeOH	16	450 nm	nr
**10**	Fluorescein	DMAP	acetone/MeOH	16	450 nm	nr
**11**	RFTA		acetone/MeOH	16	450 nm	51
**12**	RFTA (5 mol %)		acetone/MeOH	16	450 nm	55
**13**	RFTA	DIPEA	acetone/MeOH	16	450 nm	36
**14**	RFTA	DBU	acetone/MeOH	16	450 nm	nr
**15**	RFTA	PPh_3_	acetone/MeOH	16	450 nm	60
**16**	RFTA	DMAP	MeOH	16	450 nm	59
**17**	RFTA	DMAP	EtOH	16	450 nm	20
**18**	RFTA	DMAP	MeCN	16	450 nm	nr
**19**	RFTA	DMAP	acetone/MeOH	3	450 nm	traces
**20**	RFTA	DMAP	acetone/MeOH	16		nr
**21**	RFTA	DMAP	acetone/MeOH	6	sunlight	25

a (a) Standard conditions: **4** (0.1 mmol, 1 equiv), RFTA (2.5 mol %), DMAP (0.25 equiv), and *tert*-butyl 5-methylene-2,4-dioxothiazolidine-3-carboxylate **3** (3 equiv) in acetone/MeOH (0.1 M, concentration referred to boronic acid, 1:1) were stirred under a nitrogen atmosphere and irradiated with SynLED Parallel Photoreactor (λ = 450 nm) for 16 h; nr = no reaction.

Further optimization of the reaction conditions (Table 1, entries 11–15) confirmed that DMAP (Table 1, entry 6, 65%) was the best Lewis base among those tested, and a degassed acetone/MeOH (1:1) mixture (Table 1, entry 6) was the most performing solvent (compared to Table 1, entries 16–18) for achieving the desired glitazone product. Reducing the reaction time or conducting the reaction in the dark led to minimal or no product formation (Table 1, entry 19 and 20, respectively).

Interestingly, in the absence of DMAP (Table 1, entry 11), the yield was still 51%, suggesting that RFTA may act both as the photocatalyst and as a Lewis base, as previously reported by González-Gómez.^20^ Encouraged by this result, we repeated the reaction without DMAP and with an increased amount of RFTA (5 mol %, entry 12). This led to a slightly improved yield of 55%, which, however, remained lower than the yield obtained in the presence of both DMAP and RFTA at 2.5 mol % (entry 6).

Notably, when the reaction mixture was exposed to direct sunlight,^21^ the starting material was completely consumed within 6 h (Table 1, entry 21). However, the product yield was significantly lower, and several uncharacterized impurities were observed.

With the optimal reaction conditions in place, the scope and limitations of the reaction were explored by testing a variety of radical precursors, including aromatic boronic acids, phenyl and benzyl pinacol boronates, trifluoroborates, and several alkyl carboxylic acids. A wide array of products obtained through the photocatalytic process described above are showcased in Figure 2, highlighting the versatility of the methodology in accommodating different substrates and functional groups. Among the tested substrates, aromatic boronic acids were the most represented and afforded compounds **5** and **7**–**28** featuring different functional groups such as ethers, thioethers, amides, carbamates, and heterocycles. A variety of products were efficiently synthesized from ether-substituted boronic acids. For example, methoxy, ethoxy and benzyloxy boronic acids gave the desired products (e.g., **5**, **7**–**9**, **15**–**17**) in moderate to high yields. The yields further increased in the presence of additional electron-donating groups, as observed for products such as **10**–**12** and **14**, while *ortho*-methoxy boronic acids did not react, likely due to steric hindrance. Notably, the introduction of a fluorine atom at the *meta* position (**18**) did not influence the reactivity compared to the corresponding nonfluorinated analogue **7**. Similarly, thioether-substituted boronic acids were well-tolerated. Substrates like *para*-methylthio (**19**) and *meta*-methylthio (**20**) boronic acids yielded products efficiently, underscoring the robustness of our methodology on sulfur-containing functionalities. Similarly, amide-containing boronic acids, such as *para*-acetamido (**21**) or *meta*-acetamido (**22**) derivatives, delivered the desired compounds in good yields. Carbamates, represented by substrates like *N*-Boc-protected phenylboronic acids (**23**), also performed well.^22^ Notably, heterocyclic boronic acids, such as those containing benzothiophene (**24**–**26**), indazole (**27**) and Boc-protected indole (**28**) moieties, provided the corresponding glitazone. These compounds are highly relevant to drug discovery, as heterocycles are privileged scaffolds in medicinal chemistry^23–25^

**Figure 2:**
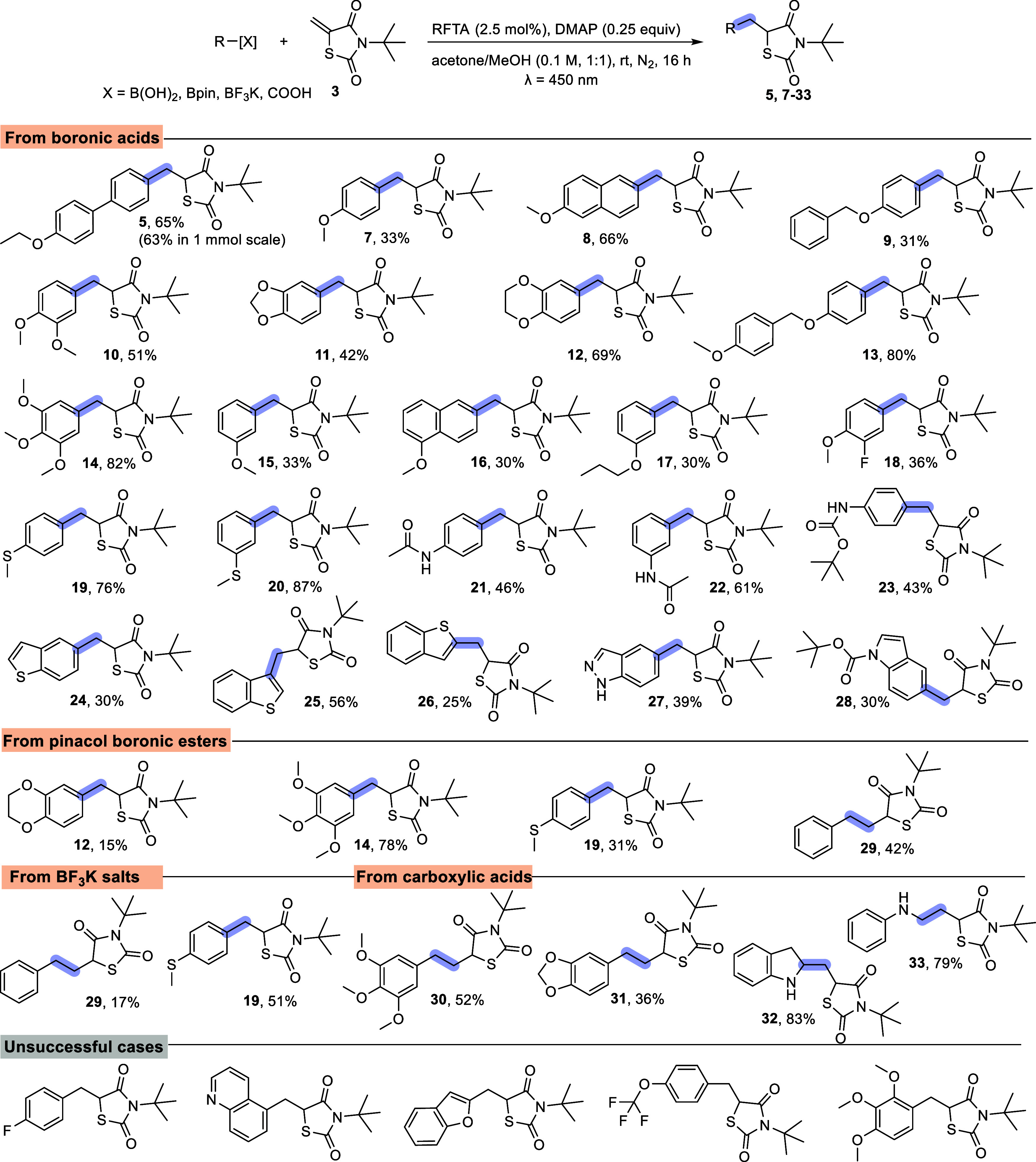
Scope of the reaction.

Pinacol boronic esters, including phenyl (**12**, **14**, **19**) and benzyl (**29**) derivatives, offered an alternative to the corresponding boronic acids and proved to be moderate to excellent reaction partners, suggesting that this class of substrates merits further exploration. Similarly, trifluoroborate salts, such as potassium phenyltrifluoroborate and potassium methylthiophenyltrifluoroborate delivered the corresponding **29** and **19**, respectively. The photoredox reaction was further extended to benzyl and alkyl carboxylic acids, which served as inexpensive and readily available radical precursors, affording the desired products **30**–**33**. While the results were overall promising, certain reactions did not yield the desired products. As anticipated, substrates containing electron-withdrawing groups or sterically hindered boronic acids showed limited reactivity.

To assess the reliability of this protocol, a scaled-up reaction was conducted with 1 mmol of boronic acid **4** under the standard batch conditions, resulting in a 63% yield of **5.**

Having found proper conditions to remove the *tert*-butyl protecting group (vide supra), we applied them to some representative derivatives (compounds **6**, **34**–**38**, Figure 3).

**Figure 3:**
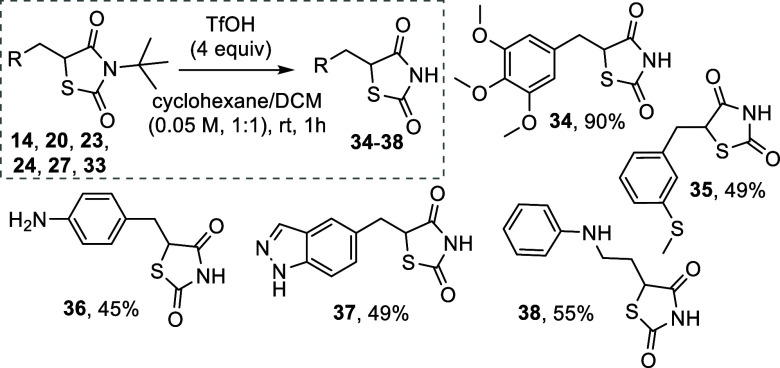
Deprotection of *N*-*tert*-butyl glitazones.

Finally, we employed our photocatalytic approach to synthesize pioglitazone **44** (Scheme 3), whose industrial preparation typically includes the condensation of the properly functionalized aldehyde with 2,4-thiazolidindione at reflux followed by hydrogenation under heating. Conversely, our process begins with a Mitsunobu reaction between compound **39** and pinacol boronic ester **40**, forming pinacol ester **41**, which is then hydrolyzed into the corresponding boronic acid **42**. A subsequent photocatalytic reaction with **3** leads to compound **43** (yield 56%), in which the *tert-*butyl group is then cleaved to give pioglitazone **44**. The entire process works at room temperature, with an overall yield of 18%. Alternatively, the pinacol ester **41** can be directly coupled with the olefin **3** to achieve **43**, albeit in a lower yield (yield 30%).

**Scheme 3:**
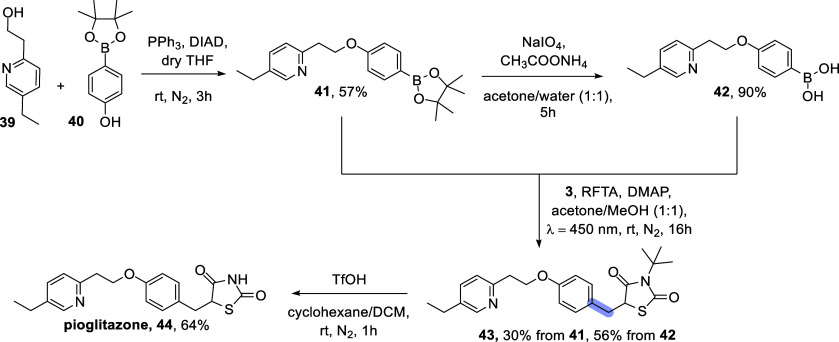
Synthesis of Pioglitazone

Notably, when the photocatalysis reaction was performed in *d*_4_-MeOH/acetone (1:1), deuterium incorporation (90%) was observed, in agreement with the proposed reaction mechanism (Figure 4),^26^ offering a practical approach to obtain deuterium-labeled derivatives with high enrichment and selectivity.

**Figure 4:**
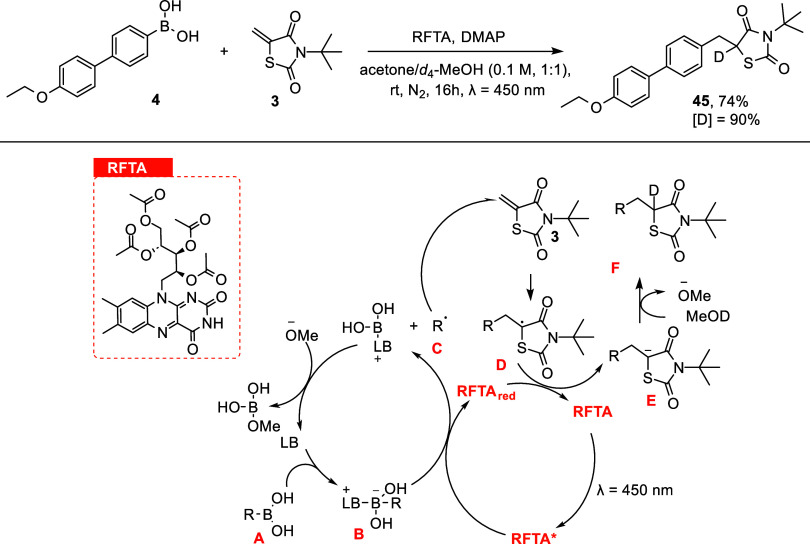
Plausible reaction mechanism and deuterium incorporation.

## Conclusion

In conclusion, by exploring a relatively under-utilized reactivity, a Giese-type protocol has been developed in which **3** is coupled with organoborates or carboxylic acids under photocatalytic conditions to access 5-substituted *N*-*tert-*Bu-TZDs. Operating under mild and environmentally benign conditions, the protocol utilizes a metal-free, natural product-derived photocatalyst and achieves excellent atom economy, with yields ranging from moderate to good. The reaction demonstrates tolerance to various functional groups, such as ethers, thioethers, amides, carbamates, and can be applied in the presence of different heterocycles. Cleavage of the *N*-*tert-*butyl group under mild conditions affords valuable *N*-unsubstituted glitazones, which have significant applications in medicinal chemistry, as exemplified by the preparation of pioglitazone. The findings of this study are expected to be of high interest in drug discovery as it can easily provide new and diverse derivatives of the glitazone class.

## Experimental Section

### General Information

Commercially available reagents and solvents were purchased from Merck, Fluorochem, Carlo Erba, BLD Pharm and Alfa Aesar and were used without further purification. When needed, reactions were performed in oven-dried or heat gun dried glassware under a positive pressure of dry nitrogen. Light irradiation was performed using the SynLED Parallel Photoreactor 2.0 (Merck) equipped with bottom-lit 450 nm LEDs (peak emission at 450 nm). Illumination was provided across a 4 × 4 reaction block array, ensuring consistent light intensity (320 lm) and a fixed irradiation angle of 45°. Reactions were conducted in borosilicate glass vials, with no optical filters employed (Figure S1). Thin layer chromatography (TLC) was carried out on 5 × 20 cm aluminum plates with a layer thickness of 0.25 cm, coated with silica gel (Merck Kieselgel 60, 230–400 mesh-ASTM). When necessary, products were developed with a KMnO_4_ staining solution (prepared by mixing a 3 g KMnO_4_ solution in 100 mL of distilled water with a solution of 4 g NaHCO_3_ in 100 mL distilled water) or *p*-anisaldehyde staining solution (prepared by mixing 135 mL of absolute ethanol, 5 mL of concentrated sulfuric acid, 1.5 mL of glacial acetic acid and 3.7 mL of *p*-anisaldehyde). Flash chromatography was performed on silica gel (Merck Kieselgel 60, 230–400 mesh-ASTM). HRMS was performed on Thermo Fisher Q-Exactive Plus equipped with an Orbitrap (ion trap) mass analyzer. Solvent used: methanol. Flow rate 0.03 mL/min. Injection volume 5 μL. ESI source was set with the following operating conditions: spray voltage, 3.50 kV; capillary temperature, 300 °C; sheath gas flow (N_2_) 23.00 L/min; sweep gas (N_2_) flow 1.00 L/min; Aux gas (N_2_) flow rate 8.00 L/min heated to 125 °C, Max Spray Current 0.60 μA. ^1^H NMR spectra were recorded at a constant temperature of 25 °C on Bruker Avance Neo 400 spectrometer operating at 400 MHz. The Chemical shifts were referenced with respect to nondeuterated residual solvent signal (DMSO-*d*_6_: 2.50 ppm for ^1^H and 39.5 ppm for ^13^C; acetone-*d*_6_: 2.05 ppm for ^1^H and 29.84 ppm for ^13^C; CDCl_3_: 7.26 ppm for ^1^H and 77.16 ppm for ^13^C). Data are reported as follows: chemical shift (δ), multiplicity (s = singlet, d = doublet, t = triplet, q = quartet, br. s = broad singlet, dd = doublet of doublets, dt = doublet of triplets, td = triplet of doublets, tt = triplet of triplets, qt = quartet of triplets, sxt = sextet, m = multiplet), coupling constants (*J*, Hz) and number of protons.

### Safety Statement

Authors did not find any unexpected, new, and/or significant hazards or risks associated with the reported work.

Synthesis procedures and/or characterization data of intermediate compounds **1**, **3**, **41**, and **42** are reported in the SI.

### General Procedure A for Photoredox Reaction for the Synthesis of Compounds **5** and **7**–**33**

An oven-dried 7 mL clear vial equipped with a magnetic stirring bar was charged with the corresponding radical precursor (25 mg), RFTA (2.5 mol %), DMAP (0.25 equiv) and *tert*-butyl 5-methylene-2,4-dioxothiazolidine-3-carboxylate **3** (3 equiv). The vial was sealed with a screw cap with septum and 3 cycles vacuum/nitrogen were performed. A 1:1 acetone/MeOH solvent mixture (purged with nitrogen for 15 min) (0.88 mL) was added. The tube was irradiated with SynLED Parallel Photoreactor (450 nm) for 16 h. The reaction was concentrated in vacuo, reconstituted in DCM, and purified through flash chromatography.

### General Procedure B for Deprotection Reaction for the Synthesis of Compounds **6** and **34**–**38**

To a solution of the corresponding *tert*-butyl derivative (1 equiv) in anhydrous and N_2_ purged cyclohexane/DCM (0.01 M, 1:1), trifluoromethanesulfonic acid (4 equiv) was added. The reaction was stirred for 1 h at room temperature under nitrogen atmosphere. Upon reaction completion, aqueous NaHCO_3_ was added dropwise, and the crude was extracted twice with EtOAc. The combined organic layers were then dried over anhydrous MgSO_4_, filtered and concentrated under *vacuum*. The crude was then purified through silica gel flash chromatography.

#### 3-(*tert*-Butyl)-5-((4′-ethoxy-[1,1′-biphenyl]-4-yl)­methyl)­thiazolidine-2,4-dione (**5**)

White solid (25.7 mg, yield: 65%). Eluent: PE/EtOAc 98:2. ^1^H NMR (400 MHz, CDCl_3_) δ 7.50 (d, *J* = 7.5 Hz, 4H), 7.27 (d, *J* = 7.4 Hz, 2H), 6.96 (d, *J* = 8.7 Hz, 2H), 4.28 (dd, *Js* = 8.9, 3.7 Hz, 1H), 4.07 (q, *J* = 7.0 Hz, 2H), 3.44 (d, *J* = 3.8 Hz, 1H), 3.13 (dd, *Js* = 13.9, 9.0 Hz, 1H), 1.54 (s, 9H), 1.44 (t, *J* = 7.0 Hz, 3H). ^13^C­{1H} NMR (101 MHz, CDCl_3_) δ 175.4, 171.7, 158.7, 140.2, 134.1, 133.0, 129.9 (2C), 128.1 (2C), 127.0 (2C), 114.9 (2C), 63.6, 62.1, 50.0, 38.8, 28.5 (3C), 15.0. HRMS (ESI) *m*/*z* calculated for C_22_H_25_NNaO_3_S [M + Na]^+^ 406.1447, found 406.1444.

#### 5-((4′-Ethoxy-[1,1′-biphenyl]-4-yl)­methyl)­thiazolidine-2,4-dione (**6**)

Off-white solid (40.5 mg, yield: 97%). Eluent: PE/EtOAc 80:20. ^1^H NMR (400 MHz, CDCl_3_) δ 8.09 (br s, 1H), 7.51 (dd, *Js* = 8.3, 5.8 Hz, 4H), 7.27 (d, *J* = 8.2 Hz, 2H), 6.96 (d, *J* = 8.8 Hz, 2H), 4.56 (dd, *Js* = 9.7, 3.9 Hz, 1H), 4.08 (q, *J* = 7.0 Hz, 2H), 3.57 (dd, *Js* = 14.1, 3.9 Hz, 1H), 3.17 (dd, *Js* = 14.1, 9.7 Hz, 1H), 1.44 (t, *J* = 7.0 Hz, 3H). ^13^C­{1H} NMR (101 MHz, CDCl_3_) δ 173.8, 170.0, 158.8, 140.4, 134.1, 132.9, 129.7 (2C), 128.2 (2C), 127.2 (2C), 115.0 (2C), 63.7, 53.5, 38.4, 15.0. HRMS (ESI) *m*/*z* estimated for C_18_H_16_NO_3_S [M-H]^−^ 326.0856, found 326.0857.

#### 3-(*tert*-Butyl)-5-(4-methoxybenzyl)­thiazolidine-2,4-dione (**7**)

White solid (16 mg, yield: 33%). Eluent: PE/EtOAc 97:3. ^1^H NMR (400 MHz, CDCl_3_) δ 7.14 (d, *J* = 8.6 Hz, 2H), 6.84 (d, *J* = 8.7 Hz, 2H), 4.21 (dd, *Js* = 8.8, 3.8 Hz, 1H), 3.79 (s, 3H), 3.35 (dd, *Js* = 14.0, 3.8 Hz, 1H), 3.05 (dd, *Js* = 14.0, 8.7 Hz, 1H), 1.53 (s, 9H). ^13^C­{1H} NMR (101 MHz, CDCl_3_) δ 175.5, 171.8, 159.1, 130.7 (2C), 127.8, 114.1 (2C), 62.1, 55.4, 50.3, 38.3, 28.5 (3C). HRMS (ESI) *m*/*z* calculated for C_15_H_20_NO_3_S [M + H]^+^ 294.1158, found 294.1152.

#### 3-(*tert*-Butyl)-5-((6-methoxynaphthalen-2-yl)­methyl)­thiazolidine-2,4-dione (**8**)

Noncrystalline white solid (28.2 mg, yield: 66%). Eluent: PE/EtOAc 92:8. ^1^H NMR (400 MHz, CDCl_3_) δ 7.69 (d, *J* = 8.6 Hz, 2H), 7.62–7.57 (m, 1H), 7.30 (dd, *Js* = 8.4, 1.8 Hz, 1H), 7.15 (dd, *Js* = 8.9, 2.6 Hz, 1H), 7.11 (d, *J* = 2.5 Hz, 1H), 4.32 (dd, *Js* = 9.0, 3.8 Hz, 1H), 3.91 (s, 3H), 3.56 (dd, *J* = 13.9, 3.8 Hz, 1H), 3.22 (dd, *Js* = 13.9, 9.0 Hz, 1H), 1.52 (s, 9H). ^13^C­{1H} NMR (101 MHz, CDCl_3_) δ 175.5, 171.7, 157.9, 133.9, 131.0, 129.3, 128.9, 128.2, 127.9, 127.3, 119.3, 105.7, 62.1, 55.4, 50.2, 39.1, 28.5 (3C). HRMS (ESI) *m*/*z* calculated for C_19_H_22_NO_3_S [M + H]^+^ 344.1315, found 344.1312.

#### 5-(4-(Benzyloxy)­benzyl)-3-(*tert*-butyl)­thiazolidine-2,4-dione (**9**)

Noncrystalline orange solid (12.4 mg, yield: 31%). Eluent: PE/EOAc 95:5. ^1^H NMR (400 MHz, CDCl_3_) δ 7.51–7.31 (m, 5H), 7.22 (t, *J* = 7.8 Hz, 1H), 6.99–6.75 (m, 3H), 5.05 (s, 2H), 4.23 (dd, *Js* = 9.3, 3.7 Hz, 1H), 3.43 (dd, *Js* = 13.9, 3.8 Hz, 1H), 3.04 (dd, *Js* = 13.9, 9.3 Hz, 1H), 1.55 (s, 9H). ^13^C­{1H} NMR (101 MHz, CDCl_3_) δ 175.4, 171.7, 159.1, 137.6, 136.9, 129.8, 128.7 (2 C), 128.1, 127.6 (2 C), 122.0, 116.2, 113.9, 70.1, 62.1, 49.9, 39.2, 28.5 (3 C). HRMS (ESI) *m*/*z* calculated for C_21_H_23_NNaO_3_S [M + Na]^+^ 392.1291, found 392.1284.

#### 3-(*tert*-Butyl)-5-(3,4-dimethoxybenzyl)­thiazolidine-2,4-dione (**10**)

White solid (22.6 mg, yield: 51%). Eluent: Cyclohexane/EtOAc 97:3. ^1^H NMR (400 MHz, CDCl_3_) δ 6.82–6.74 (m, 3H), 4.23 (dd, *Js* = 9.0, 3.7 Hz, 1H), 3.87 (s, 3H), 3.86 (s, 3H), 3.38 (dd, *Js* = 14.0, 3.7 Hz, 1H), 3.03 (dd, *Js* = 13.9, 9.1 Hz, 1H), 1.54 (s, 9H). ^13^C­{1H} NMR (101 MHz, CDCl_3_) δ 175.4, 171.8, 149.0, 148.5, 128.4, 121.7, 112.6, 111.3, 62.1, 56.0, 56.0, 50.4, 38.8, 28.5 (3C). HRMS (ESI) *m*/*z* calculated for C_16_H_21_NNaO_4_S [M + Na]^+^ 346.1083, found 346.1081.

#### 5-(Benzo­[*d*]­[1,3]­dioxol-5-ylmethyl)-3-(*tert*-butyl)­thiazolidine-2,4-dione (**11**)

White solid (21.1 mg, yield: 42%). Eluent: Cyclohexane/EtOAc 95:5. ^1^H NMR (400 MHz, CDCl_3_) δ 6.76–6.66 (m, 3H), 5.94 (s, 2H), 4.20 (dd, *Js* = 8.9, 3.9 Hz, 1H), 3.35–3.31 (m, 1H), 3.02 (dd, *Js* = 14.0, 8.9 Hz, 1H), 1.55 (s, 9H). ^13^C­{1H} NMR (101 MHz, CDCl_3_) δ 175.4, 171.7, 147.9, 147.1, 129.5, 122.8, 109.9, 108.5, 101.2, 62.1, 50.3, 38.9, 28.5 (3C). HRMS (ESI) *m*/*z* calculated for C_15_H_17_NNaO_4_S [M + Na]^+^ 330.0770, found 330.0770.

#### 3-(*tert*-Butyl)-5-((2,3-dihydrobenzo­[*b*]­[1,4]­dioxin-6-yl)­methyl)­thiazolidine-2,4-dione (**12**)

White solid (30.6 mg, yield: 69% from boronic acid, yield: 15% from pinacol boronic ester). Eluent: PE/EtOAc 98:2. ^1^H NMR (400 MHz, CDCl_3_) δ 6.80–6.66 (m, 3H), 4.24 (s, 4H), 4.19 (dd, *Js* = 9.0, 3.8 Hz, 1H), 3.32 (dd, *Js* = 14.0, 3.8 Hz, 1H), 2.98 (dd, *Js* = 14.0, 9.0 Hz, 1H), 1.55 (s, 9H). ^13^C­{1H} NMR (101 MHz, CDCl_3_) δ 175.5, 171.9, 143.6, 143.1, 129.1, 122.5, 118.2, 117.5, 64.5 (2C), 62.1, 50.2, 38.5, 28.5 (3C). HRMS (ESI) *m*/*z* calculated for C_16_H_19_NNaO_4_S [M + Na]^+^ 344.0927, found 344.0926.

#### 3-(*tert*-Butyl)-5-(4-((4-methoxybenzyl)­oxy)­benzyl)­thiazolidine-2,4-dione (**13**)

White solid (31 mg, yield: 80%). Eluent: PE/EtOAc 80:20. ^1^H NMR (400 MHz, CDCl_3_) δ 7.36–7.32 (m, 2H), 7.17–7.07 (m, 2H), 6.96–6.82 (m, 4H), 4.97 (s, 2H), 4.21 (dd, *J*s = 8.7, 3.8 Hz, 1H), 3.81 (s, 3H), 3.35 (dd, *Js* = 14.0, 3.8 Hz, 1H), 3.05 (dd, *Js* = 14.0, 8.8 Hz, 1H), 1.52 (s, 9H). ^13^C­{1H} NMR (101 MHz, CDCl_3_) δ 175.5, 171.8, 159.6, 158.4, 130.7 (2C), 129.3 (2C), 129.02, 127.9, 115.1 (2C), 114.1 (2C), 69.9, 62.0, 55.4, 50.3, 38.3, 28.5 (3C). HRMS (ESI) *m*/*z* calculated for C_22_H_25_NNaO_4_S [M + Na]^+^ 422.1397, found 422.1389.

#### 3-(*tert*-Butyl)-5-(3,4,5-trimethoxybenzyl)­thiazolidine-2,4-dione (**14**)

White solid (33 mg, yield: 82% from boronic acid, yield: 78% from pinacol boronic ester). Eluent: Cyclohexane/EtOAc 9:1. ^1^H NMR (400 MHz, CDCl_3_) δ 6.45 (s, 2H), 4.23 (dd, *Js* = 9.3, 3.5 Hz, 1H), 3.85 (s, 6H), 3.82 (s, 3H), 3.39 (dd, *Js* = 13.8, 3.5 Hz, 1H), 3.00 (dd, *Js* = 13.8, 9.3 Hz, 1H), 1.54 (s, 9H). ^13^C­{1H} NMR (101 MHz, CDCl_3_) δ 175.3, 171.7, 153.4 (2C), 137.4, 131.6, 106.4 (2C), 62.1, 61.0, 56.2 (2C), 50.2, 39.5, 28.5 (3C). HRMS (ESI) *m*/*z* calculated for C_17_H_23_NNaO_5_S [M + Na]^+^ 376.1189, found 376.1187.

#### 3-(*tert*-Butyl)-5-(3-methoxybenzyl)­thiazolidine-2,4-dione (**15**)

White solid (16.1 mg, yield: 33%). Eluent: PE/EtOAc 97:3. ^1^H NMR (400 MHz, CDCl_3_) δ 7.22 (dd, *J* = 8.3, 7.4 Hz, 1H), 6.87–6.73 (m, 3H), 4.24 (dd, *Js* = 9.3, 3.8 Hz, 1H), 3.79 (s, 3H), 3.43 (dd, *Js* = 13.9, 3.8 Hz, 1H), 3.04 (dd, *Js* = 13.8, 9.3 Hz, 1H), 1.55 (s, 9H). ^13^C­{1H} NMR (101 MHz, CDCl_3_) δ 175.4, 171.7, 159.9, 137.5, 129.8, 121.7, 115.2, 113.0, 62.1, 55.3, 50.0, 39.2, 28.5 (3C). HRMS (ESI) *m*/*z* calculated for C_15_H_20_NO_3_S [M + H]^+^ 294.1158, found 294.1153.

#### 3-(*tert*-Butyl)-5-((5-methoxynaphthalen-2-yl)­methyl)­thiazolidine-2,4-dione (**16**)

White solid (12.4 mg, yield 30%). Eluent: PE/EtOAc 98:2. ^1^H NMR (400 MHz, CDCl_3_) δ 7.69 (d, *J* = 8.6 Hz, 2H), 7.60 (s, 1H), 7.30 (dd, *Js* = 8.4, 1.8 Hz, 1H), 7.15 (dd, *Js* = 8.9, 2.5 Hz, 1H), 7.11 (d, *Js* = 2.5 Hz, 1H), 4.33 (dd, *Js* = 9.0, 3.8 Hz, 1H), 3.92 (s, 3H), 3.57 (dd, *Js* = 13.9, 3.8 Hz, 1H), 3.22 (dd, *Js* = 13.9, 9.0 Hz, 1H), 1.51 (s, 9H). ^13^C­{1H} NMR (101 MHz, CDCl_3_) δ 175.5, 171.7, 157.9, 133.9, 131.1, 129.3, 128.9, 128.2, 128.0, 127.3, 119.3, 105.7, 62.1, 55.4, 50.3, 39.2, 28.5 (3C). HRMS (ESI) *m*/*z* calculated for C_19_H_22_NO_3_S [M + H]^+^ 344.1315, found 344.1314.

#### 3-(*tert*-Butyl)-5-(3-propoxybenzyl)­thiazolidine-2,4-dione (**17**)

White solid (13.1 mg, yield: 30%). Eluent: PE/EtOAc 99:1. ^1^H NMR (400 MHz, CDCl_3_) δ 7.20 (t, *J* = 7.9 Hz, 1H), 6.81–6.75 (m, 3H), 4.24 (dd, *Js* = 9.3, 3.8 Hz, 1H), 3.90 (t, *J* = 6.4 Hz, 2H), 3.42 (dd, *Js* = 13.9, 3.8 Hz, 1H), 3.03 (dd, *Js* = 13.8, 9.3 Hz, 1H), 1.80 (h, *J* = 7.1 Hz, 2H), 1.55 (s, 9H), 1.03 (t, *J* = 7.4 Hz, 3H). ^13^C­{1H} NMR (101 MHz, CDCl_3_) δ 175.4, 171.8, 159.4, 137.5, 129.8, 121.6, 115.7, 113.6, 69.6, 62.1, 50.1, 39.3, 28.5 (3C), 22.7, 10.7. HRMS (ESI) *m*/*z* calculated for C_17_H_22_NO_3_S [M-H]^−^ 320.1326, found 320.1326.

#### 3-(*tert*-Butyl)-5-(3-fluoro-4-methoxybenzyl)­thiazolidine-2,4-dione (**18**)

White solid (16.7 mg, yield: 36%). Eluent: Cyclohexane/EtOAc 99:1. ^1^H NMR (400 MHz, CDCl_3_) δ 6.98–6.86 (m, 3H), 4.21 (dd, *Js* = 8.5, 3.9 Hz, 1H), 3.87 (s, 3H), 3.32 (dd, *Js* = 14.1, 3.9 Hz, 1H), 3.06 (dd, *Js* = 14.1, 8.6 Hz, 1H), 1.54 (s, 9H). ^13^C­{1H} NMR (101 MHz, CDCl_3_) δ 175.3, 171.5, 152.3 (d, *J* = 246.6 Hz), 147.2 (d, *J* = 10.6 Hz), 128.6 (d, *J* = 6.2 Hz), 125.5 (d, *J* = 3.6 Hz), 117.3 (d, *J* = 18.5 Hz), 113.5 (d, *J* = 2.2 Hz), 62.2, 56.4, 49.9, 38.1 (d, *J* = 1.4 Hz), 28.5 (3C). HRMS (ESI) *m*/*z* calculated for C_15_H_18_FNNaO_3_S [M + Na]^+^ 334.0883, found 334.0883.

#### 3-(*tert*-Butyl)-5-(4-(methylthio)­benzyl)­thiazolidine-2,4-dione (**19**)

White solid (34 mg, yield: 76% from boronic acid, yield: 51% from BF_3_K salt, yield; 31% from pinacol boronic ester). Eluent: PE/EtOAc 99:1. ^1^H NMR (400 MHz, CDCl_3_) δ 7.19 (d, *J* = 8.4 Hz, 2H), 7.14 (d, *J* = 8.3 Hz, 2H), 4.22 (dd, *Js* = 8.9, 3.8 Hz, 1H), 3.39–3.35 (m, 1H), 3.06 (dd, *Js* = 13.9, 8.9 Hz, 1H), 2.46 (s, 3H), 1.53 (s, 9H). ^13^C­{1H} NMR (101 MHz, CDCl_3_) δ 175.3, 171.6, 137.9, 132.6, 130.1 (2C), 126.9 (2C), 62.1, 50.0, 38.6, 28.5 (3C), 16.0. HRMS (ESI) *m*/*z* calculated for C_15_H_19_NO_2_S_2_ [M + Na]^+^ 332.0749, found 332.0742.

#### 3-(*tert*-Butyl)-5-(3-(methylthio)­benzyl)­thiazolidine-2,4-dione (**20**)

Off-white solid (40 mg, yield: 87%). Eluent: PE/EtOAc 95:5. ^1^H NMR (400 MHz, CDCl_3_) δ 7.24 (d, *J* = 7.7 Hz, 1H), 7.16 (dt, *Js* = 7.9, 1.7 Hz, 1H), 7.10 (t, *J* = 1.9 Hz, 1H), 6.99 (dt, *Js* = 7.57, 1.5 Hz, 1H), 4.24 (dd, *Js* = 9.0, 3.8 Hz, 1H), 3.40 (dd, *Js* = 13.9, 3.8 Hz, 1H), 3.06 (dd, *Js* = 13.9, 9.0 Hz, 1H), 2.47 (s, 3H), 1.54 (s, 9H). ^13^C­{1H} NMR (101 MHz, CDCl_3_) δ 175.3, 171.6, 139.2, 136.6, 129.2, 127.4, 126.2, 125.6, 62.2, 49.8, 39.0, 28.5 (3C), 15.8. HRMS (ESI) *m*/*z* calculated for C_15_H_19_NNaO_2_S_2_ [M + Na]^+^ 332.0749, found 332.0747.

#### *N*-(4-((3-(*tert*-Butyl)-2,4-dioxothiazolidin-5-yl)­methyl)­phenyl)­acetamide (**21**)

White solid (20.7 mg, yield: 46%). Eluent: Cyclohexane/EtOAc 80:20. ^1^H NMR (400 MHz, CDCl_3_) δ 7.45 (d, *J* = 8.4 Hz, 2H), 7.20–7.15 (m, 3H), 4.22 (dd, *Js* = 8.9, 3.8 Hz, 1H), 3.39 (dd, *Js* = 14.0, 3.9 Hz, 1H), 3.06 (dd, *Js* = 14.0, 8.9 Hz, 1H), 2.17 (s, 3H), 1.54 (s, 9H). ^13^C­{1H} NMR (101 MHz, CDCl_3_) δ 175.4, 171.7, 168.4, 137.4, 131.8, 130.2 (2C), 120.0 (2C), 62.2, 50.1, 38.5, 28.5, 24.8 (3C). HRMS (ESI) *m*/*z* calculated for C_16_H_20_N_2_NaO_3_S [M + Na]^+^ 343.1086, found 343.1083.

#### *N*-(3-((3-(*tert*-Butyl)-2,4-dioxothiazolidin-5-yl)­methyl)­phenyl)­acetamide (**22**)

White solid (17.9 mg, yield: 61%). Eluent: PE/EtOAc 70:30. ^1^H NMR (400 MHz, CDCl_3_) δ 7.42 (br s, 1H), 7.40 (s, 2H), 7.25 (t, *J* = 7.7 Hz, 1H), 6.95 (d, *J* = 7.6 Hz, 1H), 4.24 (dd, *Js* = 9.3, 3.8 Hz, 1H), 3.43 (dd, *Js* = 14.0, 3.9 Hz, 1H), 3.02 (dd, *Js* = 13.9, 9.3 Hz, 1H), 2.16 (s, 3H), 1.54 (s, 9H). ^13^C­{1H} NMR (101 MHz, CDCl_3_) δ 175.3, 171.7, 168.5, 138.4, 137.0, 129.4, 125.2, 120.7, 119.0, 62.2, 49.9, 39.1, 28.5, 24.8 (3C). HRMS (ESI) *m*/*z* calculated for C_16_H_20_N_2_NaO_3_S [M + Na]^+^ 343.1087, found 343.1082.

#### *tert*-Butyl (4-((3-(*tert*-butyl)-2,4-dioxothiazolidin-5-yl)­methyl)­phenyl)­carbamate (**23**)

Noncrystalline white solid (17 mg, yield: 43%). Eluent: PE/EtOAc 87:13. ^1^H NMR (400 MHz, CDCl_3_) δ 7.30 (d, *J* = 8.4 Hz, 2H), 7.18–7.05 (m, 2H), 6.46 (s, 1H), 4.21 (dd, *Js* = 9.0, 3.8 Hz, 1H), 3.41–3.32 (m, 1H), 3.03 (dd, *Js* = 14.0, 9.0 Hz, 1H), 1.54 (s, 9H), 1.51 (s, 9H). ^13^C­{1H} NMR (101 MHz, CDCl_3_) δ 175.4, 171.7, 152.8, 137.8, 130.4, 130.2 (2C), 118.7 (2C), 80.8, 62.1, 50.2, 38.5, 28.5 (3C), 28.5 (3C). HRMS (ESI) *m*/*z* calculated for C_19_H_26_N_2_NaO_4_S [M + Na]^+^ 401.1505, found 401.1498.

#### 5-(Benzo­[*b*]­thiophen-5-ylmethyl)-3-(*tert*-butyl)­thiazolidine-2,4-dione (**24**)

Yellow solid (12.8 mg, yield: 30%). Eluent: PE/EtOAc 99:1. ^1^H NMR (400 MHz, CDCl_3_) δ 7.82 (d, *J* = 8.3 Hz, 1H), 7.67 (d, *J* = 1.7 Hz, 1H), 7.45 (d, *J* = 5.5 Hz, 1H), 7.29 (dd, *Js* = 5.4, 0.8 Hz, 1H), 7.21 (dd, *Js* = 8.3, 1.8 Hz, 1H), 4.30 (dd, *Js* = 9.0, 3.8 Hz, 1H), 3.56 (dd, *Js* = 13.9, 3.8 Hz, 1H), 3.21 (dd, *Js* = 13.9, 9.0 Hz, 1H), 1.52 (s, 9H). ^13^C­{1H} NMR (101 MHz, CDCl_3_) δ 175.4, 171.7, 140.0, 139.0, 132.0, 127.3, 125.8, 124.5, 123.8, 122.8, 62.1, 50.4, 39.1, 28.5 (3C). HRMS (ESI) *m*/*z* calculated for C_16_H_17_NNaO_2_S_2_ [M + Na]^+^ 342.0593, found 342.0589.

#### 5-(Benzo­[*b*]­thiophen-3-ylmethyl)-3-(*tert*-butyl)­thiazolidine-2,4-dione (**25**)

Transparent oil (25 mg, yield: 56%). Eluent: PE/EtOAc 90:10. ^1^H NMR (400 MHz, CDCl_3_) δ 7.89–7.84 (m, 1H), 7.82–7.78 (m, 1H), 7.45–7.35 (m, 2H), 7.28 (s, 1H), 4.38 (dd, *Js* = 9.6, 3.5 Hz, 1H), 3.75 (ddd, *Js* = 14.7, 3.5, 1.0 Hz, 1H), 3.33 (ddd, *Js* = 14.7, 9.6, 0.7 Hz, 1H), 1.52 (s, 9H). ^13^C­{1H} NMR (101 MHz, CDCl_3_) δ 175.4, 171.6, 140.5, 138.2, 130.7, 124.78, 124.6, 124.4, 123.1, 121.7, 62.2, 48.7, 32.4, 28.5 (3C). HRMS (ESI) *m*/*z* calculated for C_16_H_17_NNaO_2_S_2_ [M + Na]^+^ 342.0593, found 342.0589.

#### 5-(Benzo­[*b*]­thiophen-2-ylmethyl)-3-(*tert*-butyl)­thiazolidine-2,4-dione (**26**)

White oil (12.6 mg, yield: 25%). Eluent: PE/EtOAc 95:5. ^1^H NMR (400 MHz, CDCl_3_) δ 7.83–7.75 (m, 1H), 7.73–7.67 (m, 1H), 7.32 (pd, *Js* = 7.2, 1.4 Hz, 2H), 7.13 (d, *J* = 0.9 Hz, 1H), 4.35 (dd, *Js* = 8.8, 3.7 Hz, 1H), 3.70 (ddd, *Js* = 15.1, 3.7, 1.1 Hz, 1H), 3.45 (ddd, *Js* = 14.9, 8.8, 0.8 Hz, 1H), 1.54 (s, 9H). ^13^C­{1H} NMR (101 MHz, CDCl_3_) δ 174.9, 171.5, 139.9, 139.6, 138.7, 124.6, 124.5, 123.9, 123.4, 122.3, 62.3, 49.5, 34.5, 28.5 (3C). HRMS (ESI) *m*/*z* calculated for C_16_H_18_NO_2_S_2_ [M + H]^+^ 320.0773, found 320.0770.

#### 5-((1*H*-Indazol-5-yl)­methyl)-3-(*tert*-butyl)­thiazolidine-2,4-dione (**27**)

White solid (18.1 mg, yield: 39%). Eluent: PE/EtOAc 70:30. ^1^H NMR (400 MHz, CDCl_3_) δ 8.05 (br s, 1H), 7.61 (s, 1H), 7.45 (d, *J* = 8.6 Hz, 1H), 7.29–7.27 (m, 1H), 4.30 (dd, *Js* = 8.7, 3.8 Hz, 1H), 3.53 (dd, *Js* = 14.0, 3.9 Hz, 1H), 3.24 (dd, *Js* = 14.0, 8.7 Hz, 1H), 1.50 (s, 9H). ^13^C­{1H} NMR (101 MHz, CDCl_3_) δ 175.4, 171.6, 139.6, 135.1, 128.8, 128.6, 123.6, 121.6, 109.9, 62.1, 50.4, 39.0, 28.5 (3C). HRMS (ESI) *m*/*z* calculated for C_15_H_18_N_3_O_2_S [M + H]^+^ 304.1114, found 304.1113.

#### *tert*-Butyl 5-((3-(*tert*-butyl)-2,4-dioxothiazolidin-5-yl)­methyl)-1*H*-indole-1-carboxylate (**28**)

Noncrystalline white solid (10.7 mg, yield: 30%). Eluent: PE/EtOAc 90:10. ^1^H NMR (400 MHz, CDCl_3_) δ 8.07 (d, *J* = 8.5 Hz, 1H), 7.59 (d, *J* = 3.8 Hz, 1H), 7.40 (d, *J* = 1.8 Hz, 1H), 7.16 (dd, *Js* = 8.5, 1.8 Hz, 1H), 6.52 (dd, *Js* = 3.7, 0.8 Hz, 1H), 4.29 (dd, *Js* = 9.1, 3.9 Hz, 1H), 3.54 (dd, *Js* = 13.9, 3.9 Hz, 1H), 3.21–3.11 (m, 1H), 1.67 (s, 9H), 1.54 (s, 9H). ^13^C­{1H} NMR (101 MHz, CDCl_3_) δ 175.6, 171.8, 130.9, 130.3, 126.6, 125.7, 121.7, 115.4, 107.2, 83.9, 77.5, 62.1, 50.68, 39.1, 28.5 (3C), 28.3 (3C). HRMS (ESI) *m*/*z* calculated for C_21_H_27_N_2_O_4_S [M + H]^+^ 403.1686, found 403.1681.

#### 3-(*tert*-Butyl)-5-phenethylthiazolidine-2,4-dione (**29**)

White solid (13.4 mg, yield: 42% from pinacol boronic ester, yield: 17% from BF_3_K salt). Eluent: PE/EtOAC 80:20. ^1^H NMR (400 MHz, CDCl_3_) δ 7.30 (tt, *Js* = 6.9, 0.9 Hz, 2H), 7.24–7.15 (m, 3H), 3.93 (dd, *Js* = 9.4, 4.1 Hz, 1H), 2.86–2.64 (m, 2H), 2.47 (dddd, *Js* = 13.5, 9.2, 7.2, 4.2 Hz, 1H), 2.15–2.06 (m, 1H), 1.59 (s, 9H). ^13^C­{1H} NMR (101 MHz, CDCl_3_) δ 176.2, 171.7, 139.7, 128.8 (2C), 128.7 (2C), 126.7, 60.5, 47.8, 35.2, 32.9, 28.6 (3C). HRMS (ESI) *m*/*z* calculated for C_15_H_19_NNaO_2_S [M + Na]^+^ 300.1029, found 300.1024.

#### 3-(*tert*-Butyl)-5-(3,4,5-trimethoxyphenethyl)­thiazolidine-2,4-dione (**30**)

White solid (23.6 mg, yield: 52%). Eluent: PE/EtOAc 95:5. ^1^H NMR (400 MHz, CDCl_3_) δ 6.38 (s, 2H), 3.94 (dd, *Js* = 9.4, 4.1 Hz, 1H), 3.84 (s, 6H), 3.81 (s, 3H), 2.76–2.60 (m, 2H), 2.50–2.41 (m, 1H), 2.15–2.05 (m, 1H), 1.58 (s, 9H). ^13^C­{1H} NMR (101 MHz, CDCl_3_) δ 176.1, 171.7, 153.5 (2C), 136.7, 135.4, 105.5 (2C), 62.2, 60.9, 56.2 (2C), 47.7, 35.1, 33.4, 28.6 (3C). HRMS (ESI) *m*/*z* calculated for C_18_H_26_NO_5_S [M + H]^+^ 368.1526, found 368.1522.

#### 5-(2-(Benzo­[*d*]­[1,3]­dioxol-5-yl)­ethyl)-3-(*tert*-butyl)­thiazolidine-2,4-dione (**31**)

White solid (15.9 mg, yield: 36%). Eluent: PE/EtOAc 98:2. ^1^H NMR (400 MHz, CDCl_3_) δ 6.73 (d, *J* = 7.9 Hz, 1H), 6.67 (d, *J* = 1.8 Hz, 1H), 6.62 (dd, *Js* = 7.9, 1.8 Hz, 1H), 5.93 (s, 2H), 3.91 (dd, *Js* = 9.4, 4.2 Hz, 1H), 2.75–2.59 (m, 2H), 2.47–2.38 (m, 1H), 2.10–2.01 (m, 1H), 1.59 (s, 9H). ^13^C­{1H} NMR (101 MHz, CDCl_3_) δ 176.2, 171.8, 148.0, 146.4, 133.4, 121.6, 109.1, 108.5, 101.1, 62.1, 47.7, 35.4, 32.8, 28.6 (3C). HRMS (ESI) *m*/*z* calculated for C_16_H_19_NNaO_4_S [M + Na]^+^ 344.0927, found 344.0922.

#### 3-(*tert*-Butyl)-5-(indolin-2-ylmethyl)­thiazolidine-2,4-dione (**32**)

Noncrystalline white solid (39.1 mg, yield: 83%, product isolated as a mixture 1:1 of diastereomers; in the NMR spectra the presence of different conformers was observed). Eluent: PE/EtOAc 88:12. ^1^H NMR (400 MHz, DMSO-*d*_6_) δ 7.03–6.97 (m, 3H_a+b_), 6.90 (td, *Js* = 7.6, 1.3 Hz, 1H_a_), 6.63–6.56 (m, 2H_a+b_)_,_ 6.55–6.50 (m, 1H_a_), 6.48 (d, *J* = 7.7 Hz, 1H_b_), 5.81 (d, *J* = 3.2 Hz, 1H_a_), 5.66 (d, *J* = 3.3 Hz, 1H_b_), 4.66 (dd, *Js* = 7.3, 4.8 Hz, 1H_a_), 4.46–4.34 (m, 2H_a+b_), 4.03–3.94 (m, 1H_b_), 3.87–3.78 (m, 1H_a_), 3.70 (dd, *Js* = 15.0, 4.8 Hz, 1H_a_), 3.19–3.11 (m, 1H_b_), 3.10–3.02 (m, 1H_b_), 2.73 (dd, *Js* = 15.9, 7.8 Hz, 1H_a_), 2.65 (dd, *Js* = 15.9, 8.9 Hz, 1H_a_), 2.13–2.05 (m, 1H_b_), 2.02–1.93 (m, 1H_b_), 1.53 (s, 9H_a_), 1.50 (s, 9H_b_). ^13^C­{1H} NMR (101 MHz, CDCl_3_) δ 176.1 (C_a_), 174.9 (C_b_), 172.2 (C_a_), 171.6 (C_b_), 150.5 (C_a_), 150.0 (C_b_), 128.2 (C_a_), 128.2 (C_b_), 127.9 (C_a_), 127.7 (C_a_), 124.8 (C_a_), 119.4 (C_a_), 119.4 (C_b_), 109.9 (C_a_), 108.1 (C_b_), 62.4 (C_a_), 61.9 (C_b_), 57.7 (C_a_), 50.8 (C_b_), 47.5 (C_a_), 45.5 (C_b_), 44.0 (C_a_), 40.1 (C_b_), 36.9 (C_a_), 36.1 (C_b_), 34.4 (C_a_), 28.6 (3C_a_), 28.5 (3C_b_). HRMS (ESI) *m*/*z* calculated for C_16_H_21_N_2_O_2_S [M + H]^+^ 305.1318, found 305.1313.

#### 3-(*tert*-Butyl)-5-(2-(phenylamino)­ethyl)­thiazolidine-2,4-dione (**33**)

Noncrystalline white solid (38.3 mg, yield: 79%). Eluent: PE/EtOAc 87:13. ^1^H NMR (400 MHz, CDCl_3_) δ 7.23–7.13 (m, 2H), 6.73 (tt, *J* = 7.3, 1.1 Hz, 1H), 6.61 (dq, *Js* = 7.0, 1.5, 1.1 Hz, 2H), 4.14 (ddd, *Js* = 8.0, 5.9, 2.6 Hz, 1H), 3.42–3.33 (m, 1H), 3.29 (dt, *Js* = 13.0, 6.4 Hz, 1H), 2.41 (dtd, *Js* = 14.2, 6.4, 4.6 Hz, 1H), 2.25–2.15 (m, 1H), 1.58 (s, 9H). ^13^C­{1H} NMR (101 MHz, CDCl_3_) δ 176.2, 171.7, 147.7, 129.5 (2C), 118.1, 113.1 (2C), 62.2, 46.2, 41.3, 32.9, 28.5 (3C). HRMS (ESI) *m*/*z* calculated for C_15_H_21_N_2_O_2_S [M + H]^+^ 293.1318, found 293.1312.

#### 5-(3,4,5-Trimethoxybenzyl)­thiazolidine-2,4-dione (**34**)

Off-white solid (18.9 mg, yield: 90%). Eluent: PE/EtOAc 50:50. ^1^H NMR (400 MHz, CDCl_3_) δ 6.44 (s, 2H), 4.75 (s, 1H), 4.50 (dd, *Js* = 10.1, 3.7 Hz, 1H), 3.85 (s, 6H), 3.83 (s, 3H), 3.55–3.42 (m, 1H), 3.04 (dd, *Js* = 14.0, 10.1 Hz, 1H). ^13^C­{1H} NMR (101 MHz, CDCl_3_) δ 174.2, 170.5, 153.6 (2C), 137.5, 131.7, 106.2 (2C), 61.01, 56.3 (2C), 53.8, 39.3. HRMS (ESI) *m*/*z* calculated for C_13_H_14_NO_5_S [M-H]^−^ 296.0596, found 296.0595.

#### 5-(3-(Methylthio)­benzyl)­thiazolidine-2,4-dione (**35**)

Yellow solid (12.2 mg, yield: 49%). Eluent: no further purification needed. ^1^H NMR (400 MHz, CDCl_3_) δ 7.31–7.24 (m, 1H), 7.19 (dt, *J* = 8.0, 1.5 Hz, 1H), 7.13 (t, *J* = 1.9 Hz, 1H), 7.02 (dt, *Js* = 7.5, 1.5 Hz, 1H), 4.54 (dd, *Js* = 9.9, 3.9 Hz, 1H), 3.54 (dd, *Js* = 14.1, 3.9 Hz, 1H), 3.12 (dd, *Js* = 14.0, 9.9 Hz, 1H), 2.50 (s, 3H). ^13^C­{1H} NMR (101 MHz, CDCl_3_) δ 174.1, 170.4, 139.6, 136.7, 129.4, 127.1, 125.8, 125.8, 53.4, 38.7, 15.8. HRMS (ESI) *m*/*z* calculated for C_11_H_10_NO_2_S_2_ [M-H]^−^ 252.0158, found 252.0157.

#### 5-(4-Aminobenzyl)­thiazolidine-2,4-dione (**36**)

Off-white oil (8 mg, yield: 45%). Eluent: DCM/MeOH 99:1. ^1^H NMR (400 MHz, Acetone-*d*_6_) δ 6.98 (d, *J* = 8.4 Hz, 2H), 6.65–6.58 (m, 2H), 4.71 (dd, *Js* = 9.4, 4.1 Hz, 1H), 3.34 (dd, *Js* = 14.1, 4.1 Hz, 1H), 3.01 (dd, *Js* = 14.2, 9.4 Hz, 1H). ^13^C­{1H} NMR (101 MHz, Acetone-*d*_6_) δ 175.6, 171.6, 130.8 (2C), 125.3, 120.4, 115.2 (2C), 54.7, 38.2. HRMS (ESI) *m*/*z* calculated for C_10_H_11_N_2_O_2_S [M + H]^+^ 223.0536, found 223.0534.

#### 5-((1*H*-Indazol-5-yl)­methyl)­thiazolidine-2,4-dione (**37**)

Off-white solid (6 mg, yield: 49%). Eluent: PE/EtOAc 50:50. ^1^H NMR (400 MHz, *d*_4_-MeOH) δ 8.01 (d, *J* = 1.0 Hz, 1H), 7.66 (d, *J* = 1.6 Hz, 1H), 7.53–7.47 (m, 1H), 7.33 (dd, *Js* = 8.6, 1.6 Hz, 1H), 4.78 (dd, *Js* = 9.1, 4.1 Hz, 1H), 3.58 (dd, *Js* = 14.2, 4.1 Hz, 1H), 3.26 (dd, *Js* = 14.2, 9.2 Hz, 1H). HRMS (ESI) *m*/*z* calculated for C_11_H_10_N_3_O_2_S [M + H]^+^ 248.0488, found 248.0487.

#### 5-(2-(Phenylamino)­ethyl)­thiazolidine-2,4-dione (**38**)

Light brown solid (13.4 mg, yield: 55%). Eluent: PE/EtOAc 80:20. ^1^H NMR (400 MHz, Acetone-*d*_6_) δ 7.12 (dd, *Js* = 8.6, 7.3 Hz, 2H), 6.67 (dt, *Js* = 7.8, 1.1 Hz, 2H), 6.65–6.57 (m, 1H), 4.62 (dd, *Js* = 9.3, 4.4 Hz, 1H), 3.42–3.35 (m, 2H), 2.53 (dtd, *Js* = 14.1, 7.0, 4.5 Hz, 1H), 2.30–2.16 (m, 1H). ^13^C­{1H} NMR (101 MHz, Acetone-*d*_6_) δ 176.4, 171.7, 149.5, 129.9 (2C), 117.6, 113.4 (2C), 50.2, 41.9, 33.3. HRMS (ESI) *m*/*z* calculated for C_11_H_13_N_2_O_2_S [M + H]^+^ 237.0692, found 237.0691.

### Synthesis of Pioglitazone (**44**)

#### Step 1: Mitsunobu Reaction for the Synthesis of **41**

A solution of 4-(4,4,5,5-tetramethyl-1,3,2-dioxaborolan-2-yl)­phenol **39** (250 mg, 1.65 mmol, 1 equiv) in dry THF (12 mL) was cooled to 0 °C. PPh_3_ (563 mg, 2.15 mmol, 1.3 equiv), **40** (1.65 mmol, 1 equiv) and DIAD (434.5 mg, 2.15 mmol, 1.3 equiv) were added sequentially. The reaction mixture was stirred at room temperature for 3 h under a nitrogen atmosphere. Upon completion, the solvent was evaporated under reduced pressure. The residue was washed with a saturated 2N NaOH solution and extracted with EtOAc. The combined organic layers were dried over anhydrous Na_2_SO_4_, filtered, and concentrated under *vacuum*. The crude product (300 mg, yield: 57%) proceeded to the next step without further purification.

##### **5**-Ethyl-2-(2-(4-(4,4,5,5-tetramethyl-1,3,2-dioxaborolan-2-yl)­phenoxy)­ethyl)­pyridine (**41**)

White solid (300 mg, yield: 57%). Eluent: no further purification needed. ^1^H NMR (400 MHz, CDCl_3_) δ 8.38 (d, *J* = 2.3 Hz, 1H), 7.72 (d, *J* = 8.6 Hz, 2H), 7.47 (dd, *Js* = 7.9, 2.4 Hz, 1H), 7.20 (d, *J* = 7.9 Hz, 1H), 6.90–6.85 (m, 2H), 4.35 (t, *J* = 6.6 Hz, 2H), 3.24 (t, *J* = 6.7 Hz, 2H), 2.63 (q, *J* = 7.6 Hz, 2H), 1.32 (d, *J* = 1.2 Hz, 15H). ^13^C­{1H} NMR (101 MHz, CDCl_3_) δ 161.5, 155.7, 148.8, 137.4, 136.9, 136.6 (2C), 136.3, 123.7, 114.0 (2C), 83.7 (2C), 67.1, 37.4, 25.8, 24.9 (4C), 15.4. HRMS (ESI) *m*/*z* calculated for C_21_H_29_BNO_3_ [M + H]^+^ 354.2235, found 354.2230.

#### Step 2: Hydrolysis Reaction for the Synthesis of Compound **42**

Compound **41** (300 mg, 0.85 mmol), NaIO_4_ (1.27 mmol, 1.5 equiv), and CH_3_COONH_4_ (1.27 mmol, 1.5 equiv) were combined and dissolved in acetone (750 μL) and water (750 μL) in a round-bottomed flask. The mixture was stirred vigorously and heated in an oil bath at 65 °C for 5 h. The slurry was filtered, and the reaction was concentrated under reduced pressure. The aqueous solution was extracted with EtOAc, then the organic phase was washed with brine, dried over anhydrous Na_2_SO_4_ and concentrated to obtain 209.7 mg of **42** as a light brown solid (yield: 90%).

##### (4-(2-(5-Ethylpyridin-2-yl)­ethoxy)­phenyl)­boronic Acid (**42**)

Light brown solid (209.7 mg, yield: 90%). Eluent: no further purification needed. ^1^H NMR (400 MHz, acetone-d_6_) δ 8.38 (d, *J* = 2.3 Hz, 1H), 7.80 (d, *J* = 8.6 Hz, 2H), 7.55 (dd, *Js* = 7.9, 2.4 Hz, 1H), 7.27 (d, *J* = 7.9 Hz, 1H), 6.91 (d, *J* = 8.7 Hz, 2H), 4.40 (t, *J* = 6.8 Hz, 2H), 3.19 (t, *J* = 6.8 Hz, 2H), 2.63 (q, *J* = 7.6 Hz, 2H), 1.22 (t, *J* = 1.2 Hz, 3H). HRMS (ESI) *m*/*z* calculated for C_15_H_19_BNO_3_ [M + H]^+^ 272.1452, found 272.1448.

#### Step 3: See General Procedure A

##### 3-(*tert*-Butyl)-5-(4-(2-(5-ethylpyridin-2-yl)­ethoxy)­benzyl)­thiazolidine-2,4-dione (**43**)

Off-white solid (30.1 mg, yield: 56%). Eluent: PE/EtOAc 50:50. ^1^H NMR (400 MHz, CDCl_3_) δ 8.38 (d, *J* = 2.3 Hz, 1H), 7.45 (dd, *Js* = 7.9, 2.4 Hz, 1H), 7.17 (d, *J* = 7.8 Hz, 1H), 7.13–7.08 (m, 2H), 6.87–6.80 (m, 2H), 4.31 (t, *J* = 6.7 Hz, 2H), 4.19 (dd, *Js* = 8.9, 3.8 Hz, 1H), 3.33 (dd, *Js* = 14.0, 3.8 Hz, 1H), 3.21 (td, *Js* = 6.7, 3.8 Hz, 2H), 3.10–2.97 (m, 1H), 2.62 (q, *J* = 7.6 Hz, 2H), 1.52 (s, 9H), 1.22 (t, *J* = 1.2 Hz, 3H). ^13^C­{1H} NMR (101 MHz, CDCl_3_) δ 175.3, 171.7, 158.3, 155.6, 149.1, 137.1, 135.8, 130.5 (2C), 127.7, 123.2, 114.7 (2C), 67.4, 61.9, 50.2, 38.2, 37.6, 28.4 (3C), 25.7, 15.4. HRMS (ESI) *m*/*z* calculated for C_23_H_29_N_2_O_3_S [M + H]^+^ 413.1893, found 413.1886.

#### Step 4: See General Procedure B

##### 5-(4-(2-(5-Ethylpyridin-2-yl)­ethoxy)­benzyl)­thiazolidine-2,4-dione (**44**)

White solid (16.5 mg, yield: 64%). Eluent: DCM/MeOH 96:4. ^1^H NMR (400 MHz, DMSO-*d*_6_) δ 12.00 (s, 1H), 8.36 (d, *J* = 2.3 Hz, 1H), 7.57 (dd, *Js* = 8.0, 2.4 Hz, 1H), 7.27 (d, *J* = 7.9 Hz, 1H), 7.21–7.08 (m, 2H), 6.86 (d, *J* = 8.7 Hz, 2H), 4.85 (dd, *Js* = 9.1, 4.3 Hz, 1H), 4.30 (t, *J* = 6.7 Hz, 2H), 3.29 (dd, *Js* = 10.9, 3.4 Hz, 1H), 3.12 (t, *J* = 6.7 Hz, 2H), 3.04 (dd, *Js* = 14.2, 9.1 Hz, 1H), 2.58 (q, *J* = 7.6 Hz, 2H), 1.17 (t, *J* = 7.6 Hz, 3H). ^13^C­{1H} NMR (101 MHz, DMSO-*d*_6_) δ 176.2, 172.0, 157.5, 155.5, 148.6, 136.7, 135.7, 130.4 (2C), 128.7, 123.1, 114.4 (2C), 66.7, 53.2, 36.8, 36.4, 24.9, 15.4. HRMS (ESI) *m*/*z* calculated for C_19_H_20_N_2_O_3_S [M + H]^+^ 357.1267, found 357.1267.

### Synthesis of the Deuterated Compound **45**

An oven-dried 7 mL clear vial equipped with a magnetic stirring bar was charged with (4′-ethoxy-[1,1′-biphenyl]-4-yl)­boronic acid **4** (25 mg), PC (2.5 mol %), Lewis base (0.25 equiv) and *tert*-butyl 5-methylene-2,4-dioxothiazolidine-3-carboxylate **3** (3 equiv). The vial was sealed with a screw cap with septum and 3 cycles *vacuum*/nitrogen were performed. A solvent mixture of acetone/*d*_4_-MeOH (0.88 mL, purged with nitrogen for 15 min) was added. The tube was irradiated in a SynLED Parallel Photoreactor (450 nm) for 16 h. The reaction was concentrated in vacuo, reconstituted in DCM, and purified through flash chromatography (PE/EtOAc 98:2).

#### 3-(*tert*-Butyl)-5-((4′-ethoxy-[1,1′-biphenyl]-4-yl)­methyl)­thiazolidine-2,4-dione-5-d (**45**)

White solid (29.3 mg, yield: 74%). Eluent: Cyclohexane/EtOAc 99:1. [D] = 90%. ^1^H NMR (400 MHz, CDCl_3_) δ 7.53–7.46 (m, 4H), 7.27 (d, *J* = 7.4 Hz, 2H), 6.96 (d, *J* = 8.7 Hz, 2H), 4.08 (q, *J* = 7.0 Hz, 2H), 3.45 (d, *J* = 13.9 Hz, 1H), 3.12 (d, *J* = 13.9 Hz, 1H), 1.53 (s, 9H), 1.44 (t, *J* = 7.0 Hz, 3H). ^13^C­{1H} NMR (101 MHz, CDCl_3_) δ 175.4, 171.8, 158.7, 140.2, 134.2, 133.1, 130.0 (2C), 128.1 (2C), 127.0 (2C), 114.9 (2C), 63.7, 62.1, 50.0, 38.7, 28.5 (3C), 15.0. HRMS (ESI) *m*/*z* calculated for C_22_H_24_DKNO_3_S [M+K]^+^ 423.1249, found 423.1247.

## Supplementary Material



## Data Availability

The data underlying this study are available in the published article and its online Supporting Information.

## Notes

Experimental procedures, characterization data, and copies of ^1^H and ^13^C­{1 H} spectra (PDF)

## §

Process R&D, Procos S.P.A., Cameri, Novara 28062, Italy
