# Supplementary material for: Sustainable Photocatalytic Synthesis of Glitazones via Riboflavin Tetraacetate
Source: J Org Chem. 2025 Jul 5;90(28):10064–73. doi: 10.1021/acs.joc.5c01306 (PMC12281567; doi:10.1021/acs.joc.5c01306)

Supporting Information

Sustainable Photocatalytic Synthesis of Glitazones *via* Riboflavin Tetraacetate

Sarah Jane Rezzi[a], Marco Koten[a], Rita Maria Concetta Di Martino[a], Gianluca Papeo[b], Tracey Pirali[a]* and Marina Caldarelli[b]

[a] Department of Pharmaceutical Sciences, Università del Piemonte Orientale, Largo Donegani 2, 28100, Novara, Italy

[b] Nerviano Medical Sciences Srl, viale Pasteur 10, 20014 Nerviano (Milano), Italy

Present Addresses

†Process R&D, Procos S.P.A., Cameri, Novara 28062, Italy

Table of Contents

1. Additional figures 2

2. Experimental procedures 2

3. Characterization data of compounds 4

4. Copies of NMR spectra 5

1. Additional figures

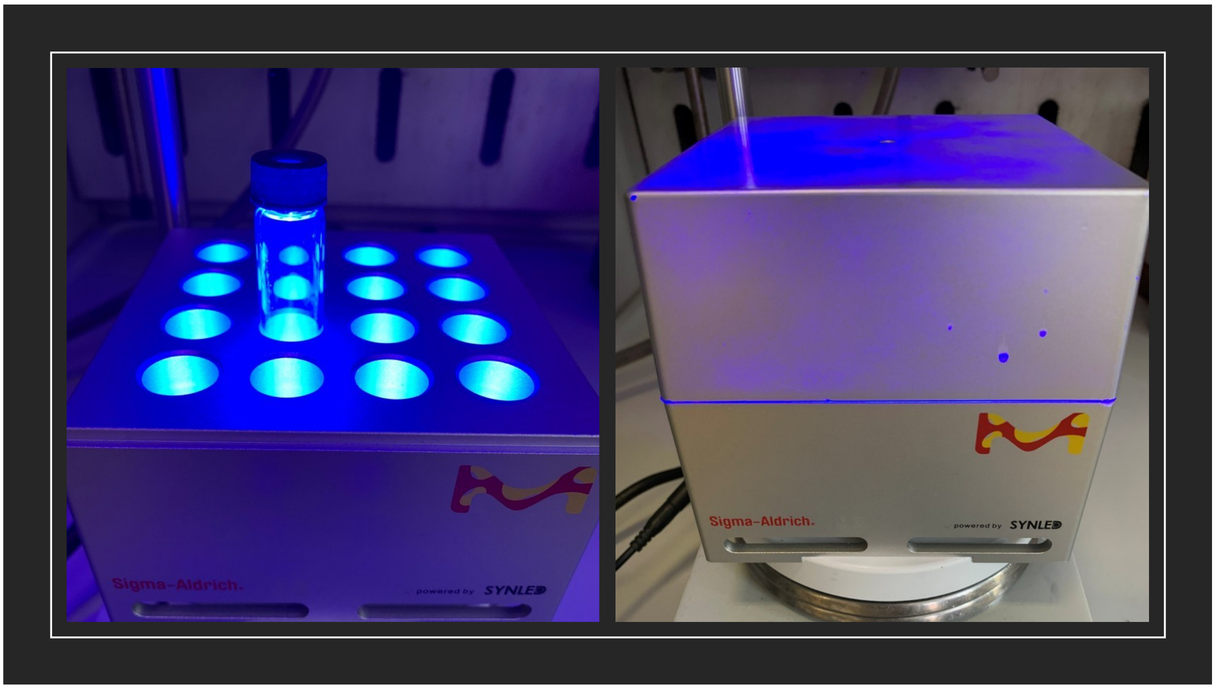

Figure S1. SynLED Parallel Photoreactor 2.0

2. Experimental procedures

Procedure A: synthesis of 3-(*tert*-butyl)thiazolidine-2,4-dione

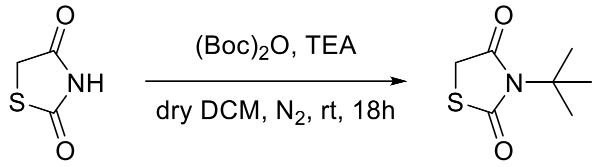

Under nitrogen atmosphere, a solution of thiazolidine-2,4-dione (TZD, 20.0 g, 0.17 mol, 1 equiv) in dry DCM (250 mL) was cooled to 0 °C. TEA (47.6 mL, 0.34 mol, 2 equiv) was added dropwise, followed by the portionwise addition of Boc anhydride (46.58 g, 0.23 mol, 2.4 equiv). The reaction was allowed to warm up to room temperature and stirred overnight. Upon completion, the reaction mixture was diluted with 125 mL of 3 M aqueous HCl and extracted twice with DCM. The combined organic layers were dried over anhydrous Na_2SO_4, filtered and concentrated under *vacuum*. The reaction crude was purified through flash chromatography (PE/EtOAc 98:2).

Procedure B: synthesis of 3-(*tert*-butyl)-5-methylenethiazolidine-2,4-dione

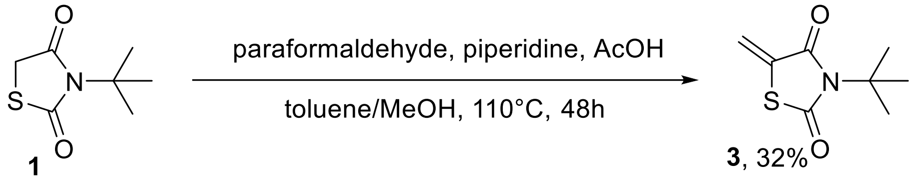

To a stirred solution of 3-(*tert*-butyl)thiazolidine-2,4-dione **1** (4 g, 23.12 mmol, 1 equiv) in dry toluene (59.4 mL) and dry MeOH (1.68 mL), paraformaldehyde (3.33 g, 69.36 mmol, 3 equiv) was added. Then piperidine (0.23 mL, 2.31 mmol, 0.1 equiv) and glacial acetic acid (0.20 mL, 3.46 mmol, 0.15 equiv) were added. The round-bottom flask was fitted with a Dean-Stark apparatus and the reaction was heated in an oil bath at reflux for two days. Upon completion the reaction crude was diluted with water and extracted with EtOAc. The combined organic layers were dried over anhydrous Na_2SO_4, filtered and the solvent was evaporated under *vacuum*. The reaction crude was purified through flash chromatography (PE/EtOAc 99:1).

Procedure C: optimization of the photoredox reaction

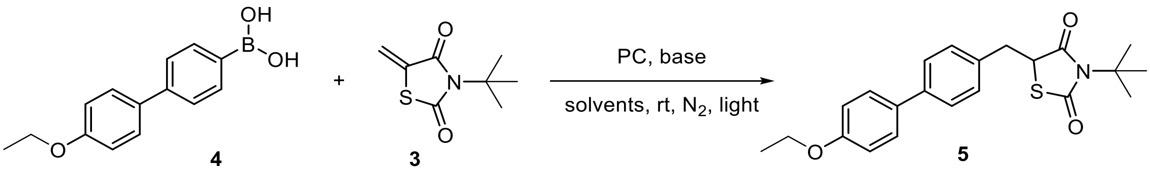

An oven-dried 7 mL clear vial equipped with a magnetic stirring bar was charged with (4'-ethoxy-[1,1'-biphenyl]-4-yl)boronic acid **4** (25 mg), PC (2.5 mol%, unless stated otherwise, see Table 1), Lewis base (0.25 equiv) and *tert*-butyl 5-methylene-2,4-dioxothiazolidine-3-carboxylate **3** (3 equiv). The vial was sealed with a screw cap with septum and 3 cycles *vacuum*/nitrogen were performed. A solvent mixture (0.90 mL, purged with nitrogen for 15 minutes) was added. The tube was irradiated in a SynLED Parallel Photoreactor (450 nm) for 16 hours (unless stated otherwise, see Table 1). The reaction was concentrated in vacuo, reconstituted in DCM, and purified through flash chromatography (PE/EtOAc 98:2).
See Table 1.

Procedure D: synthesis of compound 5 in a 1 mmol scale

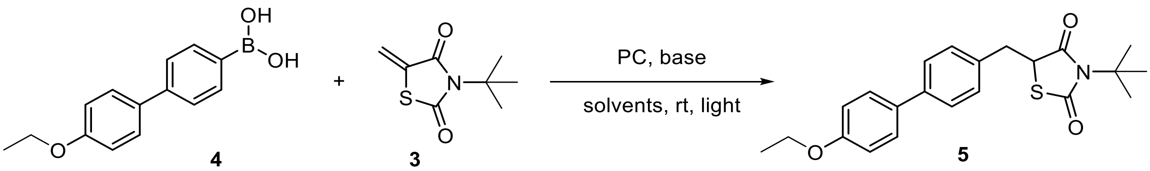

An oven-dried 7 mL clear vial equipped with a magnetic stirring bar was charged with (4'-ethoxy-[1,1'-biphenyl]-4-yl)boronic acid **4** (242 mg), PC (2.5 mol%), Lewis base (0.25 equiv) and *tert*-butyl 5-methylene-2,4-dioxothiazolidine-3-carboxylate **3** (3 equiv). The vial was sealed with a screw cap with septum and 3 cycles *vacuum*/nitrogen were performed. A solvent mixture (7 mL, purged with nitrogen for 15 minutes) was added. The tube was irradiated in a

SynLED Parallel Photoreactor (450 nm) for 16 hours. The reaction was concentrated in vacuo, reconstituted in DCM, and purified through flash chromatography (PE/EtOAc 98:2) to obtain a white solid (243 mg, 63%).

3. Characterization data of compounds

3-(*tert*-Butyl)thiazolidine-2,4-dione

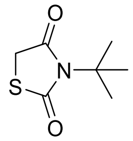

1: pale-yellow oil (19.76 g, yield: 53%). **Eluent**: PE/EtOAc 98:2. **^{1}H NMR** (400 MHz, $CDCl_3$) δ 3.79 (s, 2H), 1.61 (s, 9H). **^{13}C{1H} NMR** (101 MHz, $CDCl_3$) δ 172.9, 172.5, 62.2, 33.3, 28.4 (3C). **IR** (neat) 3421, 2977, 2938, 1753, 1673, 1310, 1136, 1037, 864, 7873, 510 cm^{-1}.

2-(*tert*-butoxy)thiazol-4(5*H*)-one

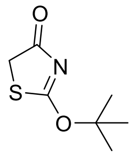

2: yellow oil (3.00 g, yield: 10%). **Eluent**: PE/EtOAc 98:2. **^{1}H NMR** (400 MHz, $CDCl_3$) δ 4.06 (s, 2H), 1.61 (s, 9H). **^{13}C{1H} NMR** (101 MHz, $CDCl_3$) δ 186.8, 185.4, 89.5, 40.1, 27.6.

3-(*tert*-Butyl)-5-methylenethiazolidine-2,4-dione

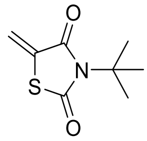

3: white crystalline solid (1.45 g, yield: 32%). **Eluent**: PE/EtOAc 99:1. **^{1}H NMR** (400 MHz, $CDCl_3$) δ 6.50 (d, *J* = 1.2 Hz, 1H), 5.66 (d, *J* = 1.2 Hz, 1H), 1.66 (s, 9H). **^{13}C{1H} NMR** (101 MHz, $CDCl_3$) δ 168.1, 166.3, 132.1, 118.0, 62.5, 28.1 (3C). **IR** (neat) 3412, 3127, 2965, 2939, 1754, 1675, 1609, 1300, 1258, 1181, 1129, 1028, 789 cm^{-1}. **HRMS** (ESI) *m/z* calculated for $C_8H_{11}NNaO_2S$ [M+Na]$^+$ 208.0403, found 208.0406.

4. Copies of NMR spectra

1, ^{1}H NMR (400 MHz, $CDCl_3$)

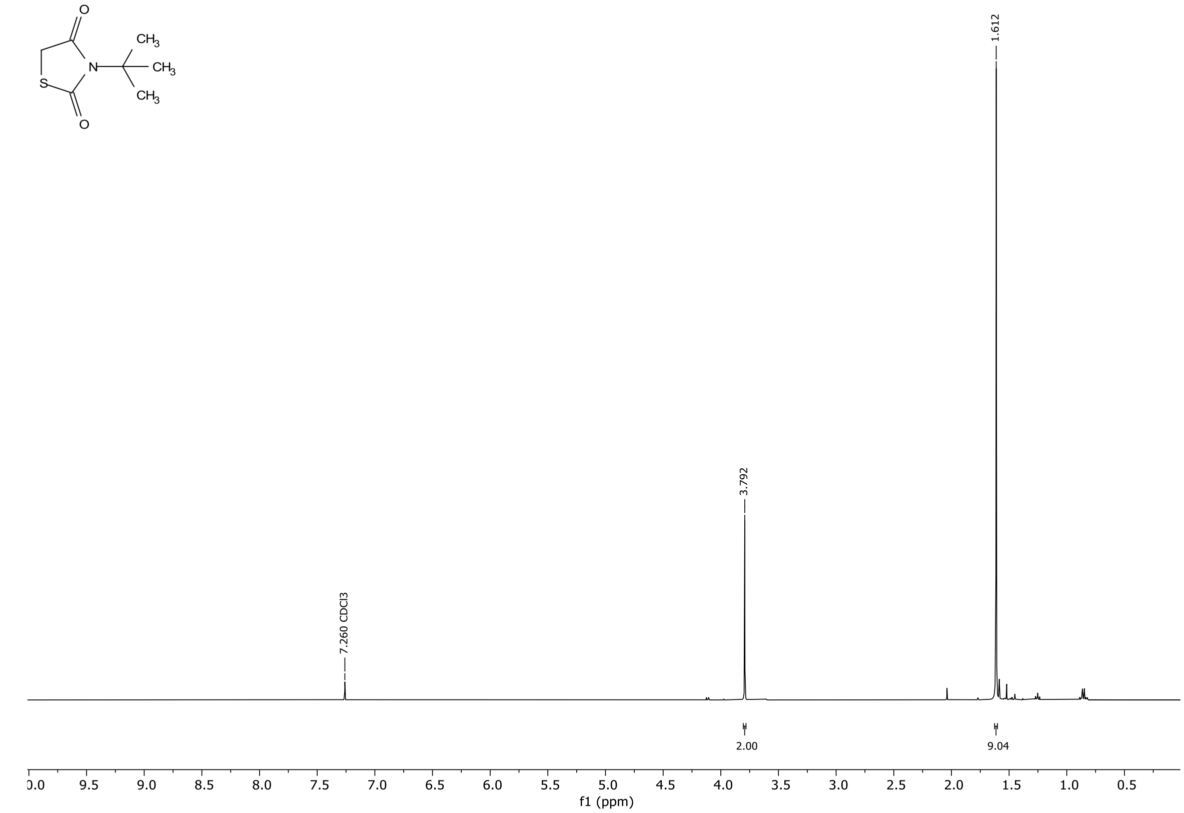

1, **^{13}C{1H} NMR** (101 MHz, $CDCl_3$)

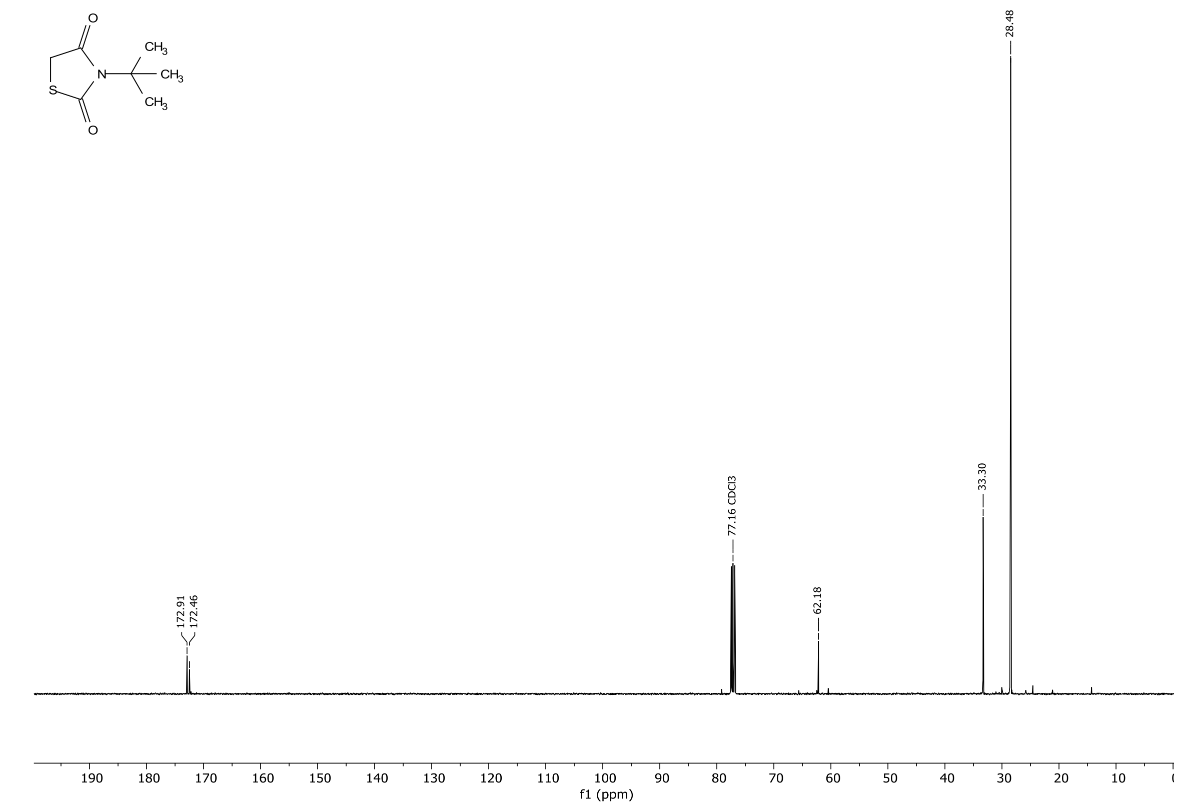

2, ^{1}H NMR (400 MHz, $CDCl_3$)

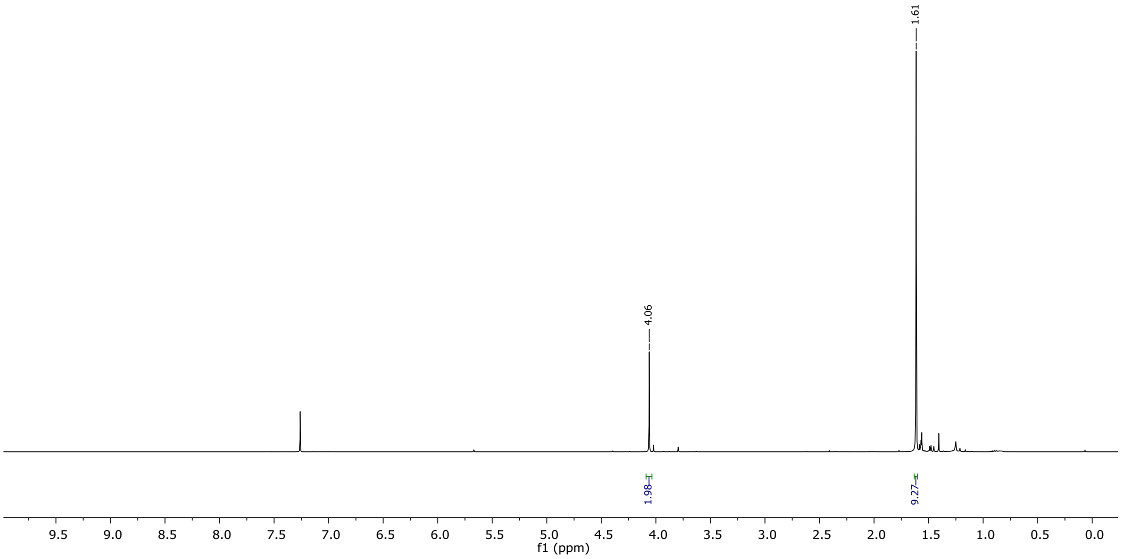

2, **^{13}C{1H} NMR** (101 MHz, $CDCl_3$)

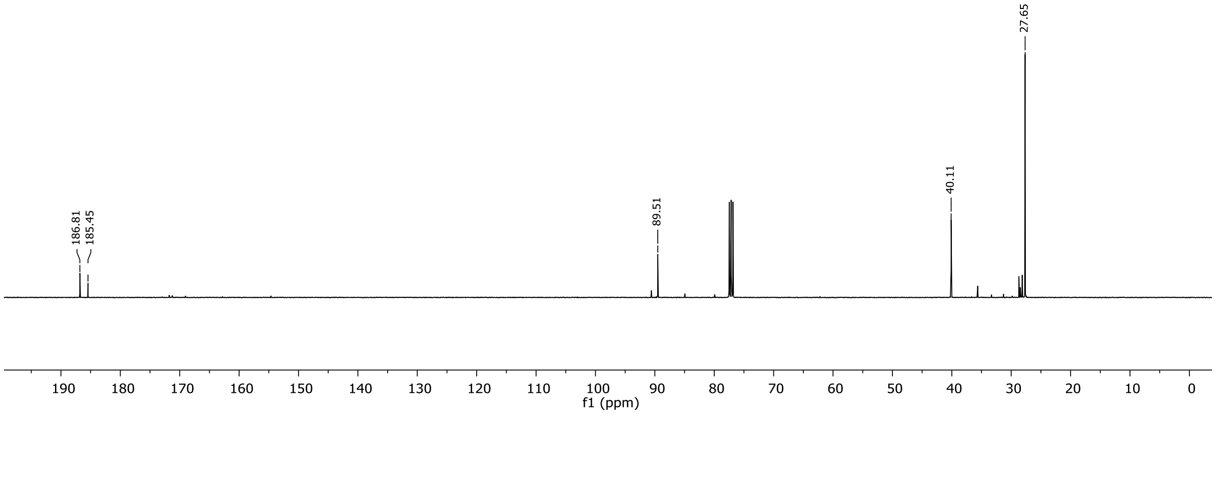

3, ^{1}H NMR (400 MHz, $CDCl_3$)

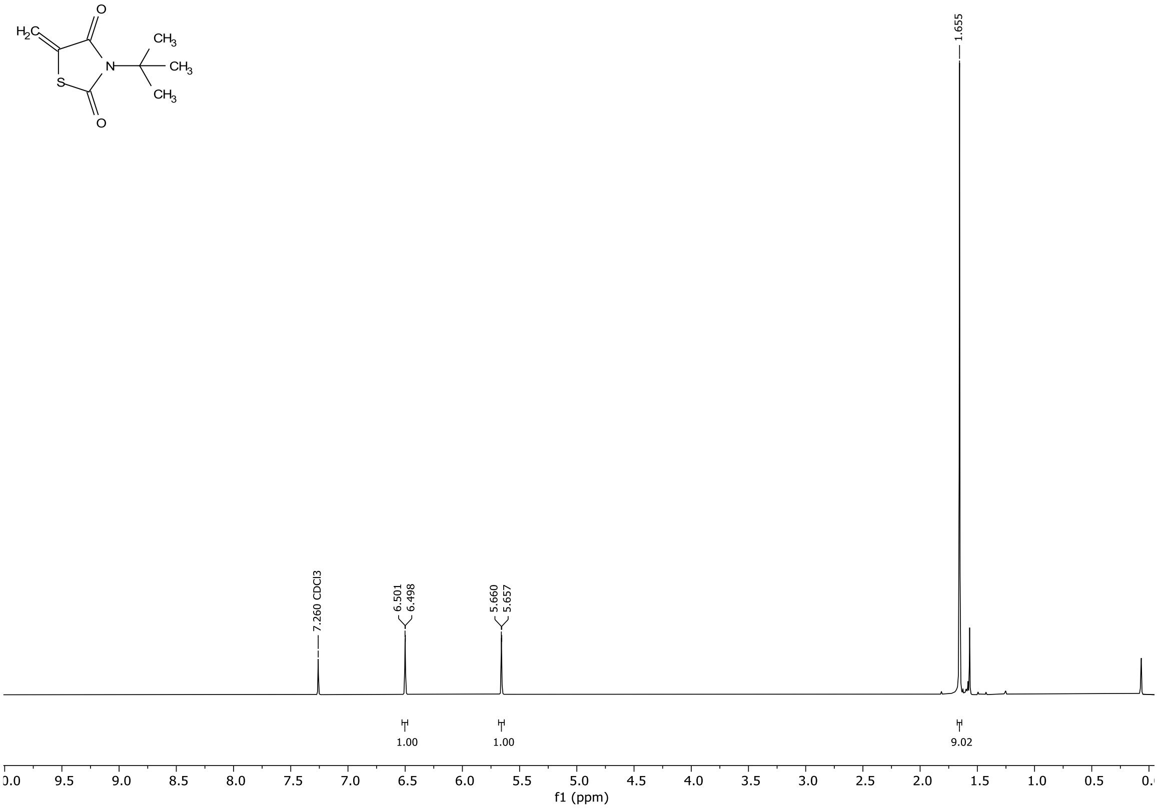

3, **^{13}C{1H} NMR** (101 MHz, $CDCl_3$)

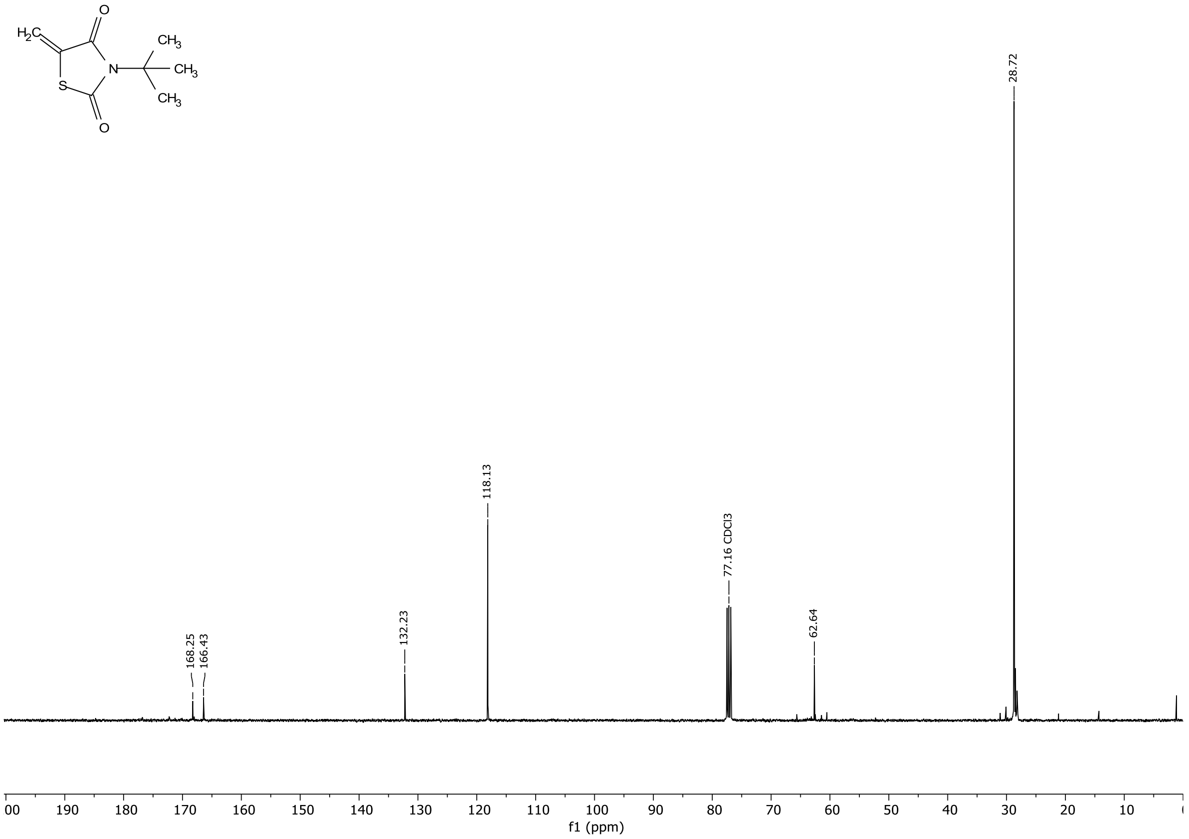

5, ^{1}H NMR (400 MHz, $CDCl_3$)

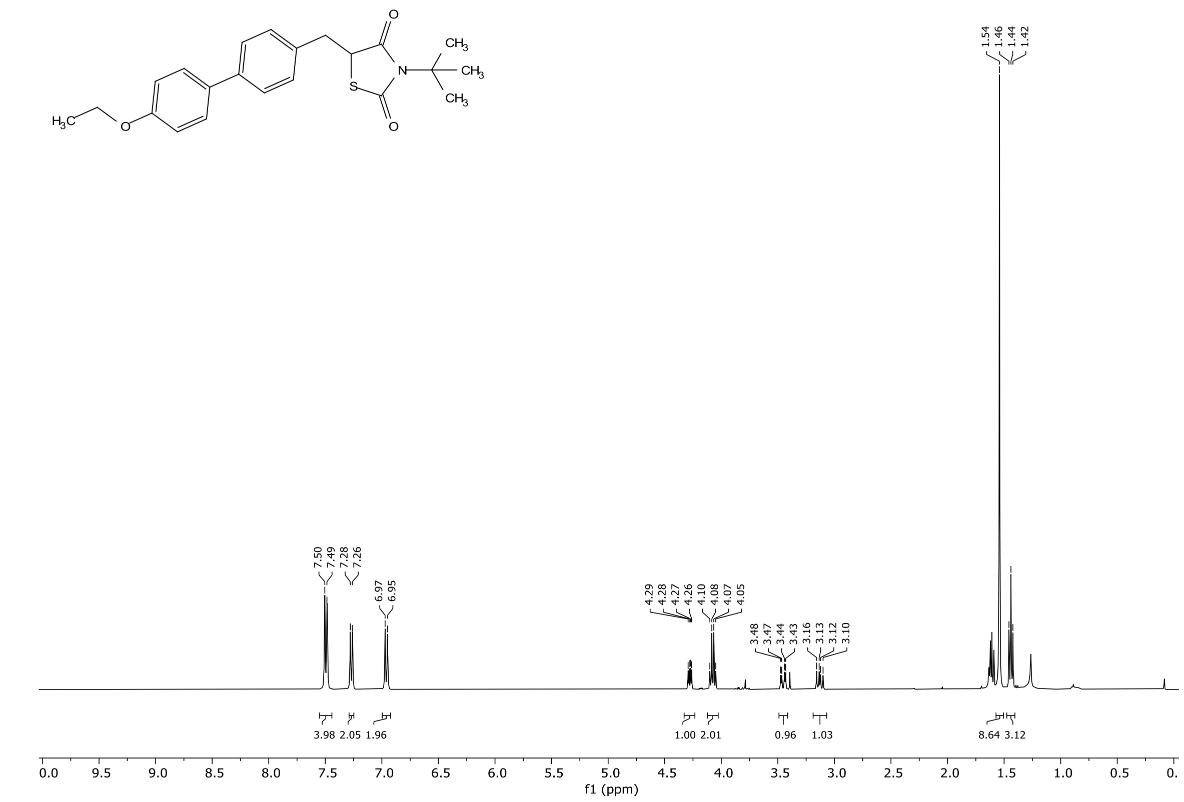

5, **^{13}C{1H} NMR** (101 MHz, $CDCl_3$)

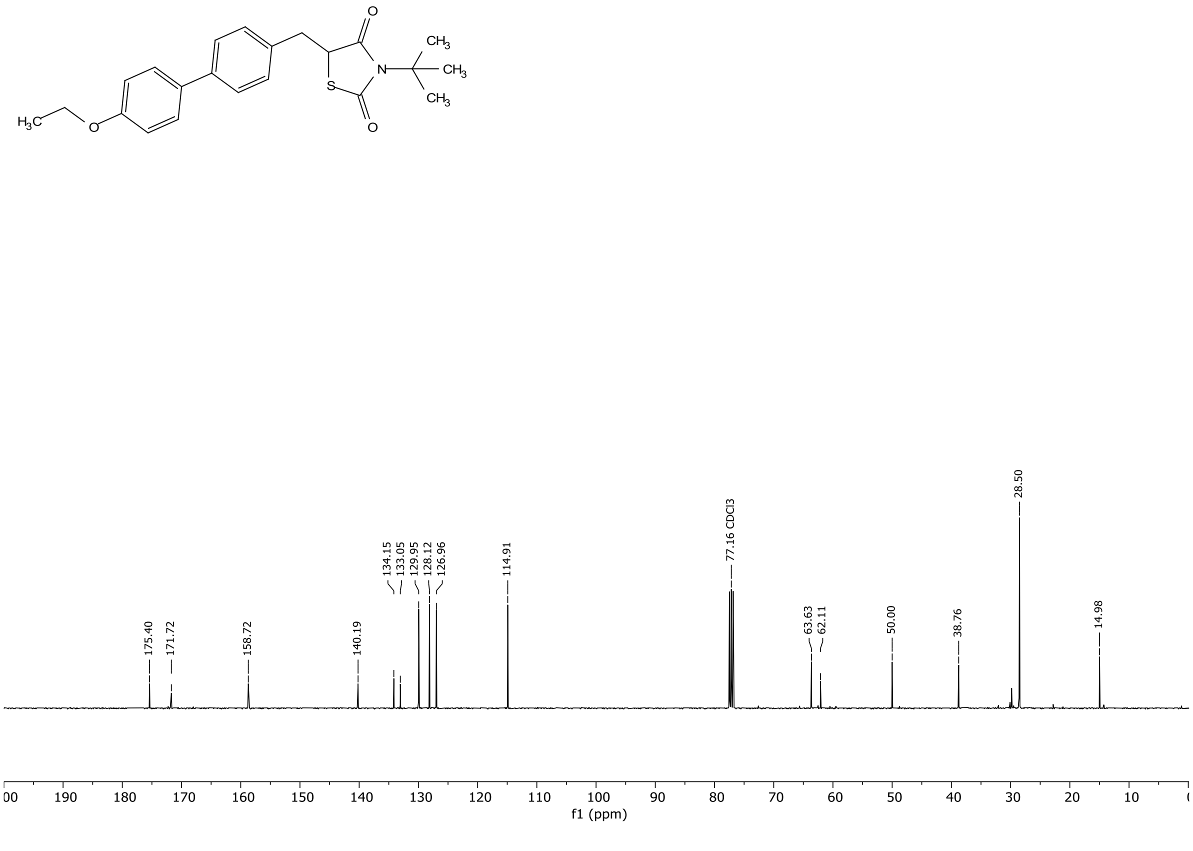

6, ¹H NMR (400 MHz, $CDCl_3$)

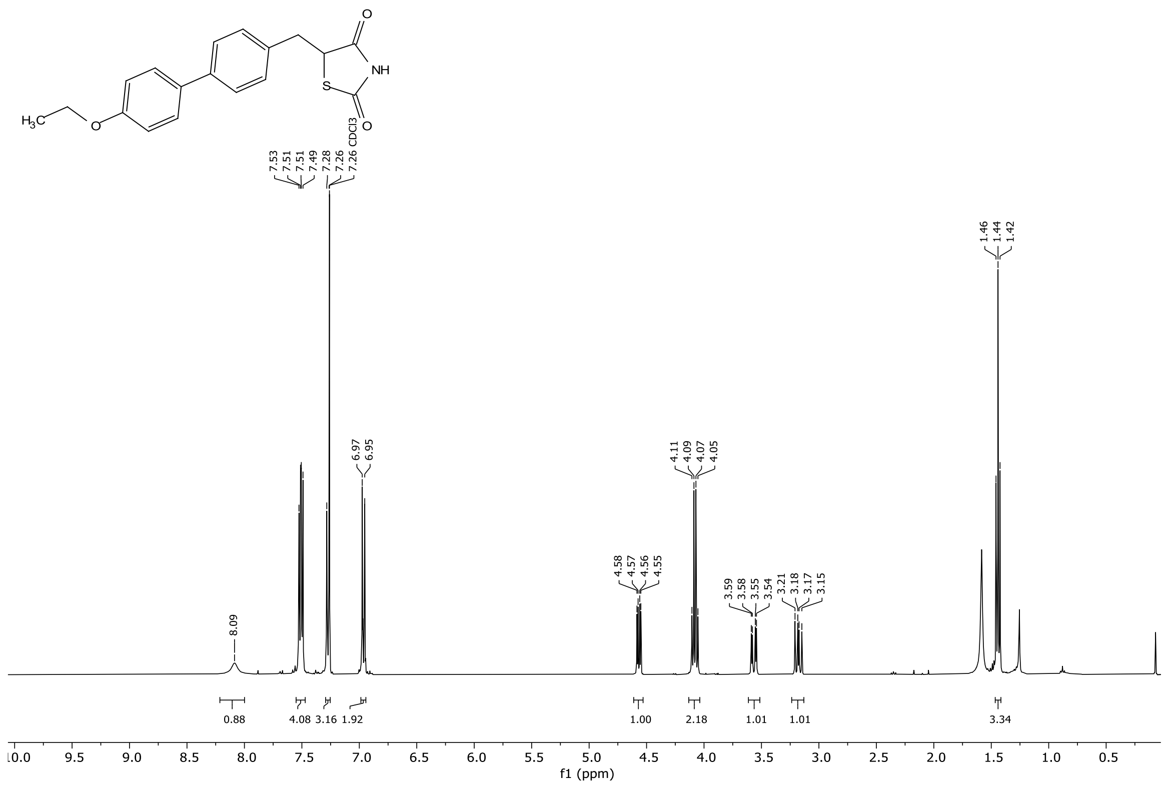

6, **^{13}C{1H} NMR** (101 MHz, $CDCl_3$)

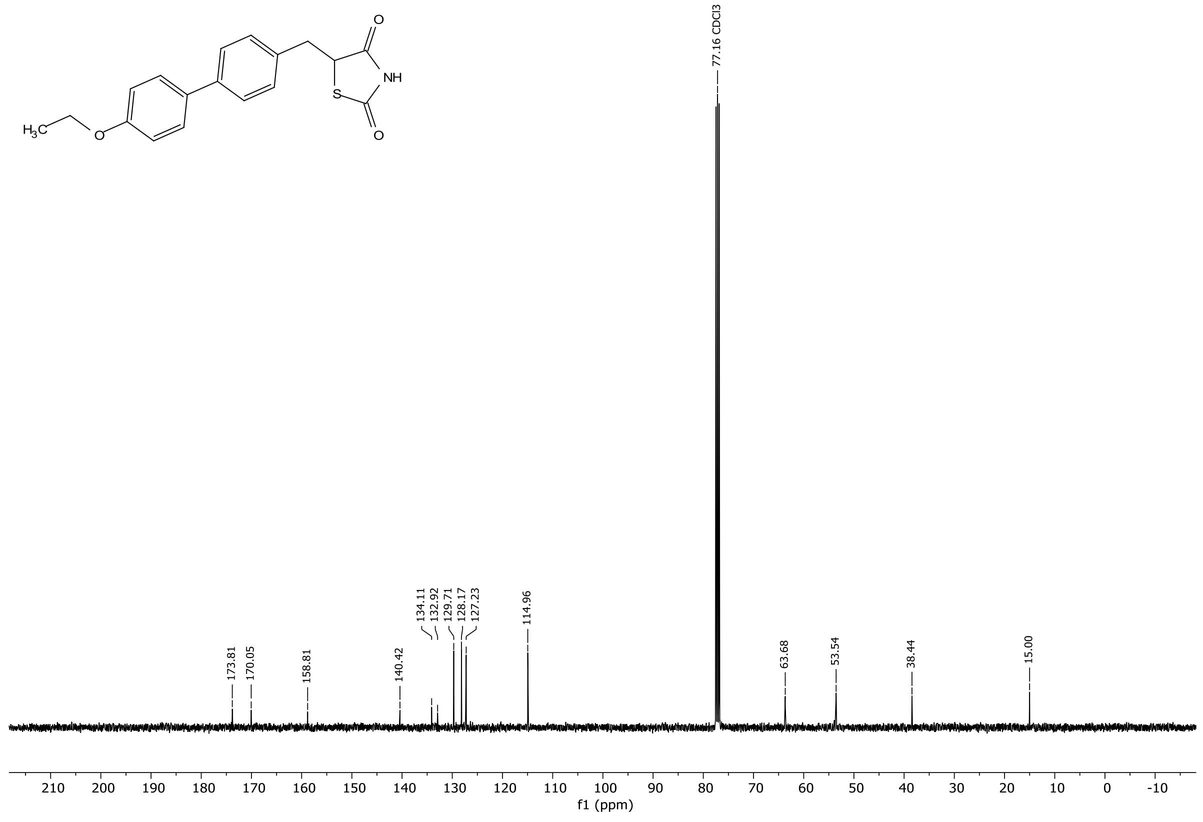

7, ^{1}H NMR (400 MHz, $CDCl_3$)

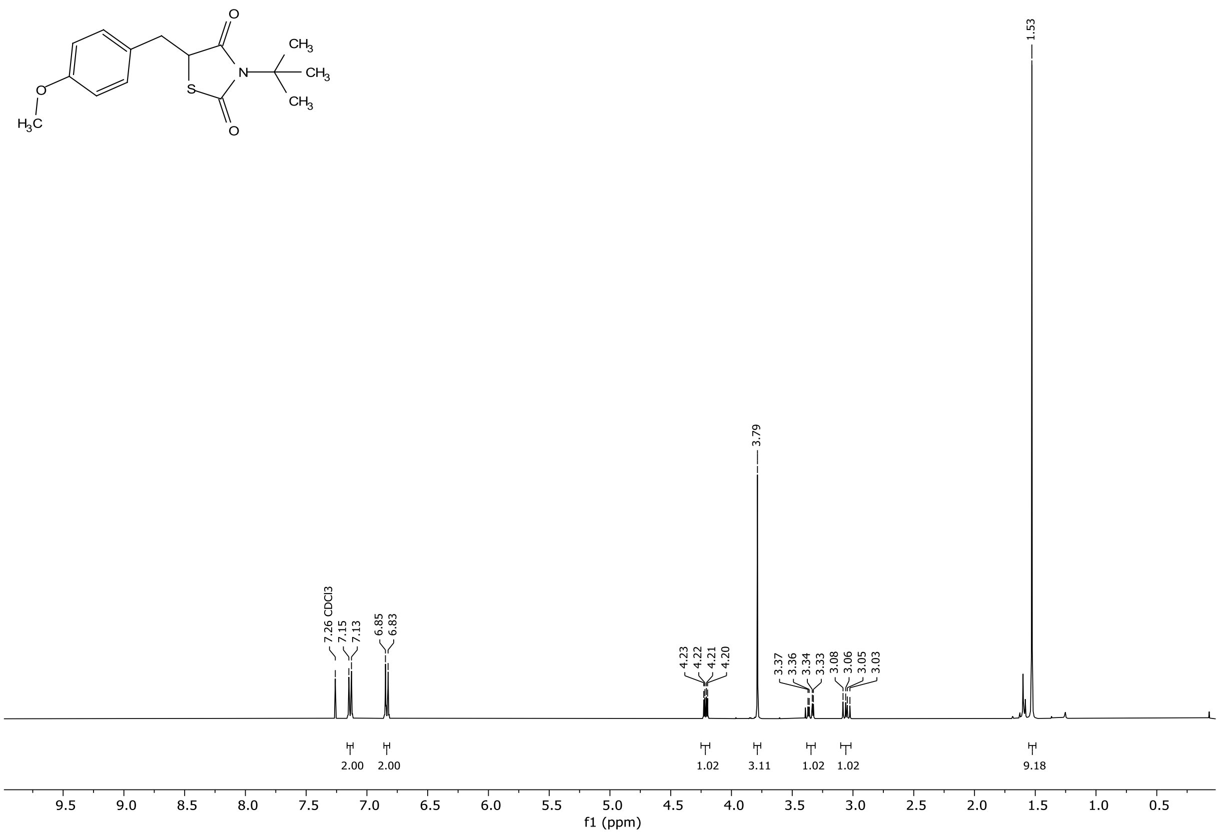

7, **^{13}C{1H} NMR** (101 MHz, $CDCl_3$)

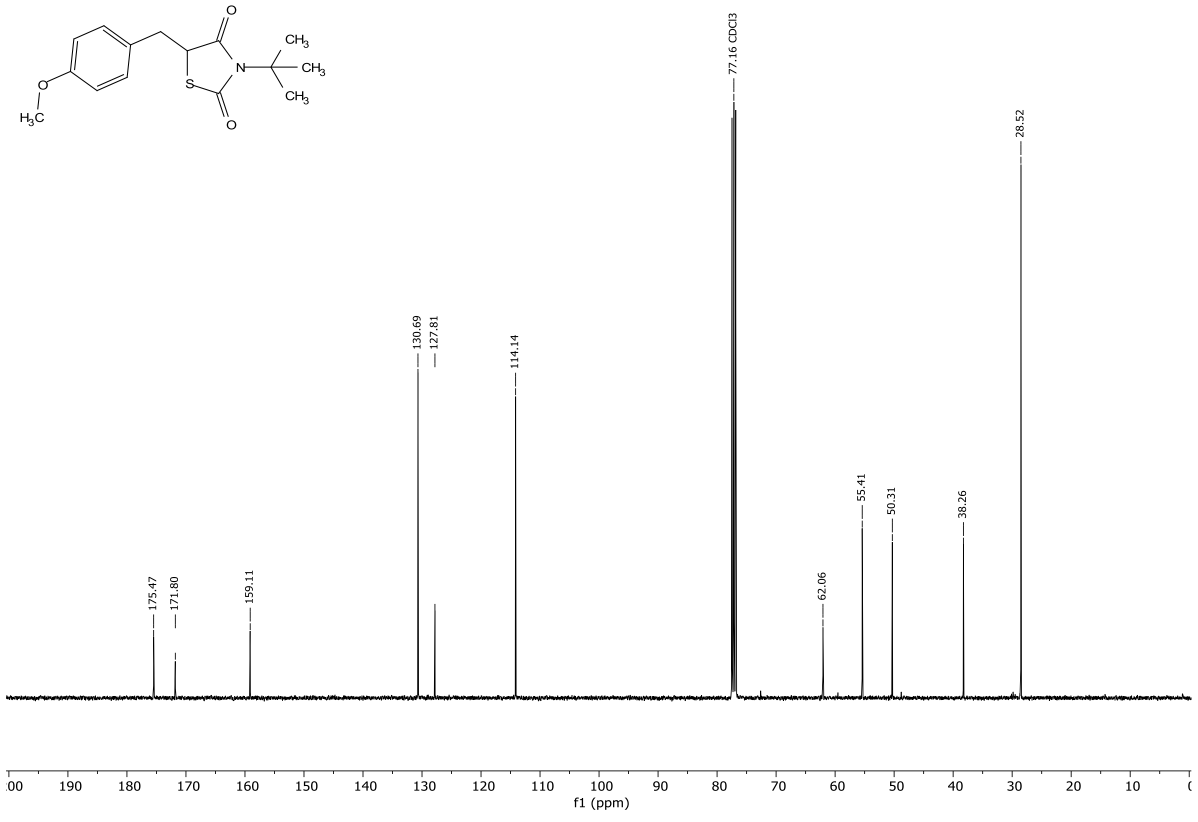

8, ^{1}H NMR (400 MHz, $CDCl_3$)

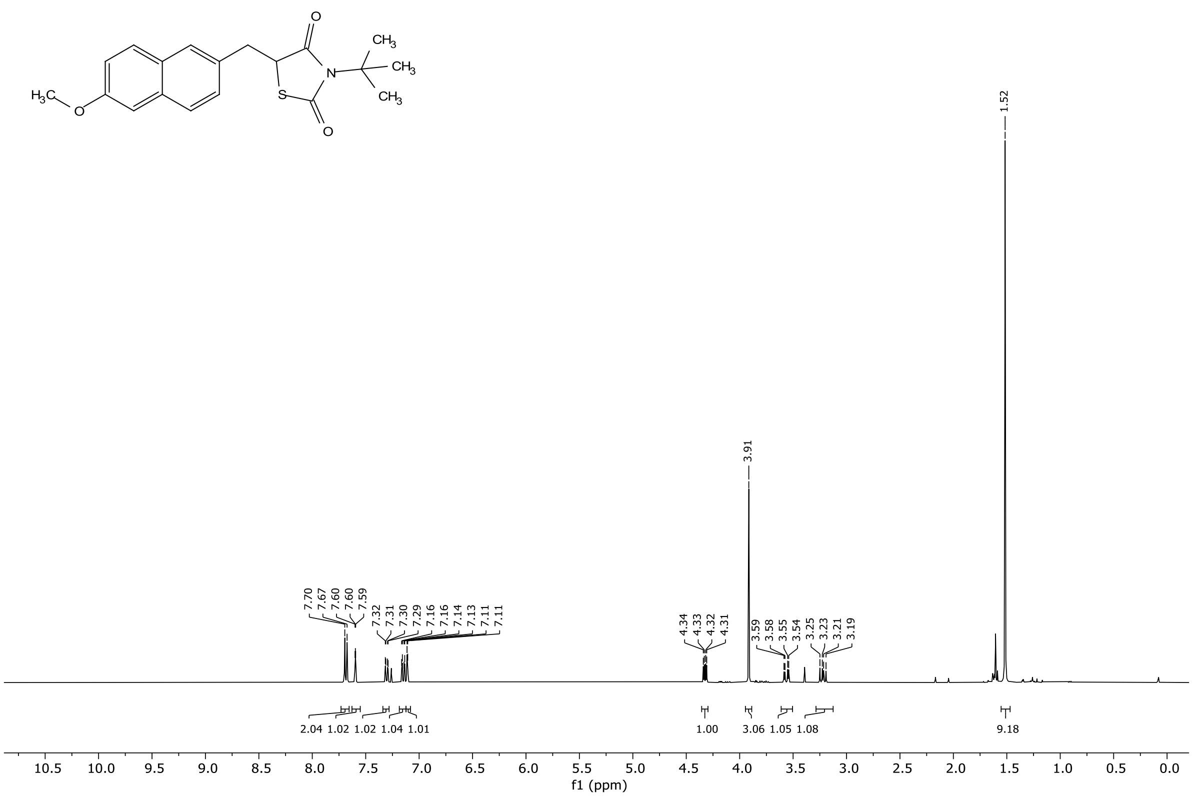

8, **^{13}C{1H} NMR** (101 MHz, $CDCl_3$)

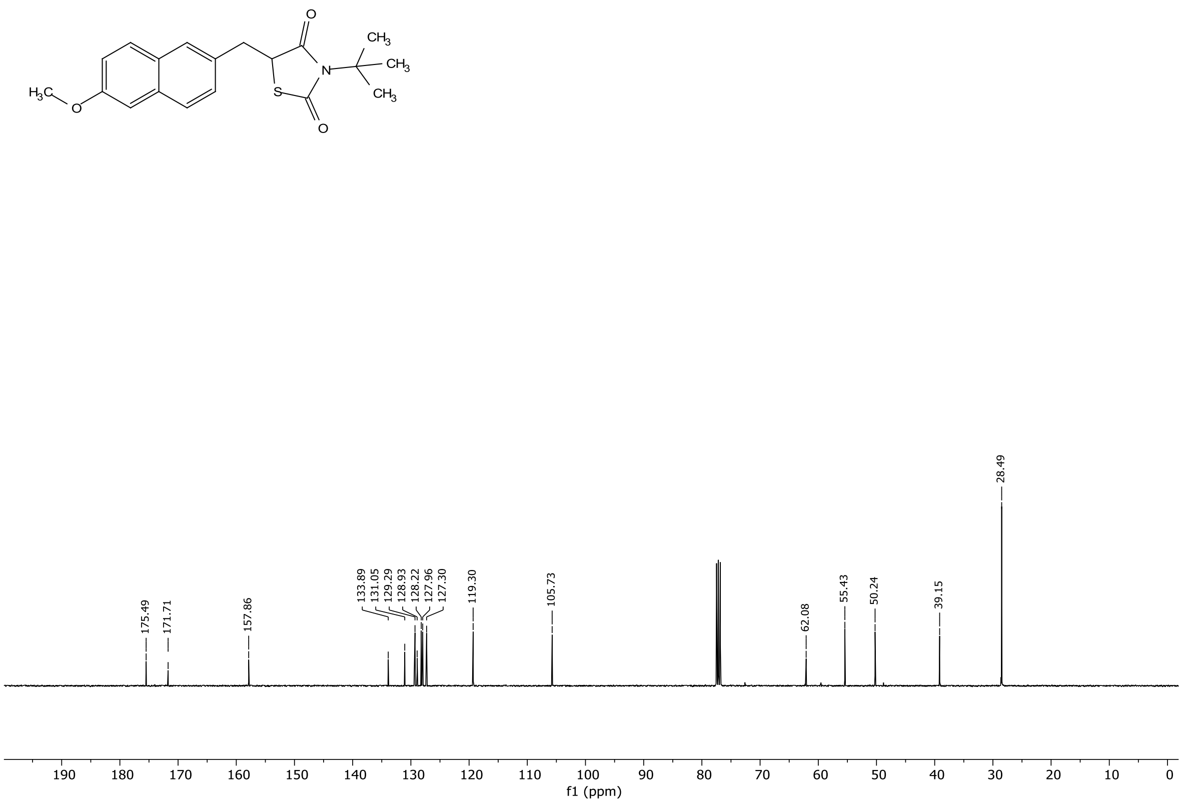

9, ^{1}H NMR (400 MHz, $CDCl_3$)

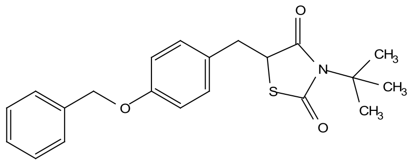

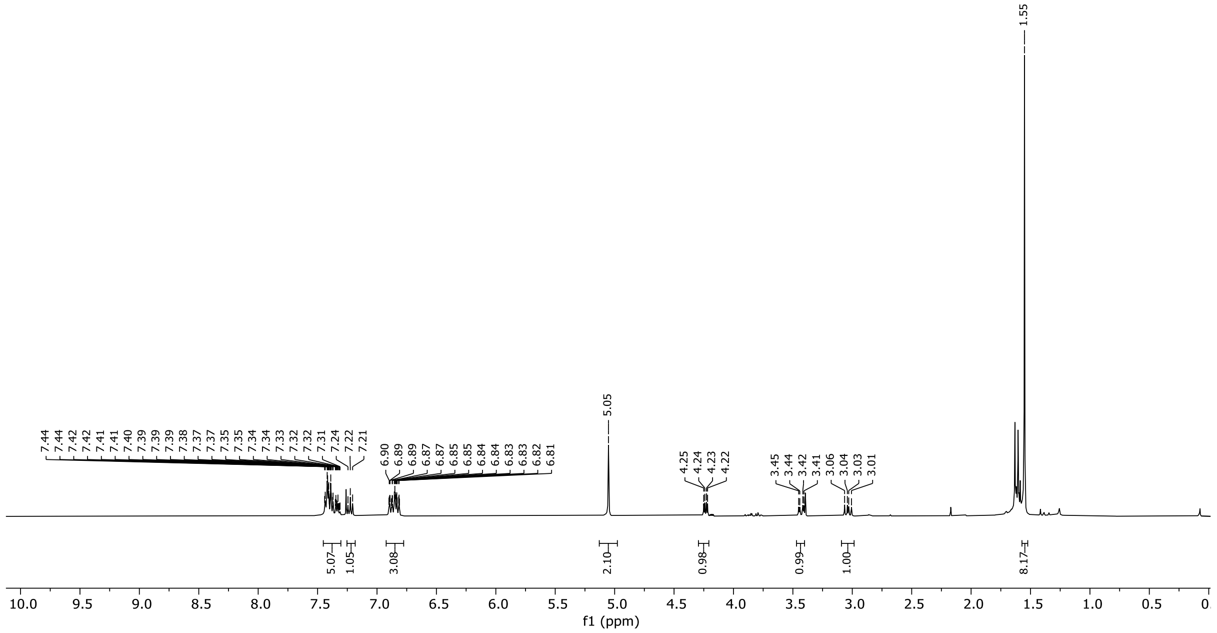

9, **^{13}C{1H} NMR** (101 MHz, $CDCl_3$)

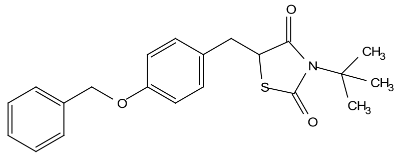

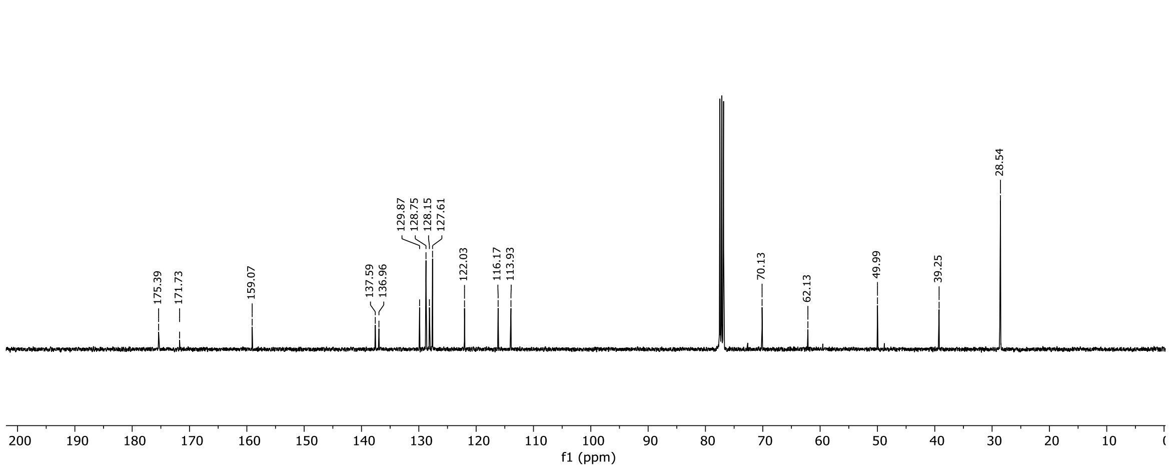

10, ^{1}H NMR (400 MHz, $CDCl_3$)

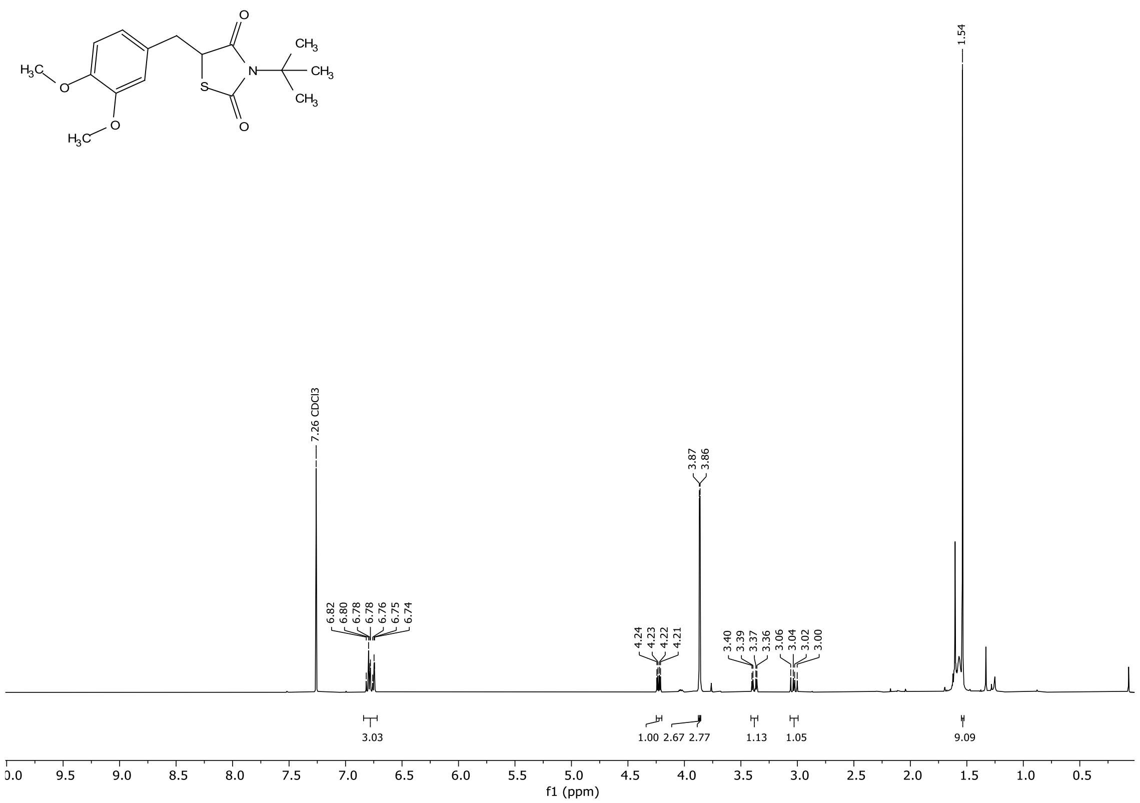

10, ^{13}**C{1H} NMR** (101 MHz, $CDCl_3$)

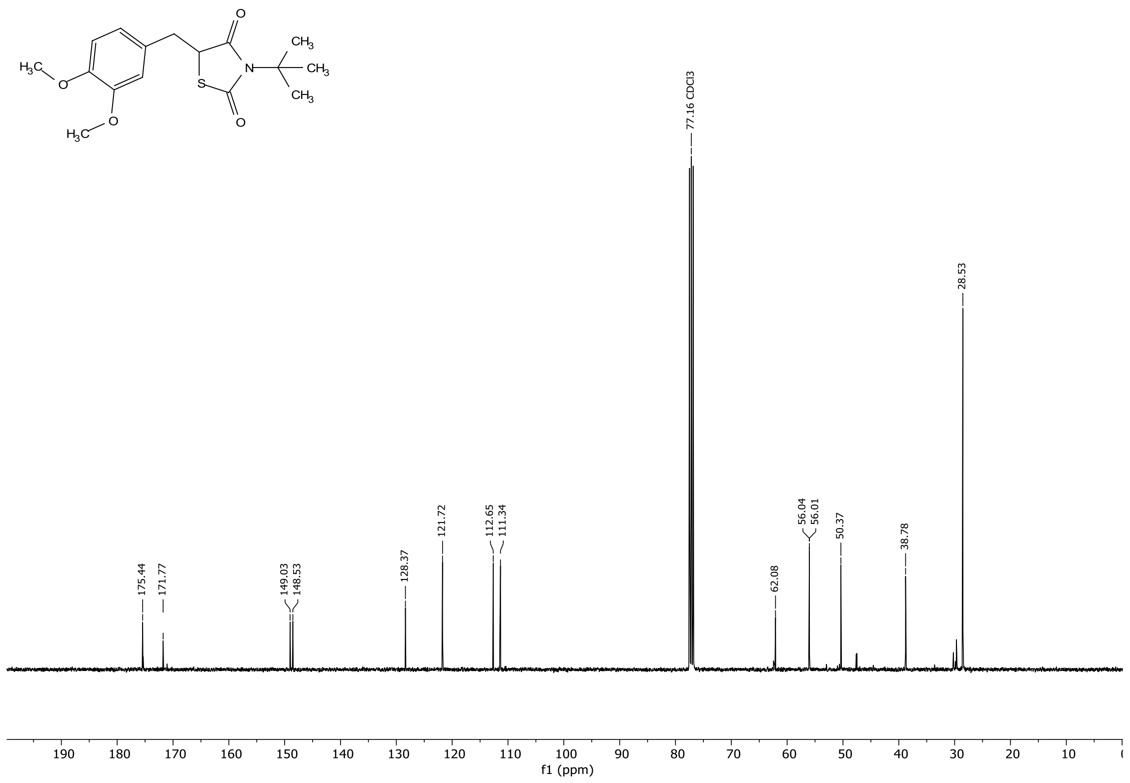

11, ^{1}H NMR (400 MHz, CDCl$_3$)

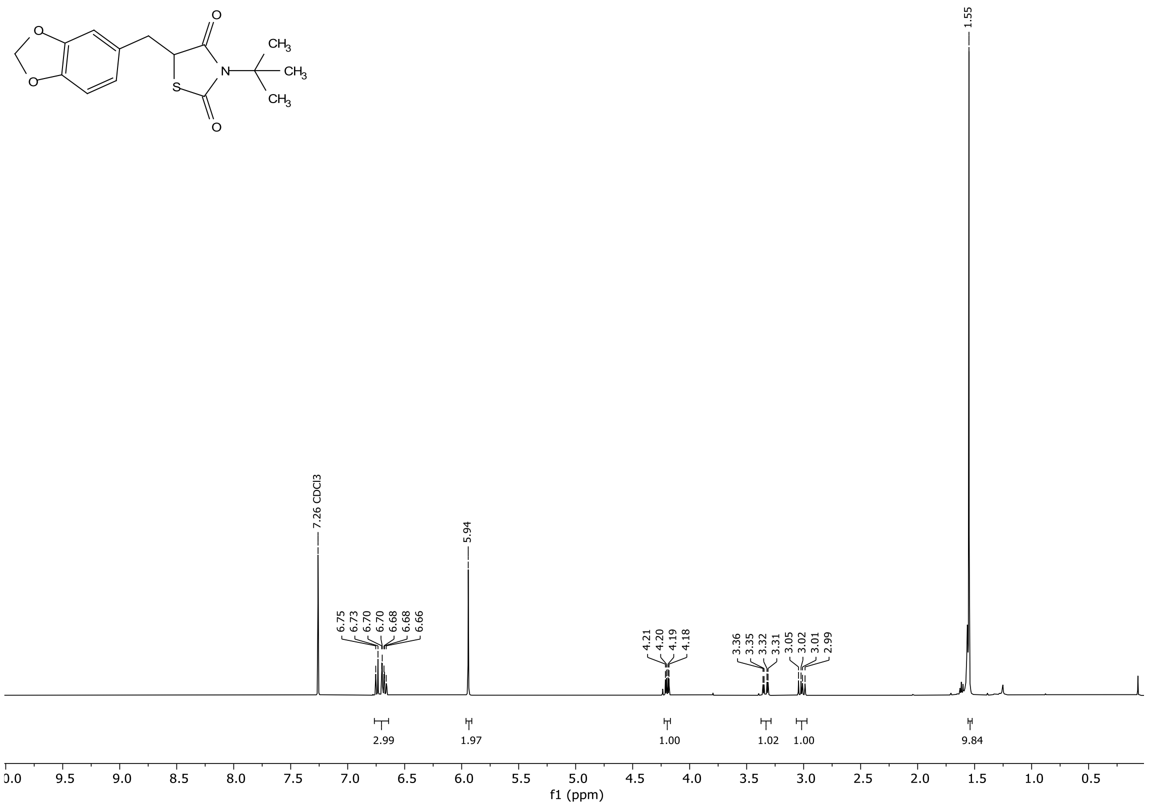

11, **^{13}C{1H} NMR** (101 MHz, CDCl$_3$)

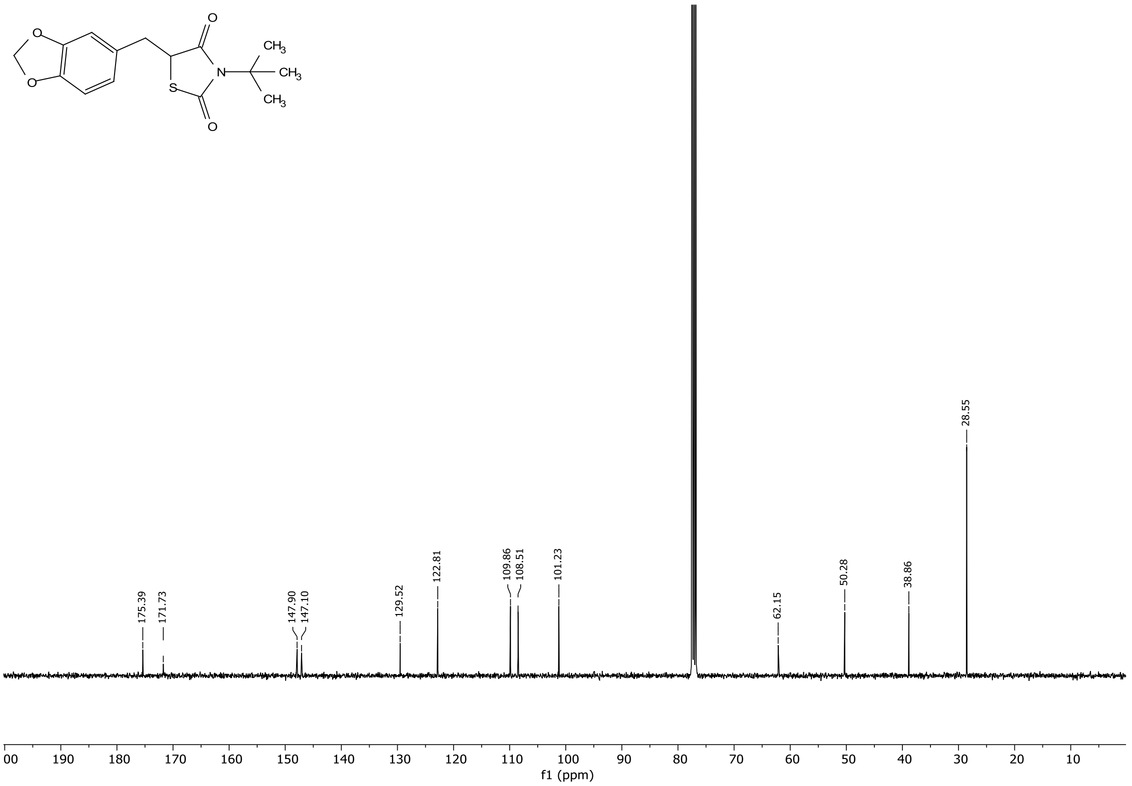

12, ^{1}H NMR (400 MHz, $CDCl_3$)

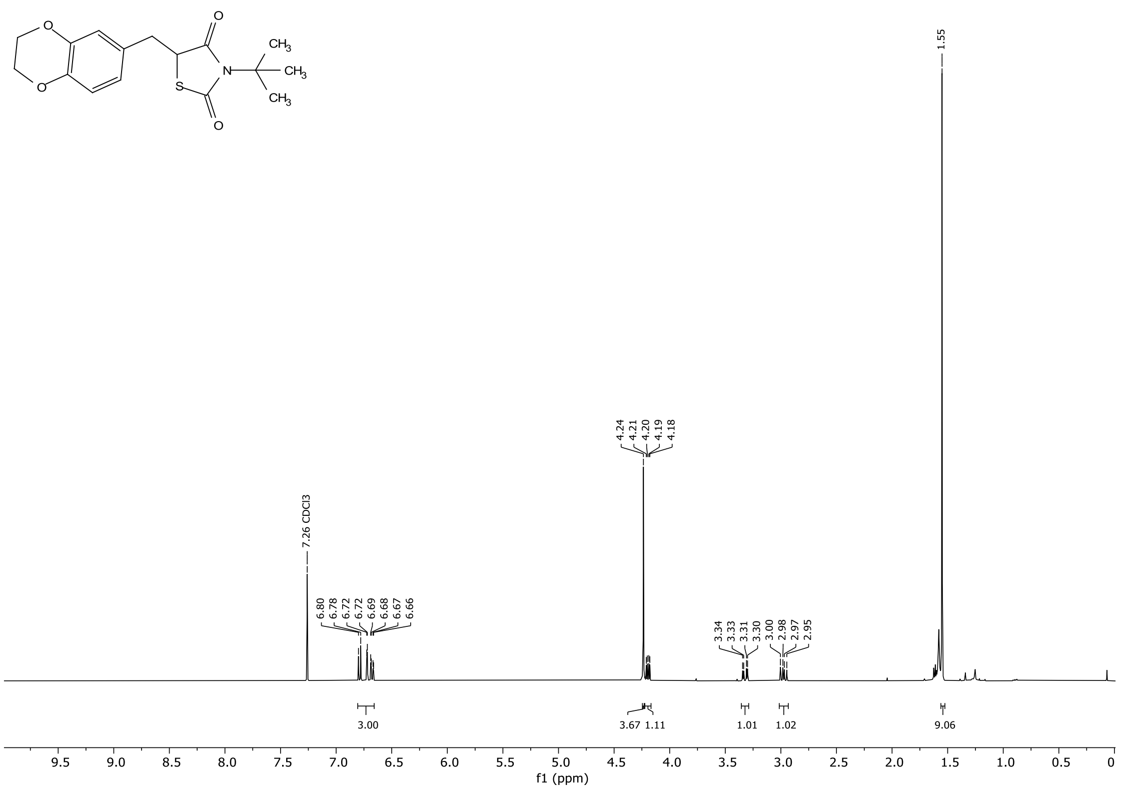

12, **^{13}C{1H} NMR** (101 MHz, $CDCl_3$)

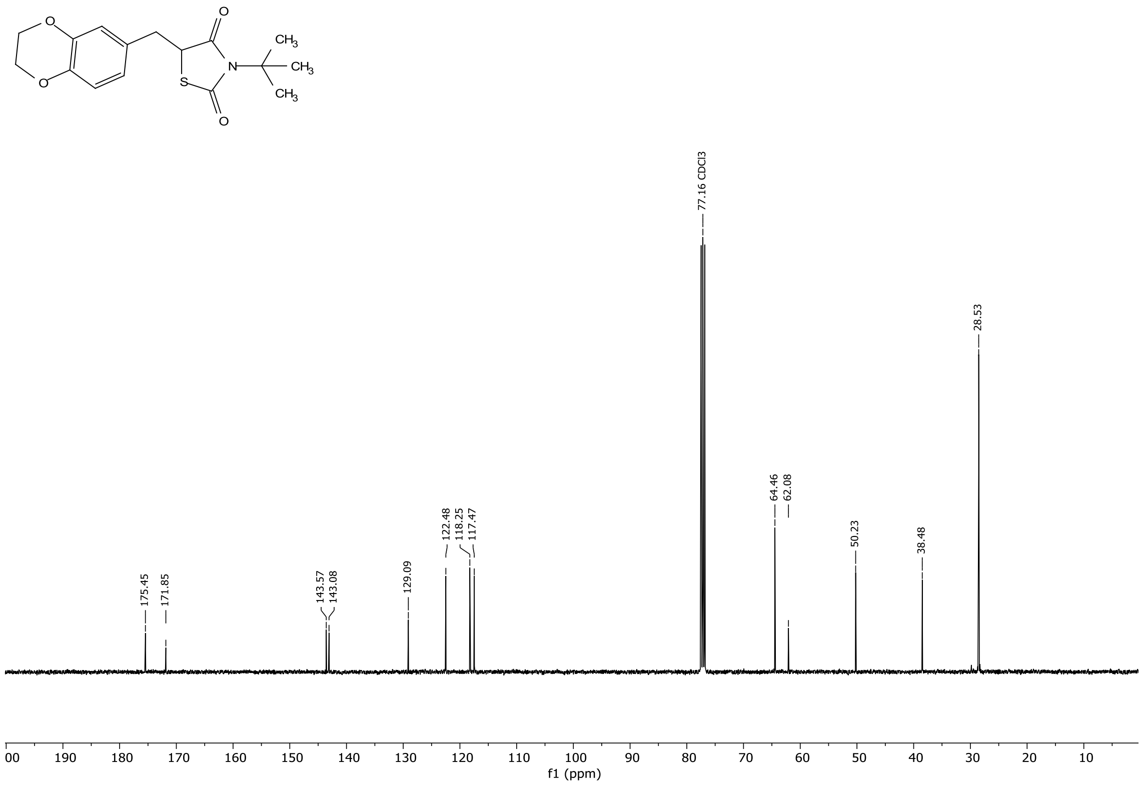

13, ^{1}H NMR (400 MHz, $CDCl_3$)

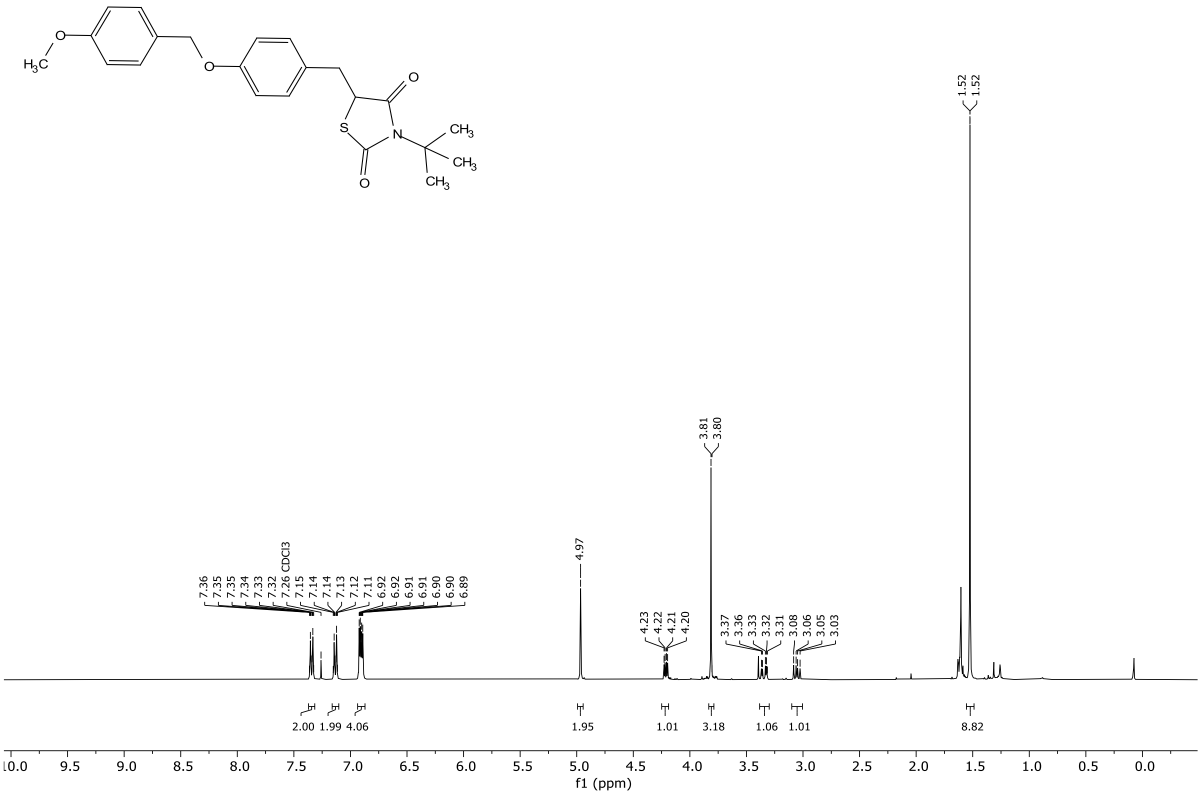

13, **^{13}C{1H} NMR** (101 MHz, $CDCl_3$)

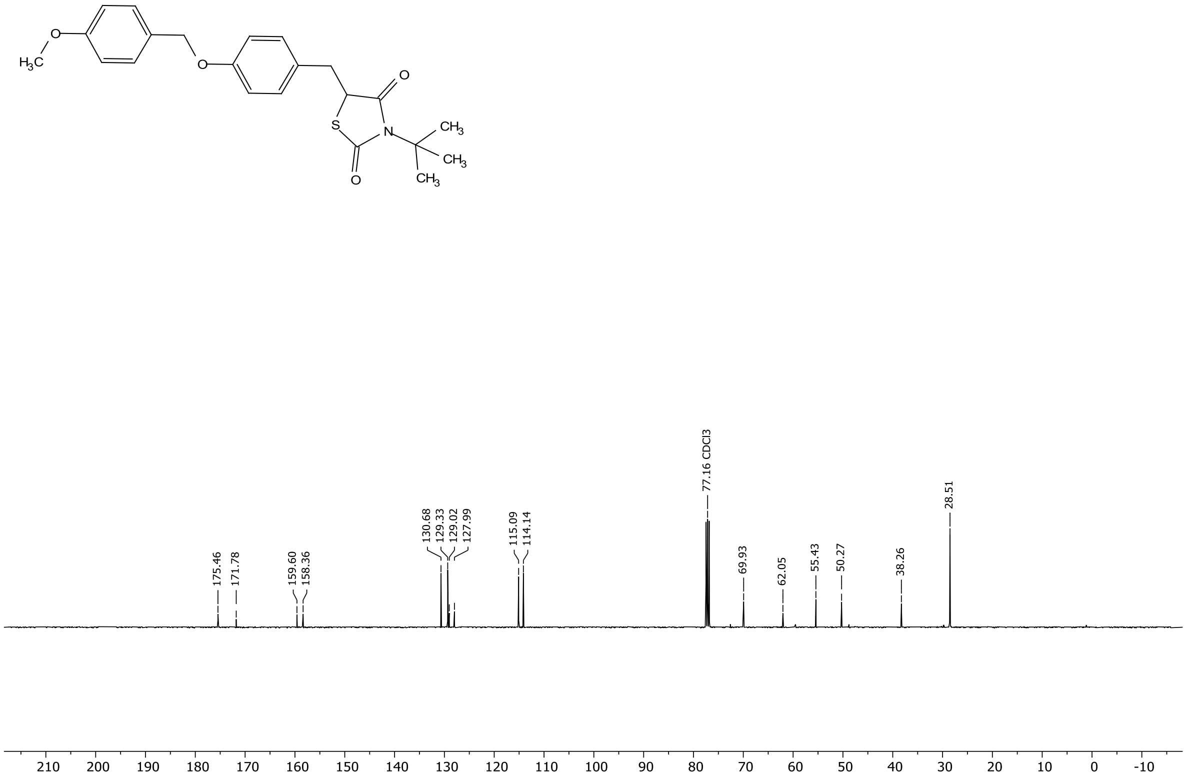

14, ^{1}H NMR (400 MHz, $CDCl_3$)

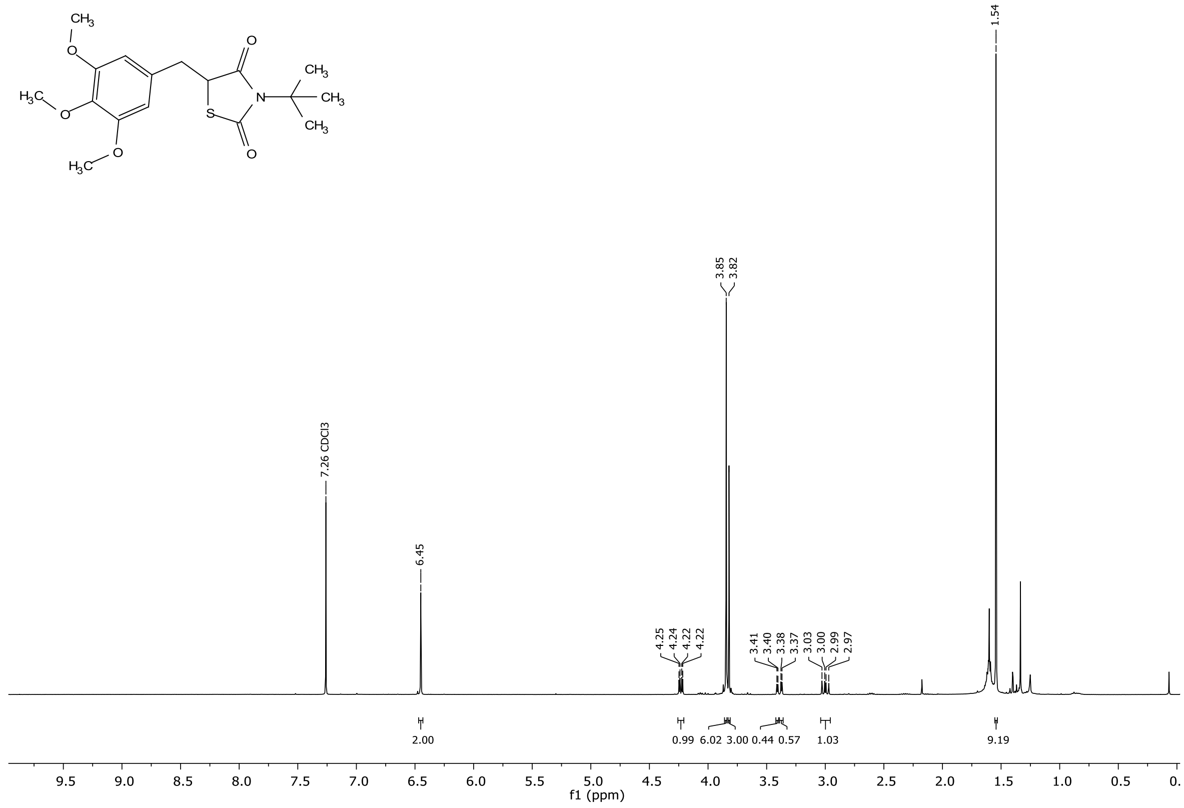

14, **^{13}C{1H} NMR** (101 MHz, $CDCl_3$)

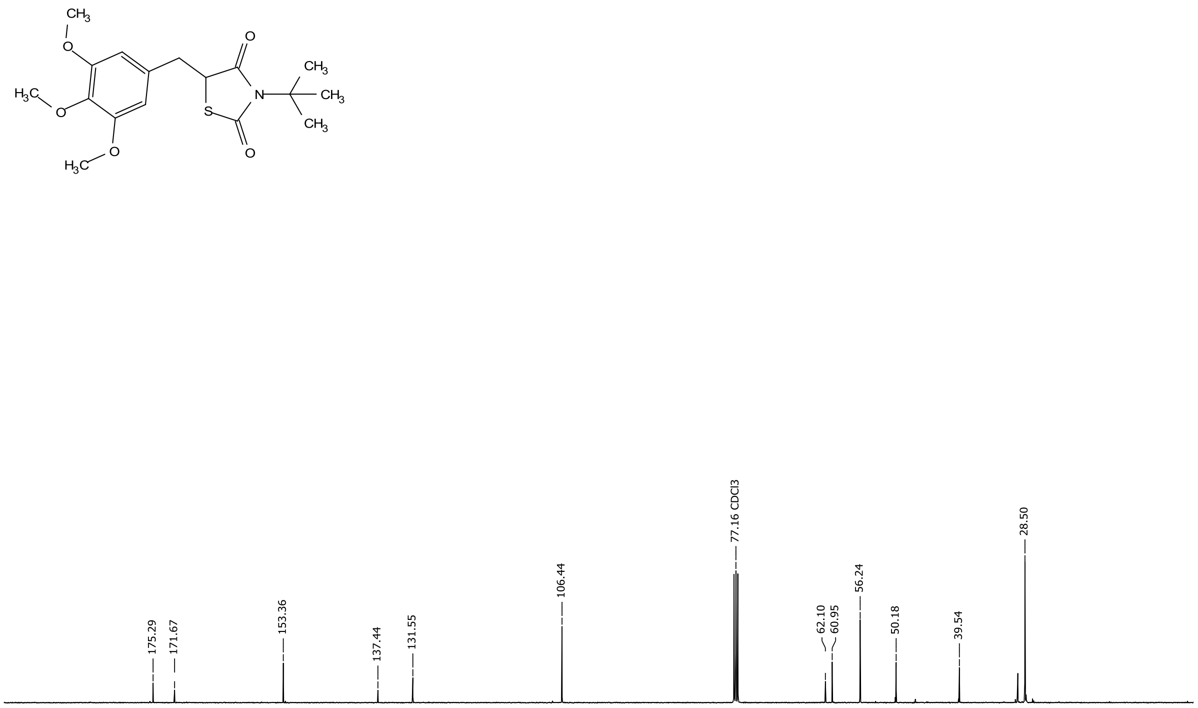

15, ^{1}H NMR (400 MHz, $CDCl_3$)

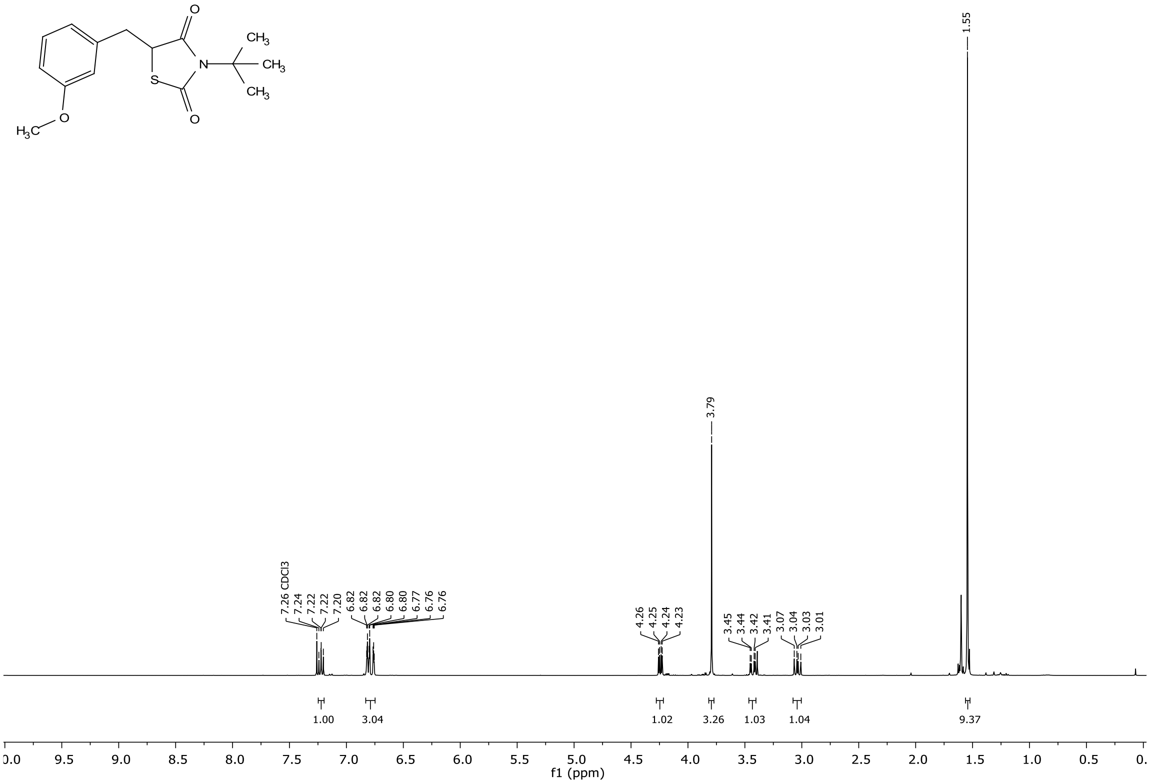

15, **^{13}C{1H} NMR** (101 MHz, $CDCl_3$)

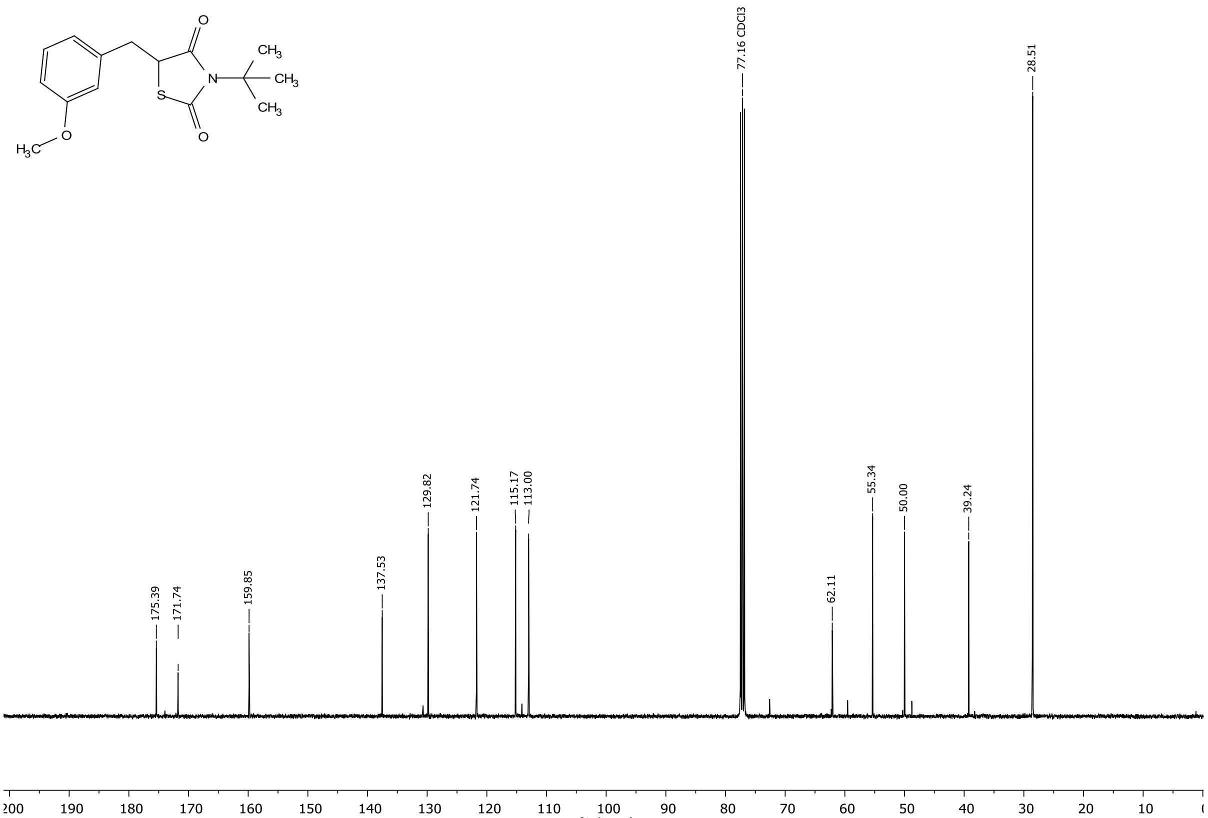

16, ^{1}H NMR (400 MHz, $CDCl_3$)

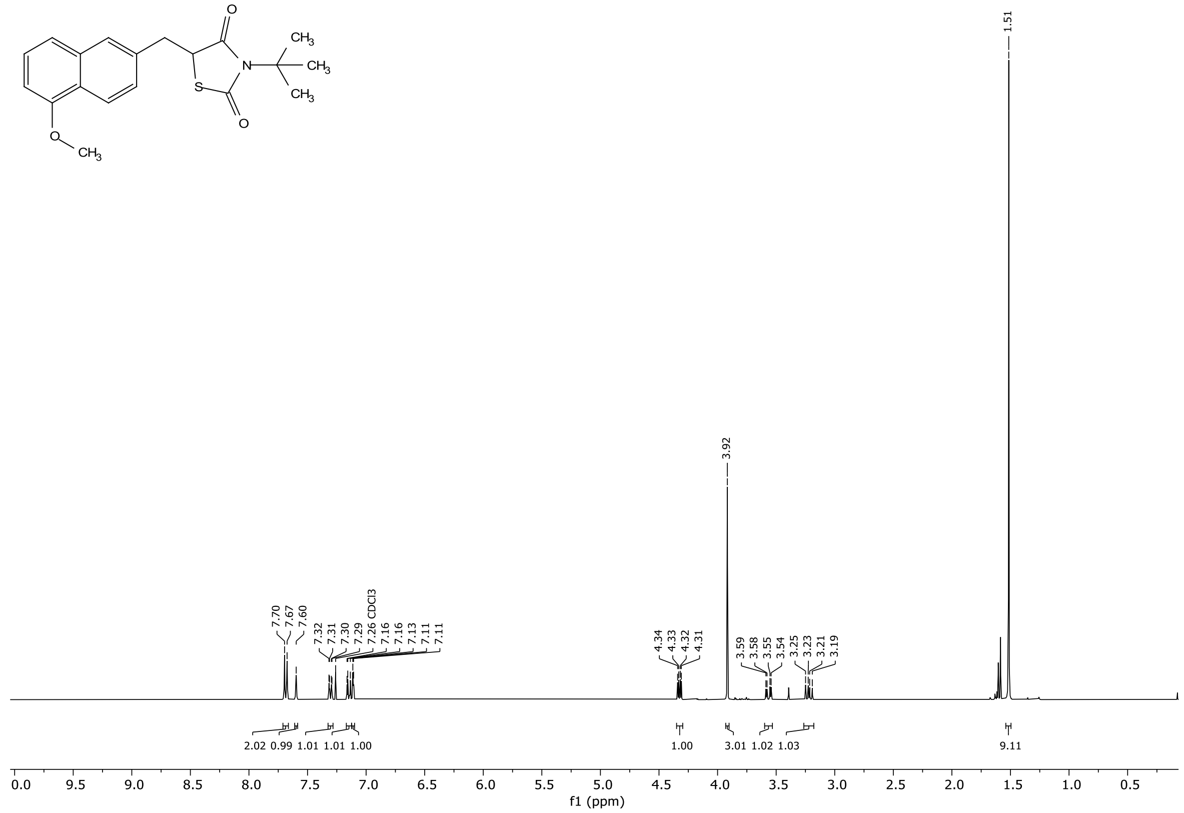

16, **^{13}C{1H} NMR** (101 MHz, $CDCl_3$)

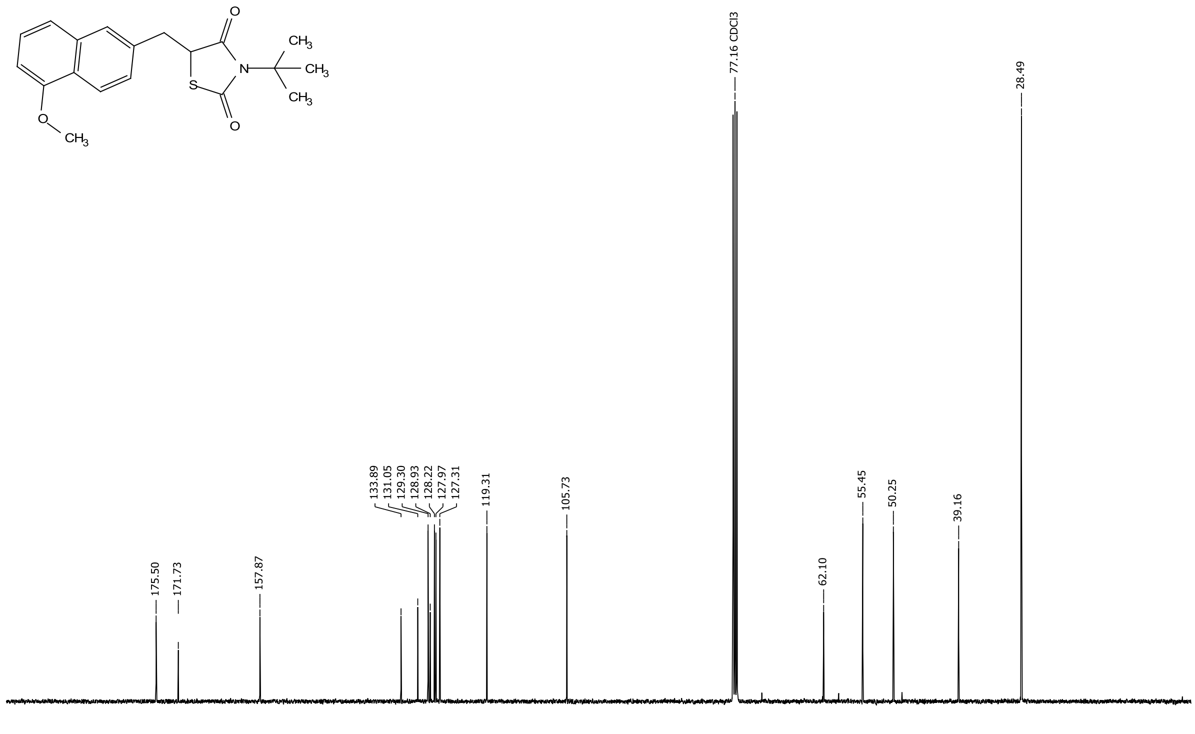

17, ^{1}H NMR (400 MHz, $CDCl_3$)

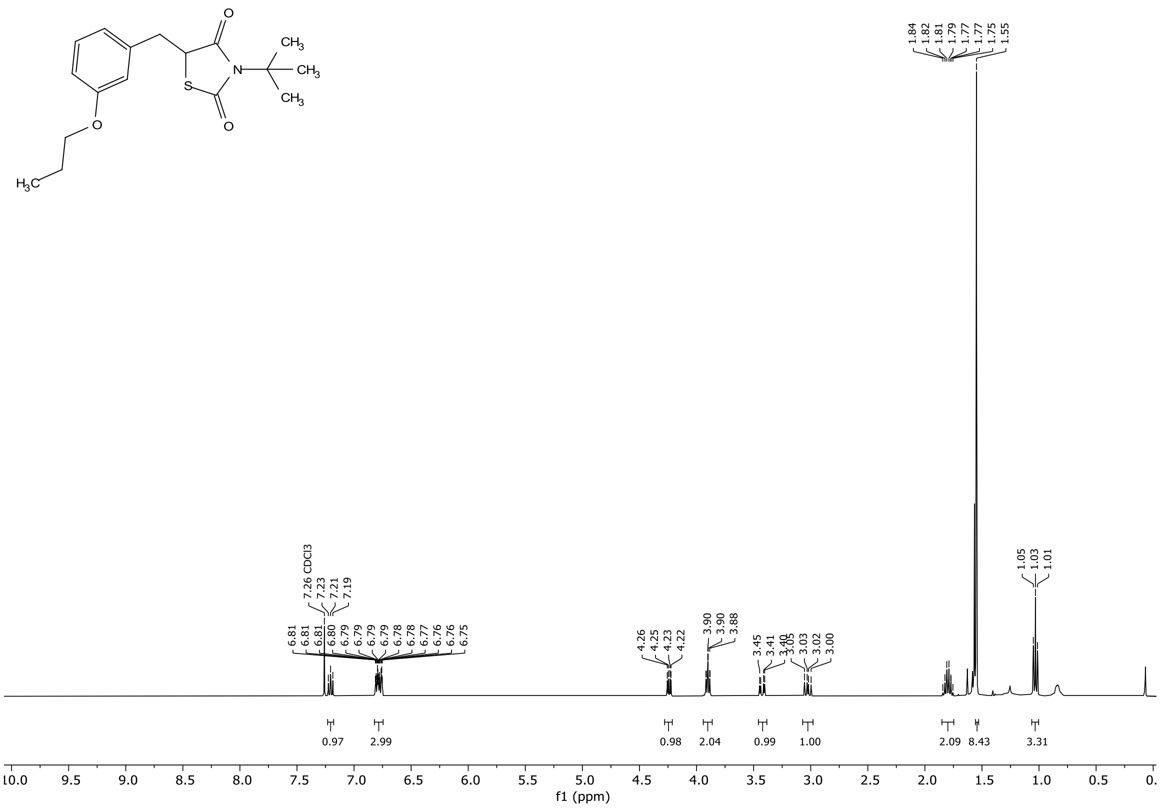

17, ^{13}C{1H} NMR (101 MHz, $CDCl_3$)

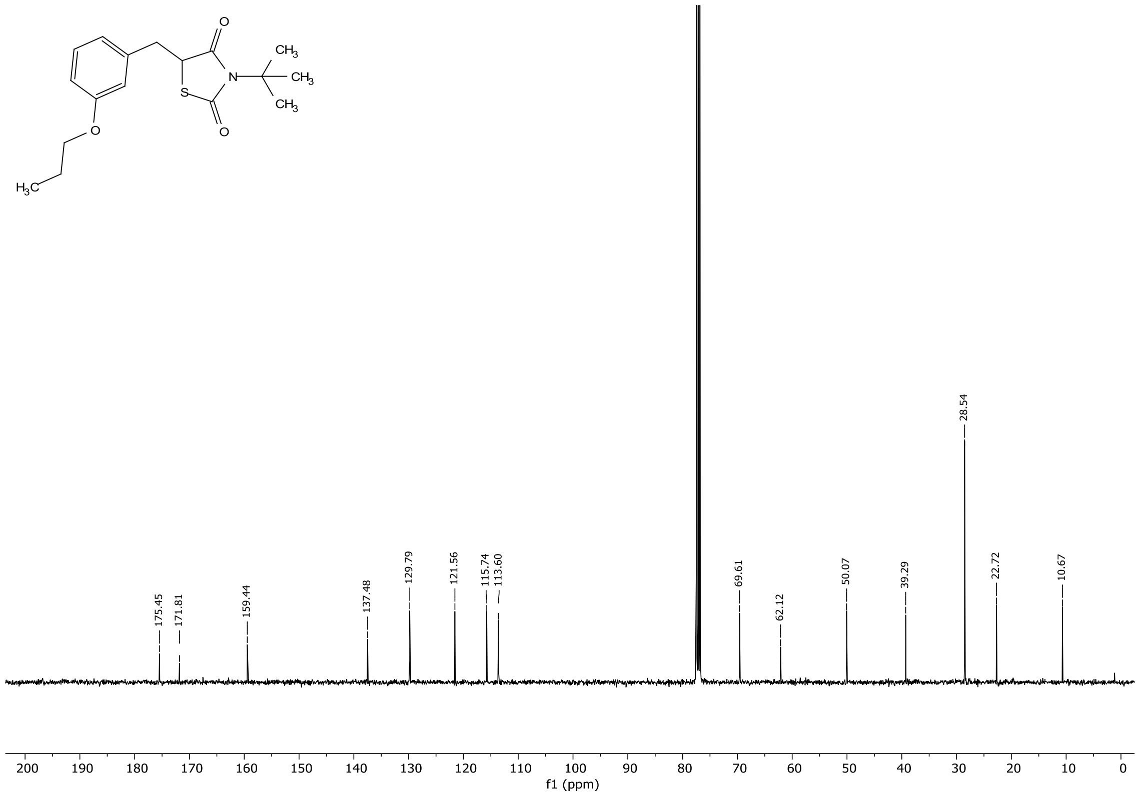

18, ^{1}H NMR (400 MHz, $CDCl_3$)

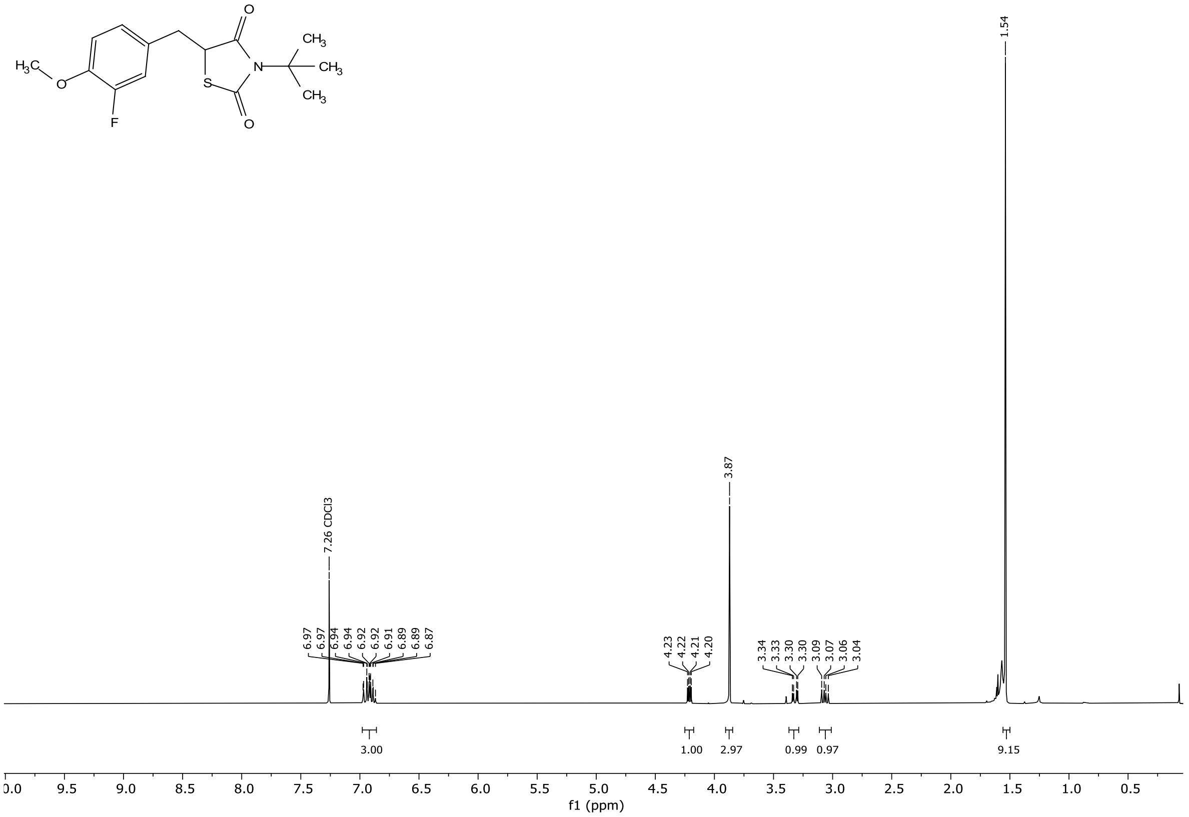

18, **^{13}C{1H} NMR** (101 MHz, $CDCl_3$)

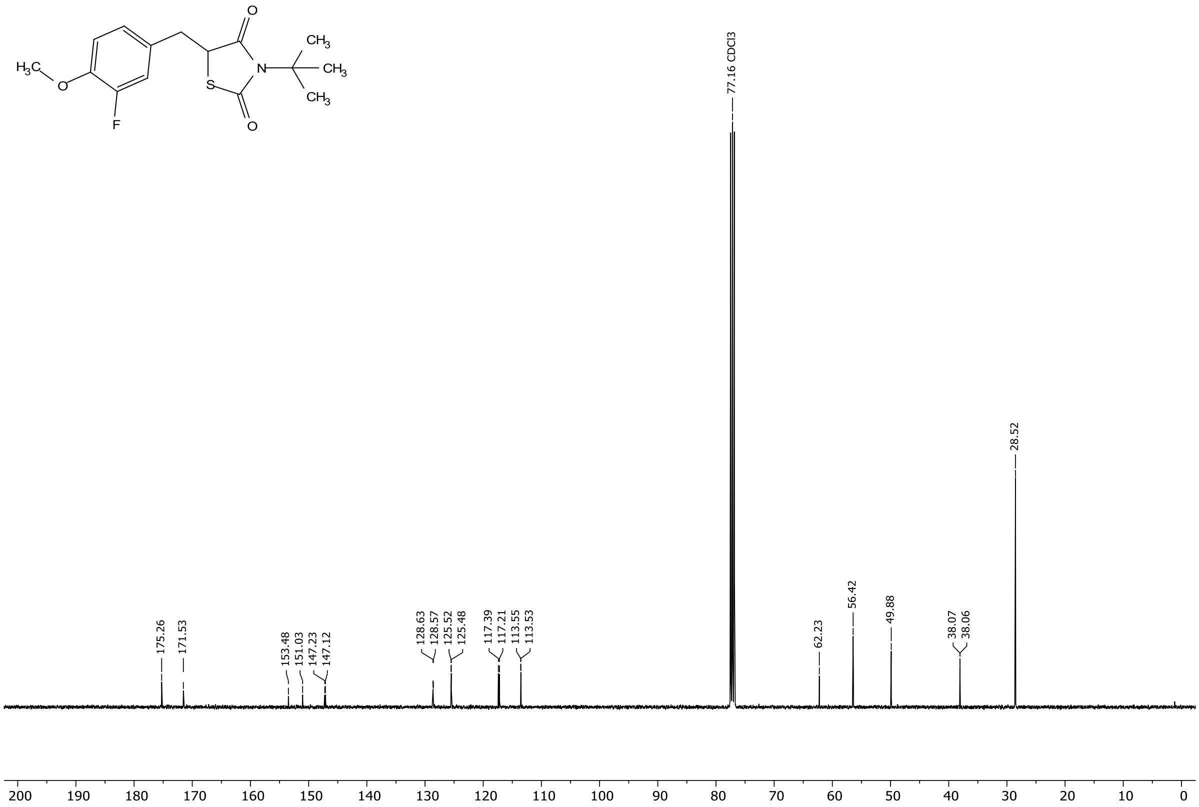

19, ^{1}H NMR (400 MHz, $CDCl_3$)

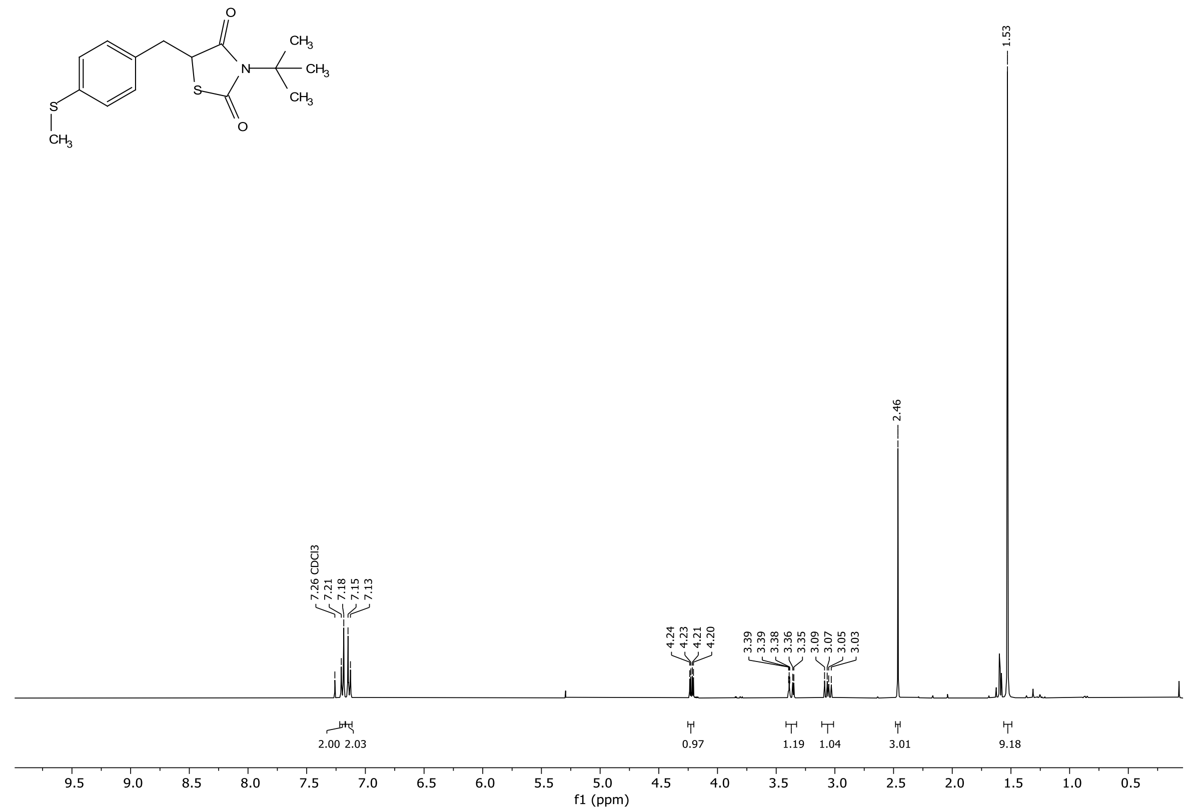

19, **^{13}C{1H} NMR** (101 MHz, $CDCl_3$)

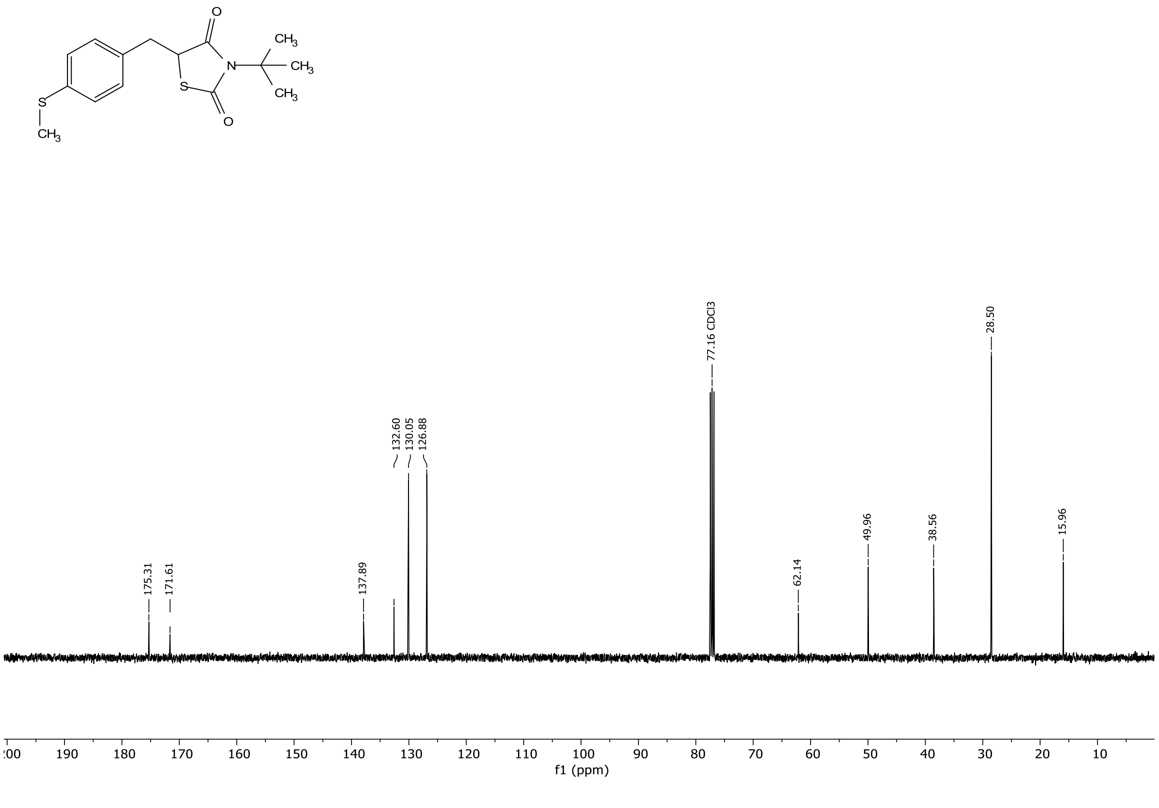

20, ^{1}H NMR (400 MHz, $CDCl_3$)

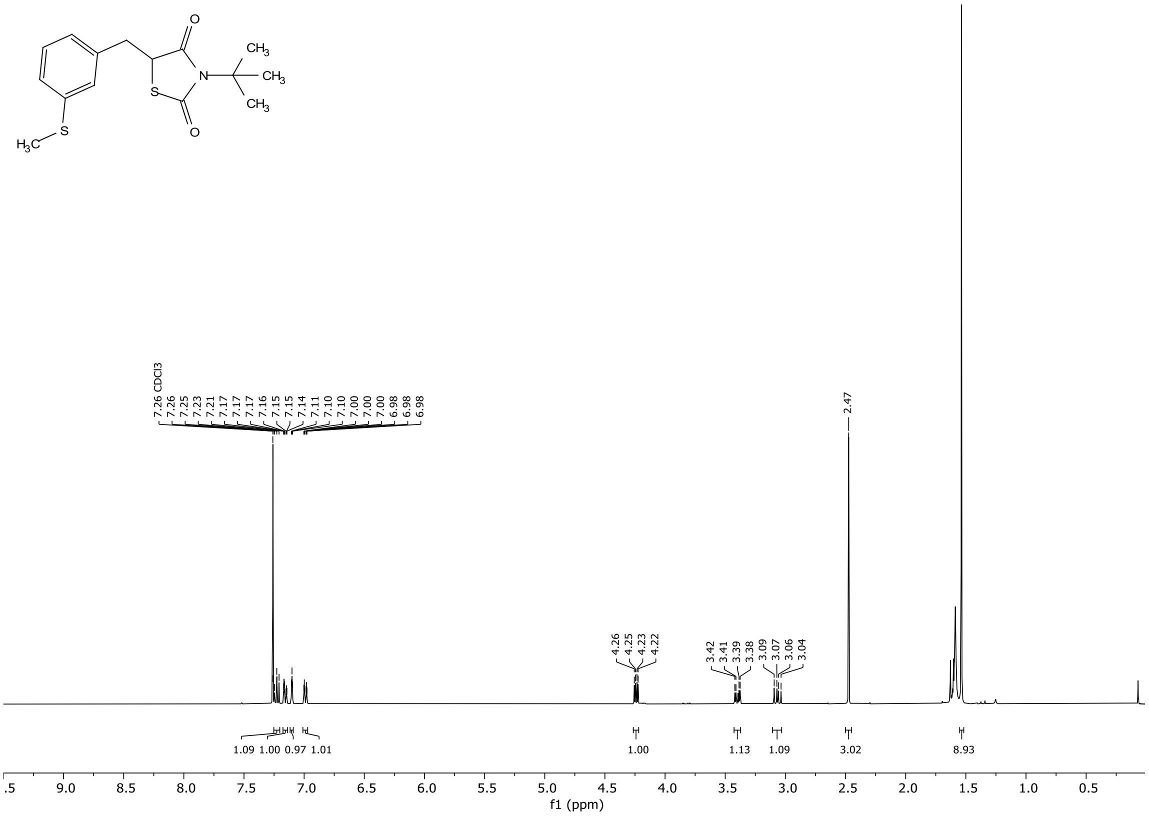

20, **^{13}C{1H} NMR** (101 MHz, $CDCl_3$)

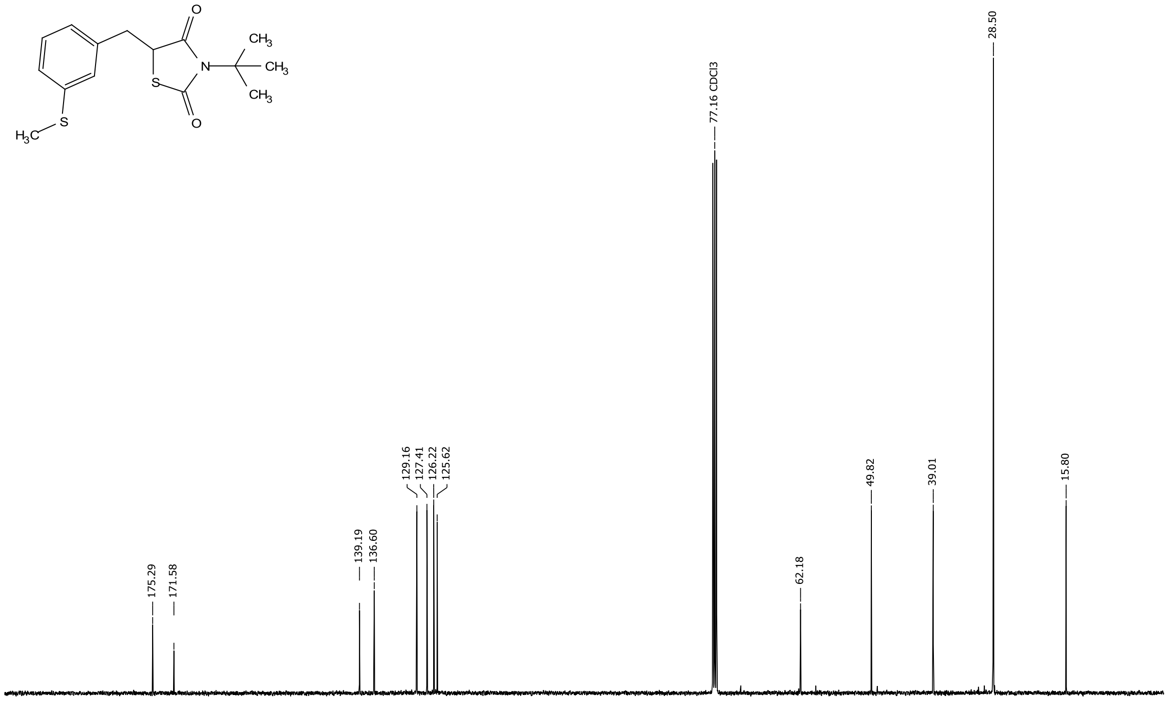

21, ^{1}H NMR (400 MHz, $CDCl_3$)

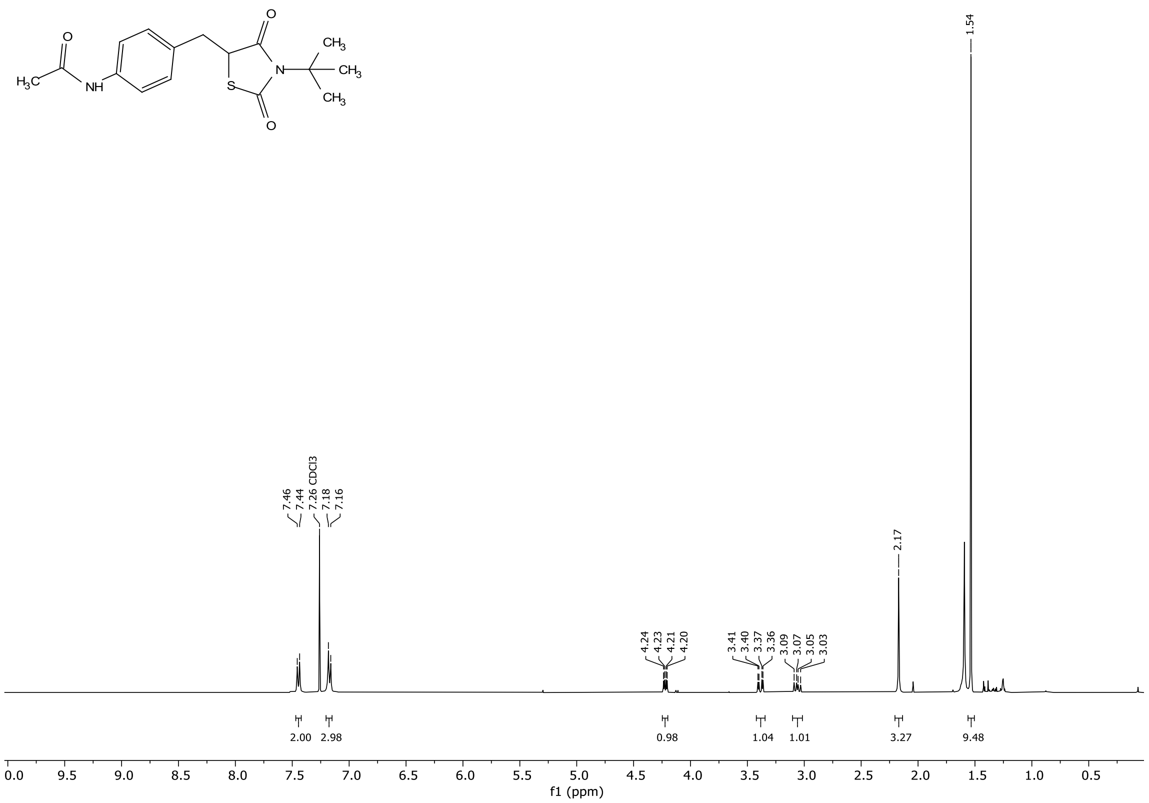

21, **^{13}C{1H} NMR** (101 MHz, $CDCl_3$)

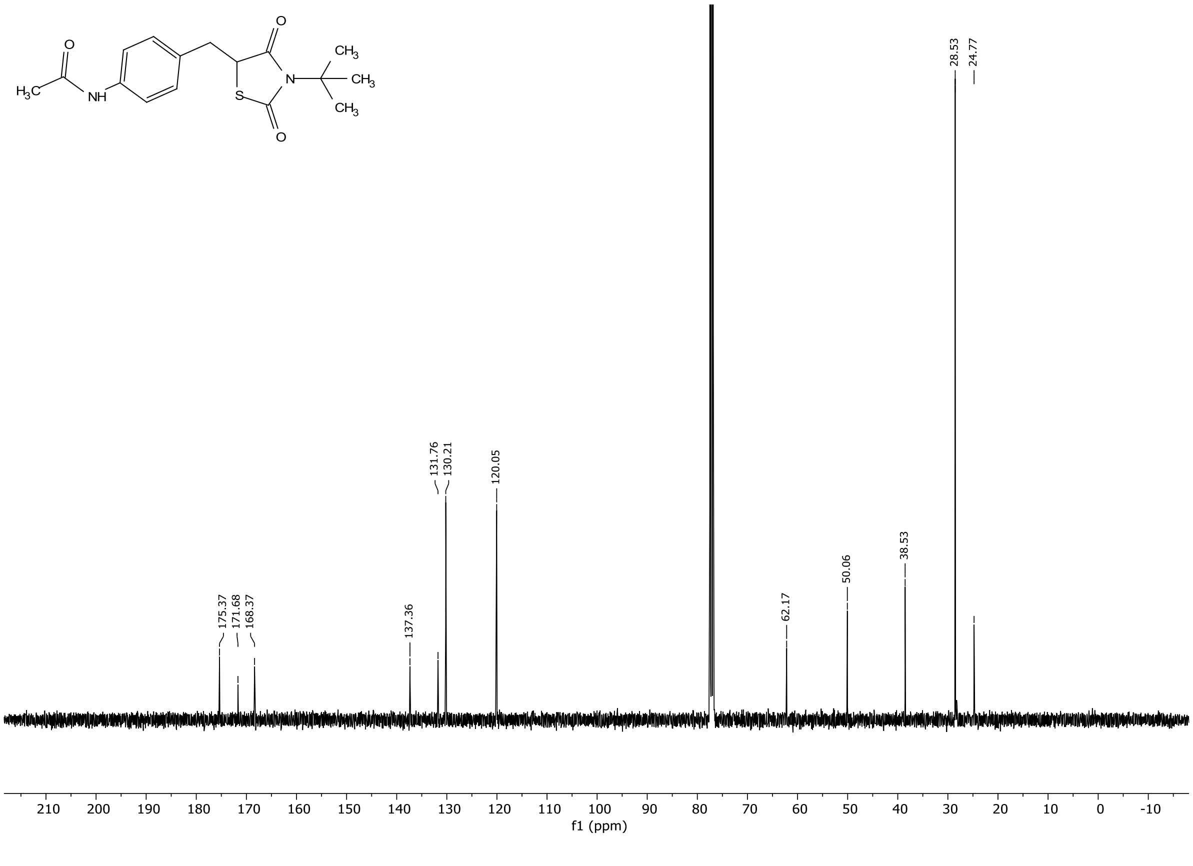

22, ^{1}H NMR (400 MHz, $CDCl_3$)

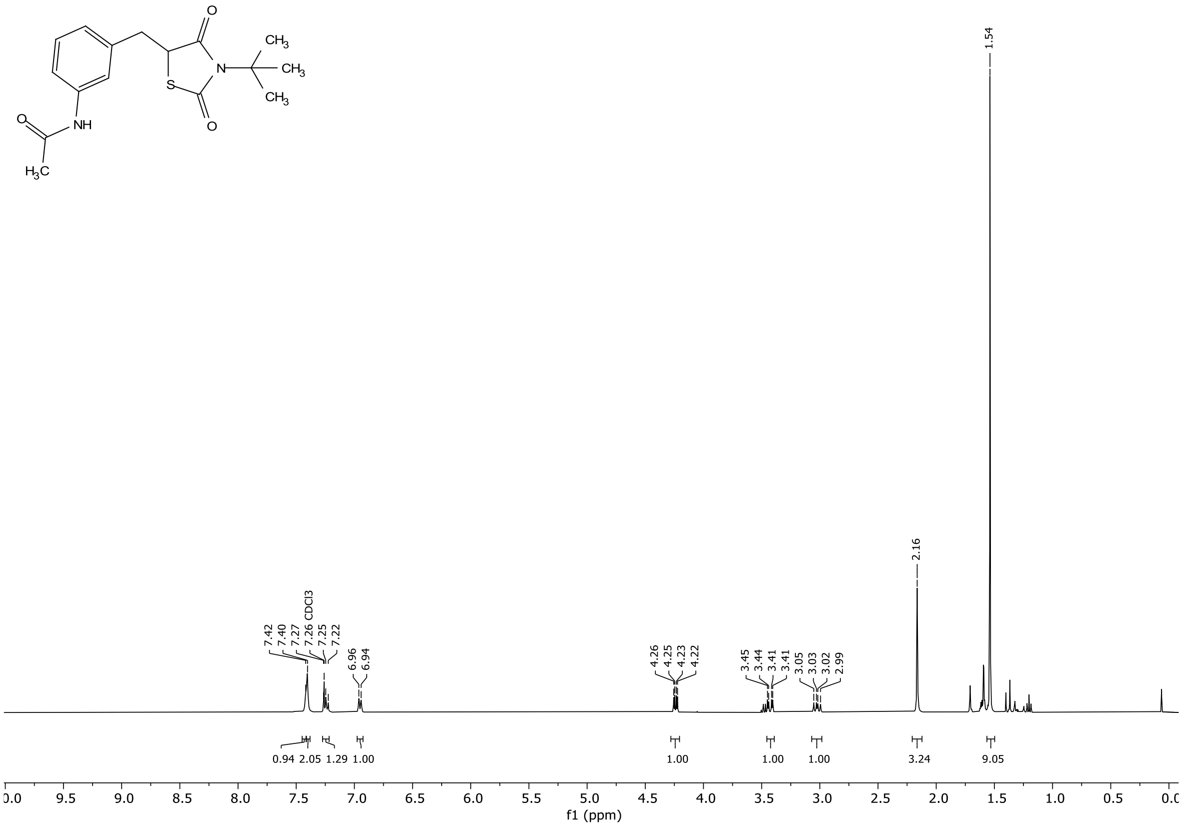

22, **^{13}C{1H} NMR** (101 MHz, $CDCl_3$)

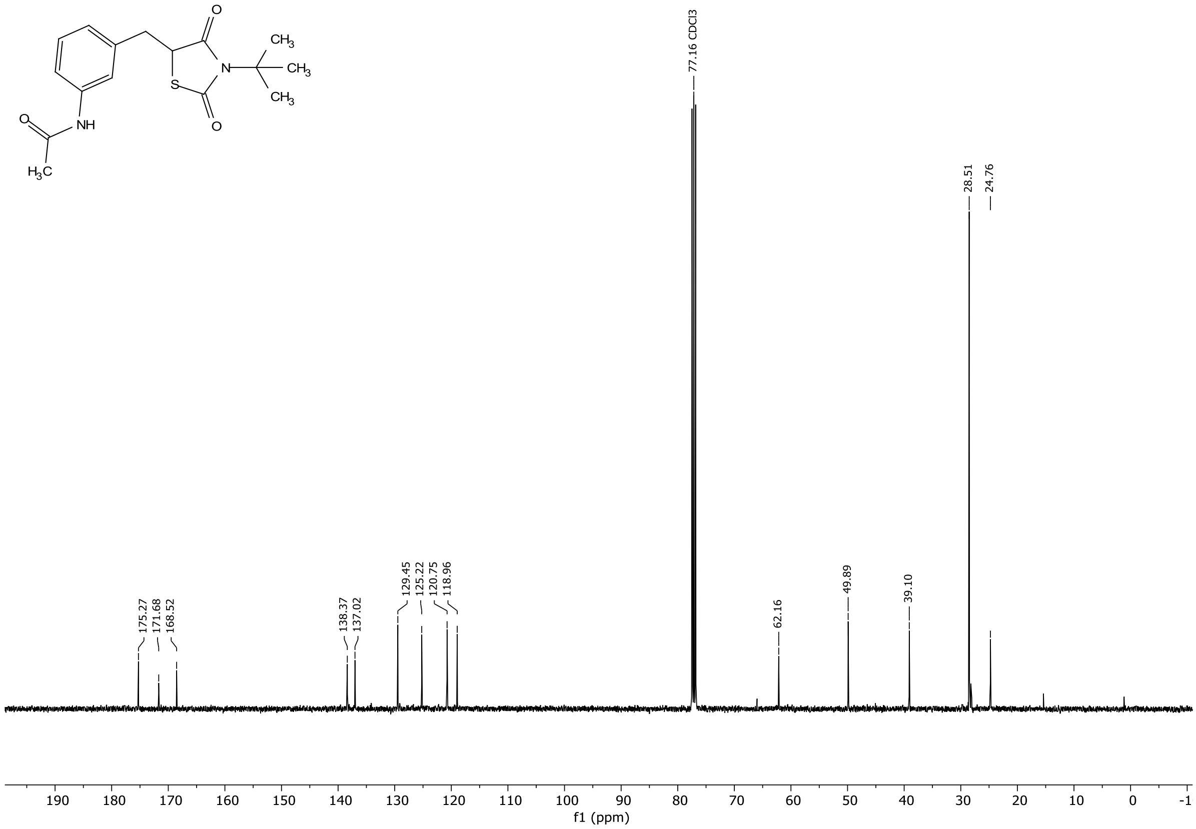

23, ^{1}H NMR (400 MHz, $CDCl_3$)

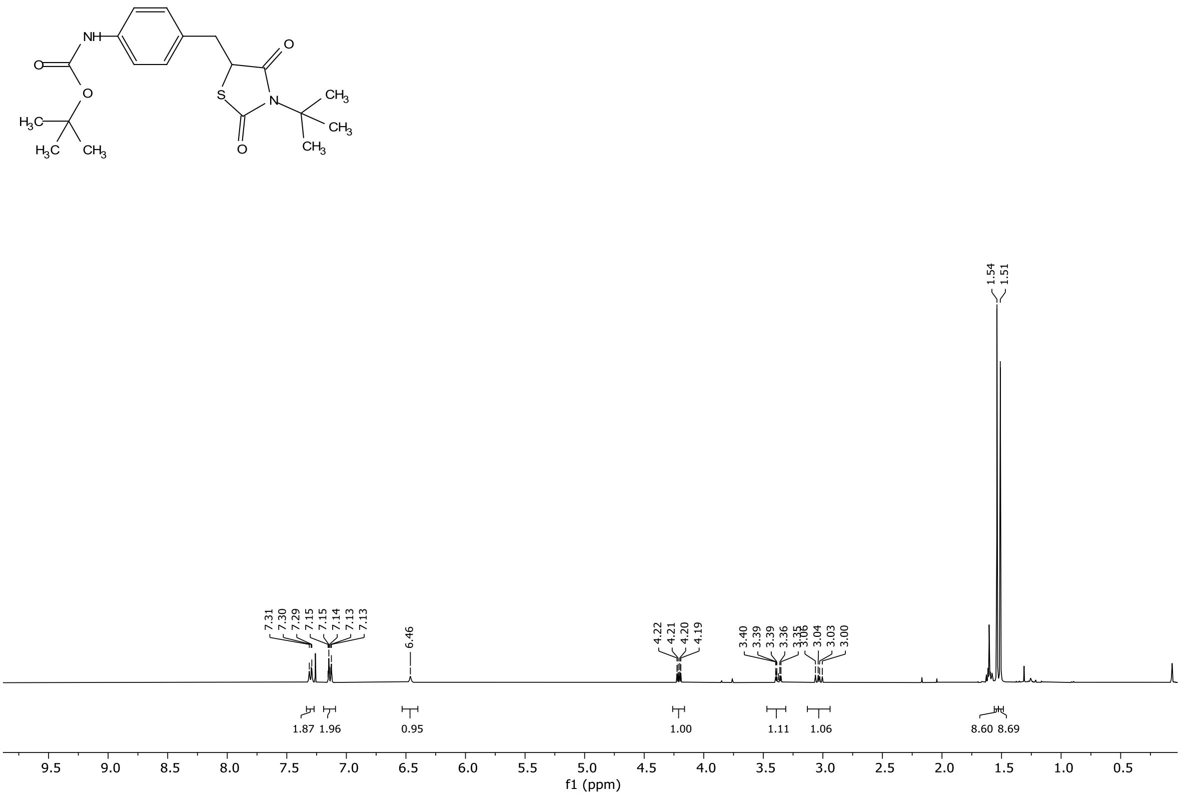

23, **^{13}C{1H} NMR** (101 MHz, $CDCl_3$)

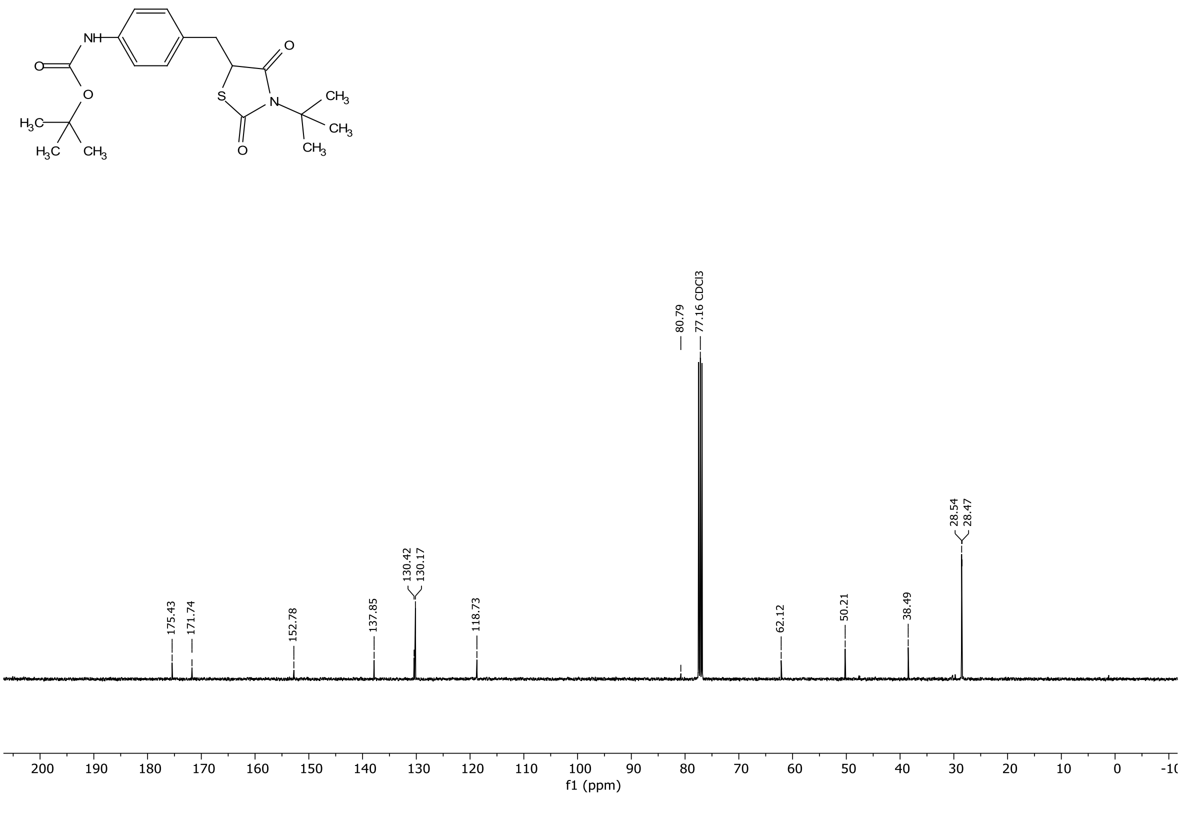

24, ^{1}H NMR (400 MHz, $CDCl_3$)

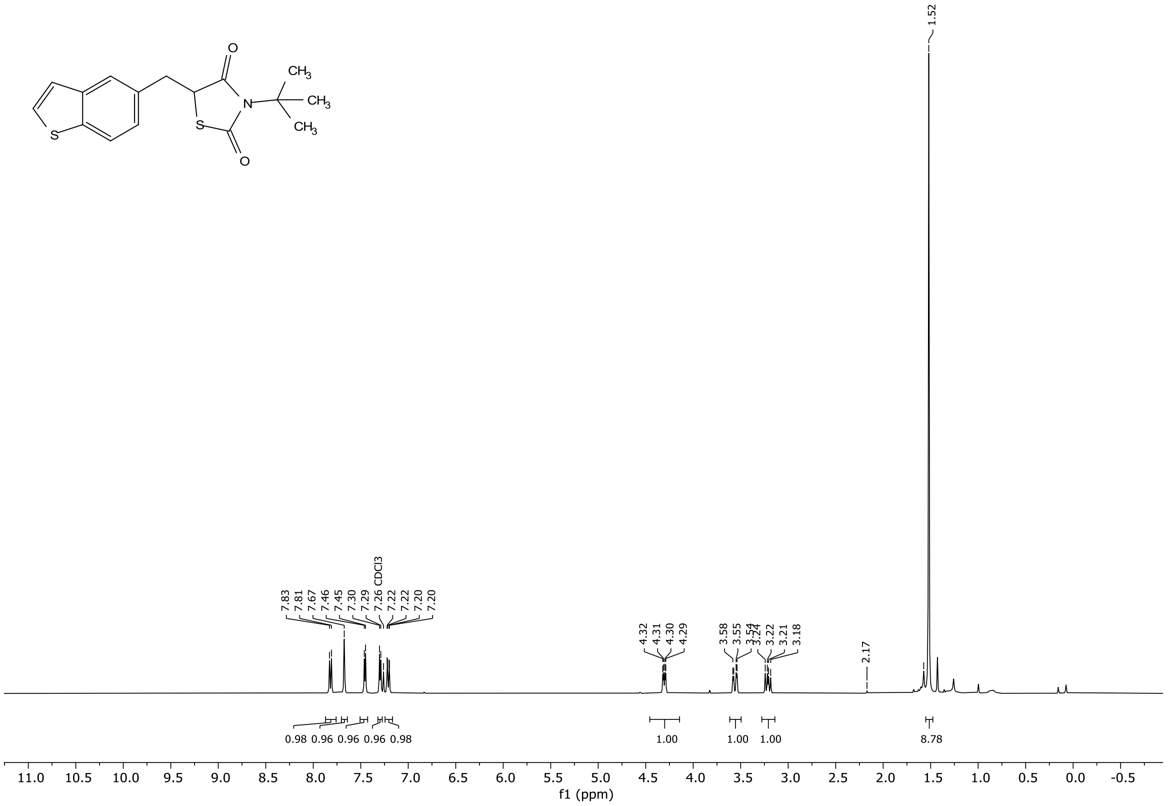

24, **^{13}C{1H} NMR** (101 MHz, $CDCl_3$)

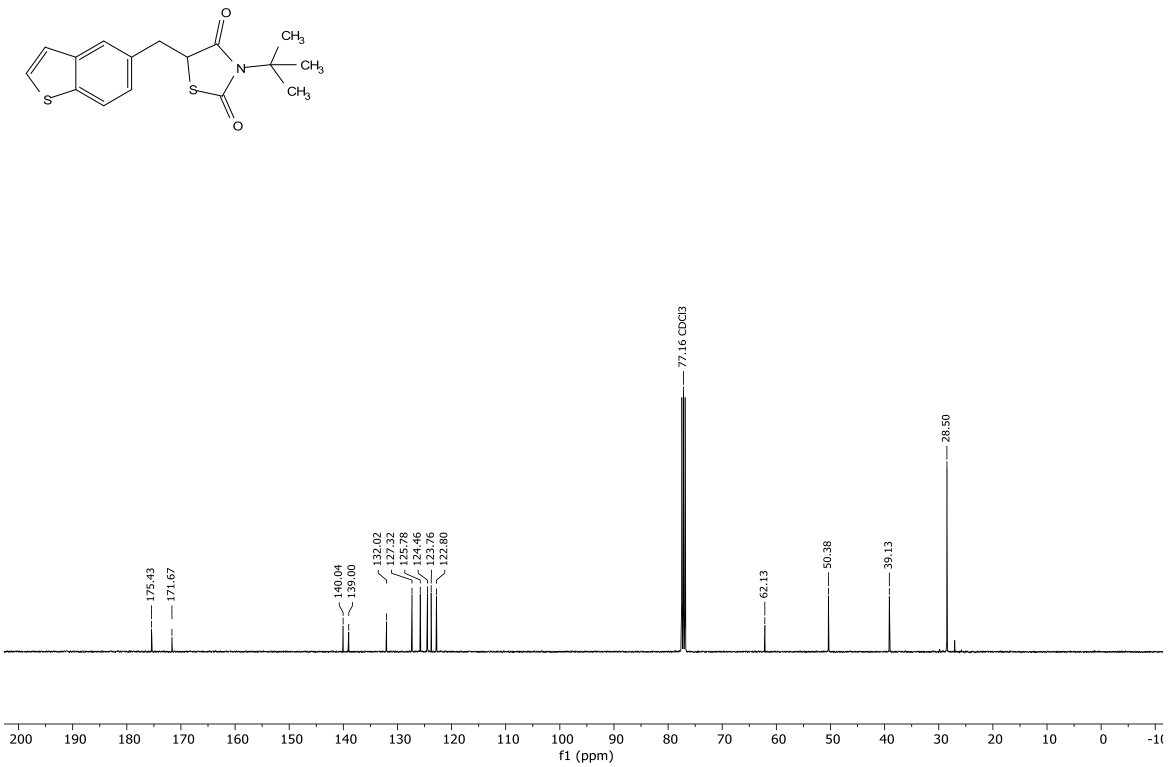

25, ^{1}H NMR (400 MHz, CDCl$_3$)

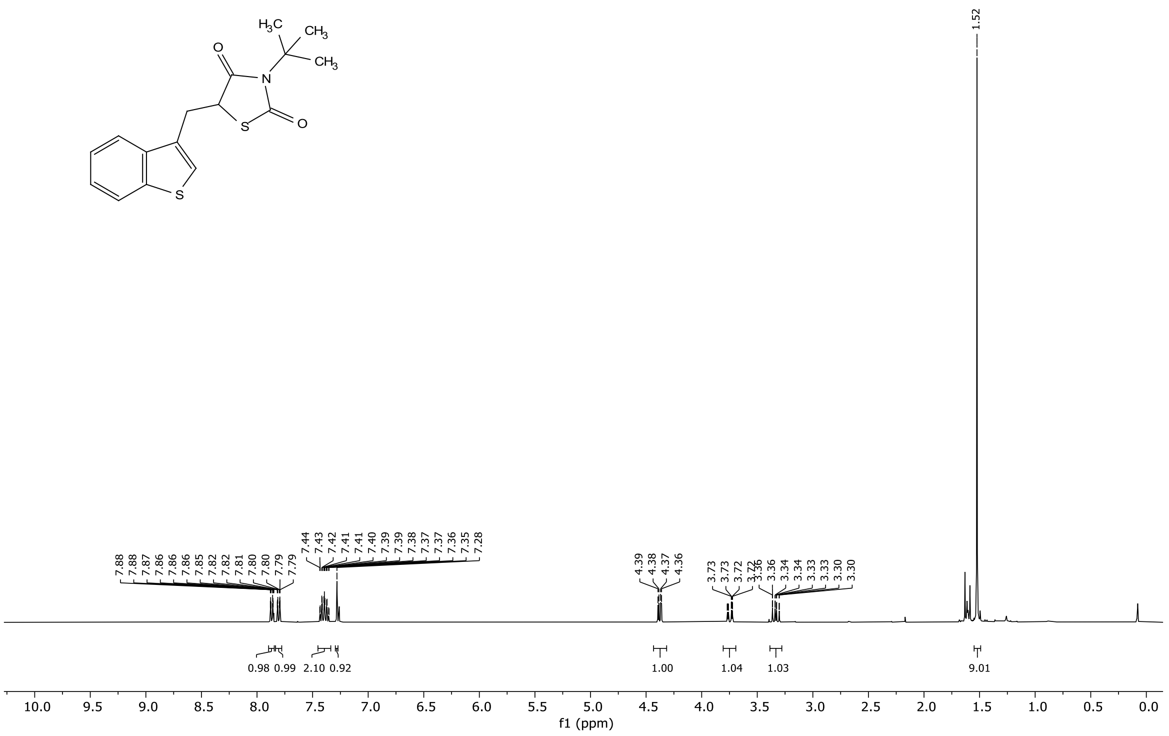

25, **^{13}C{1H} NMR** (101 MHz, CDCl$_3$)

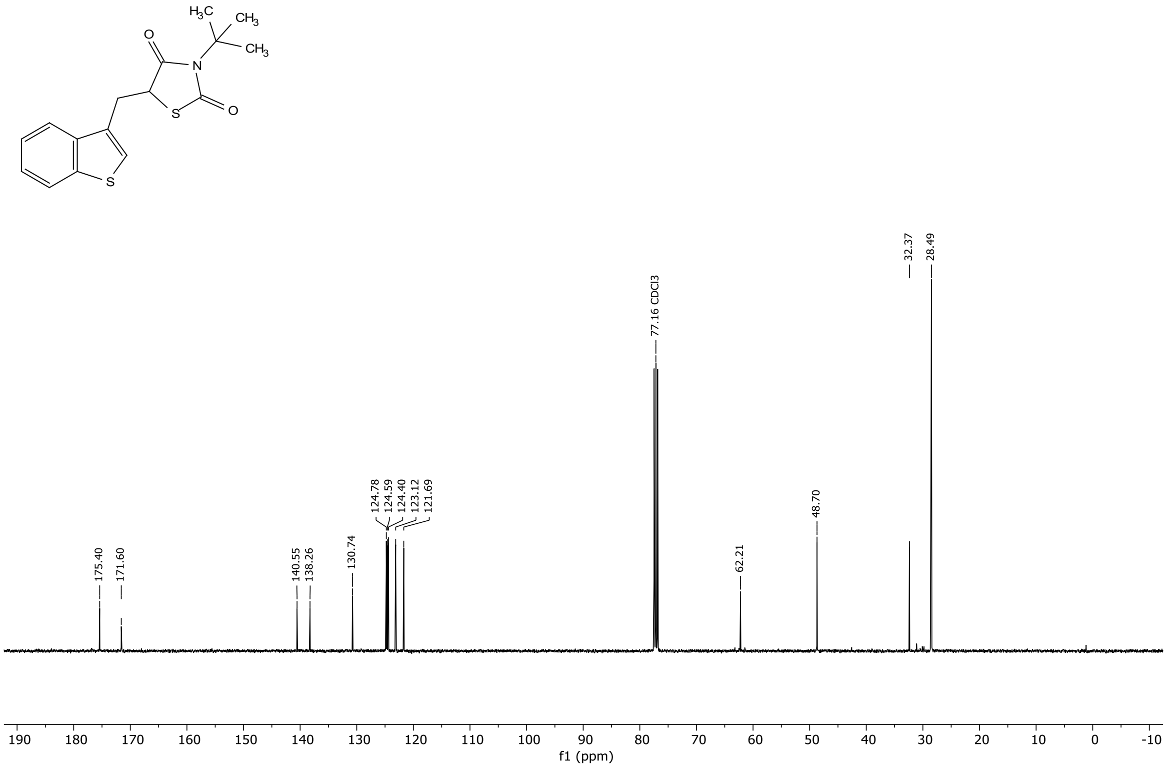

26, ¹H NMR (400 MHz, $CDCl_3$)

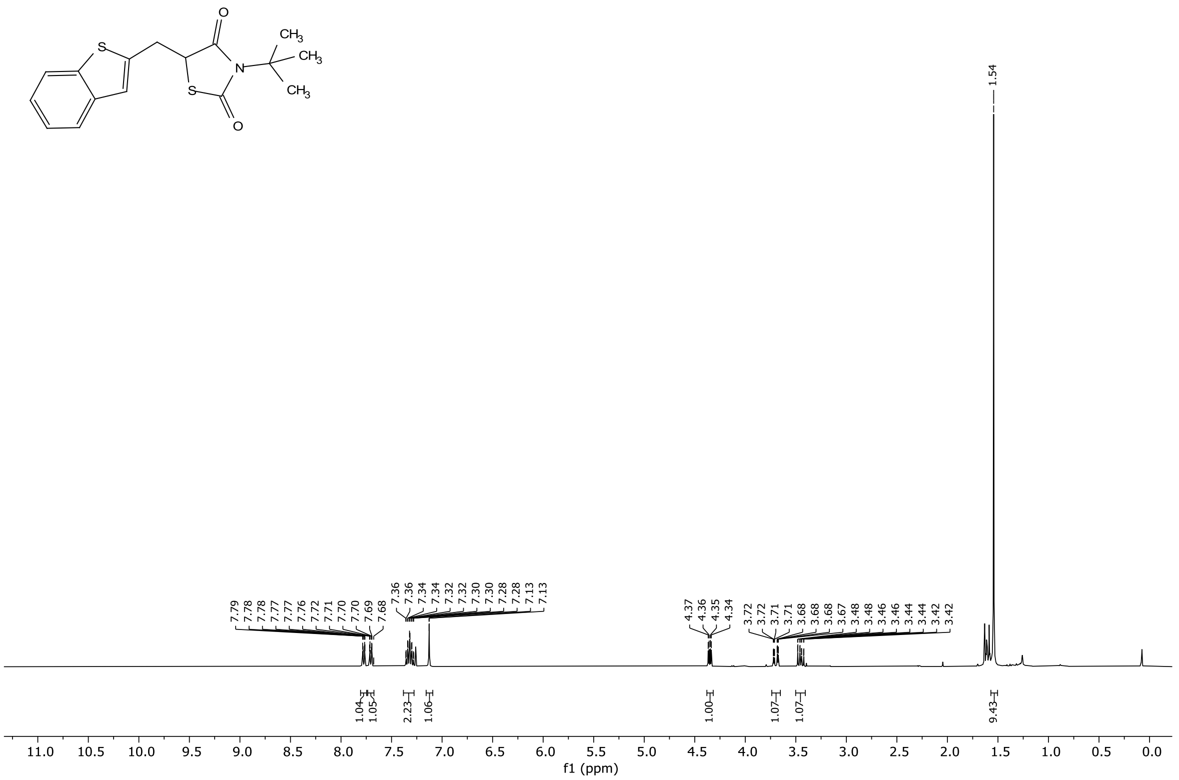

26, **^{13}C{1H} NMR** (101 MHz, $CDCl_3$)

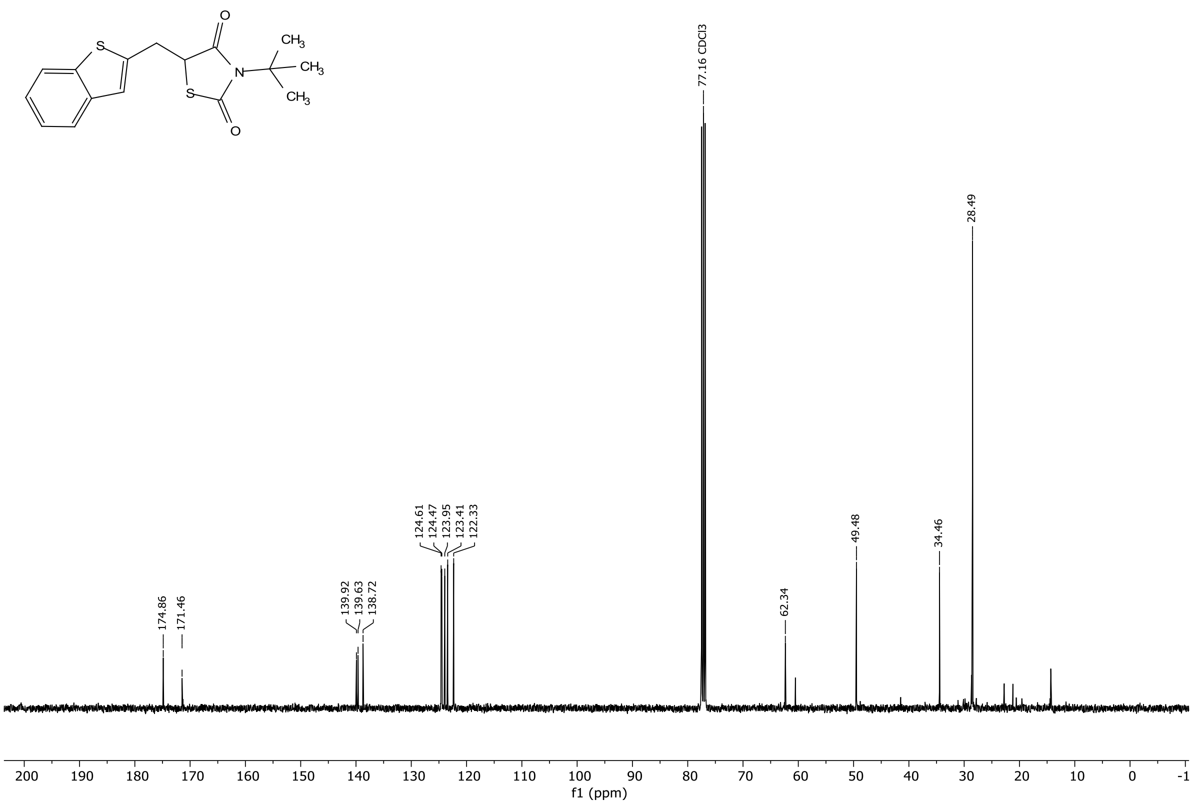

27, ^{1}H NMR (400 MHz, $CDCl_3$)

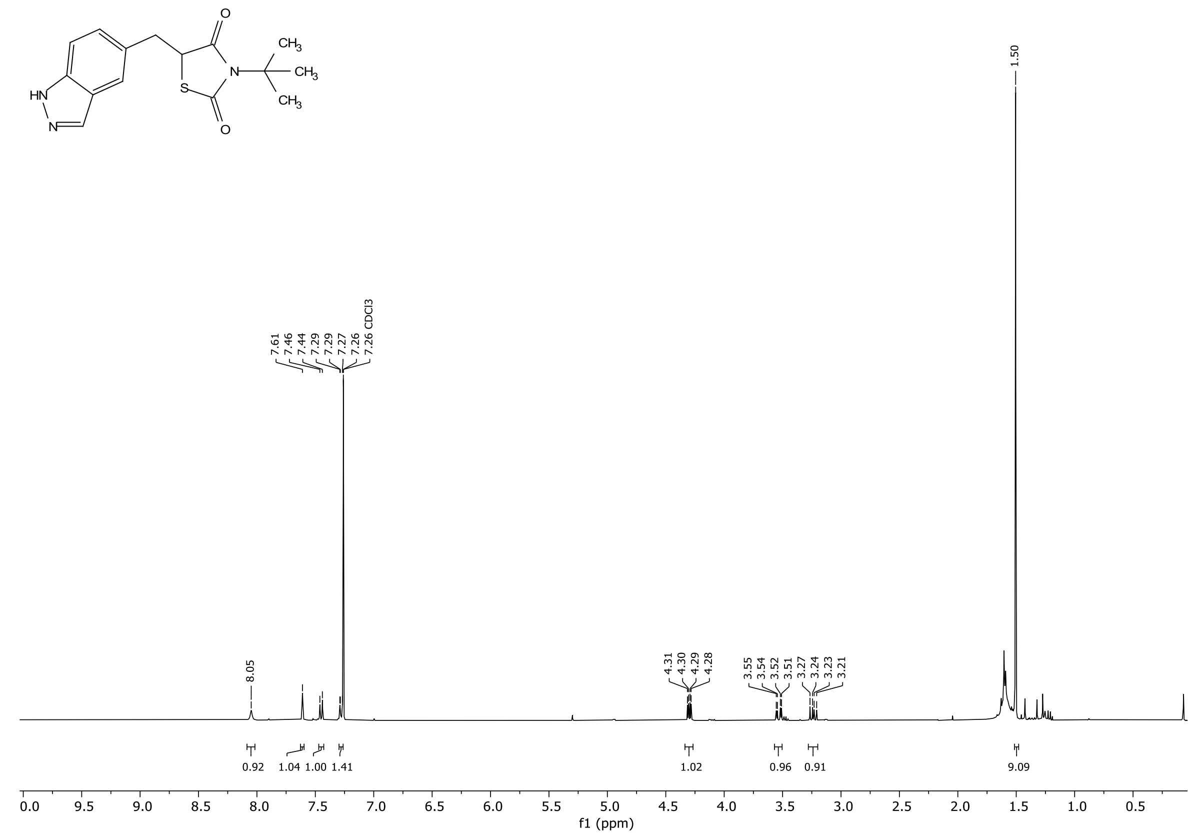

27, **^{13}C{1H} NMR** (101 MHz, $CDCl_3$)

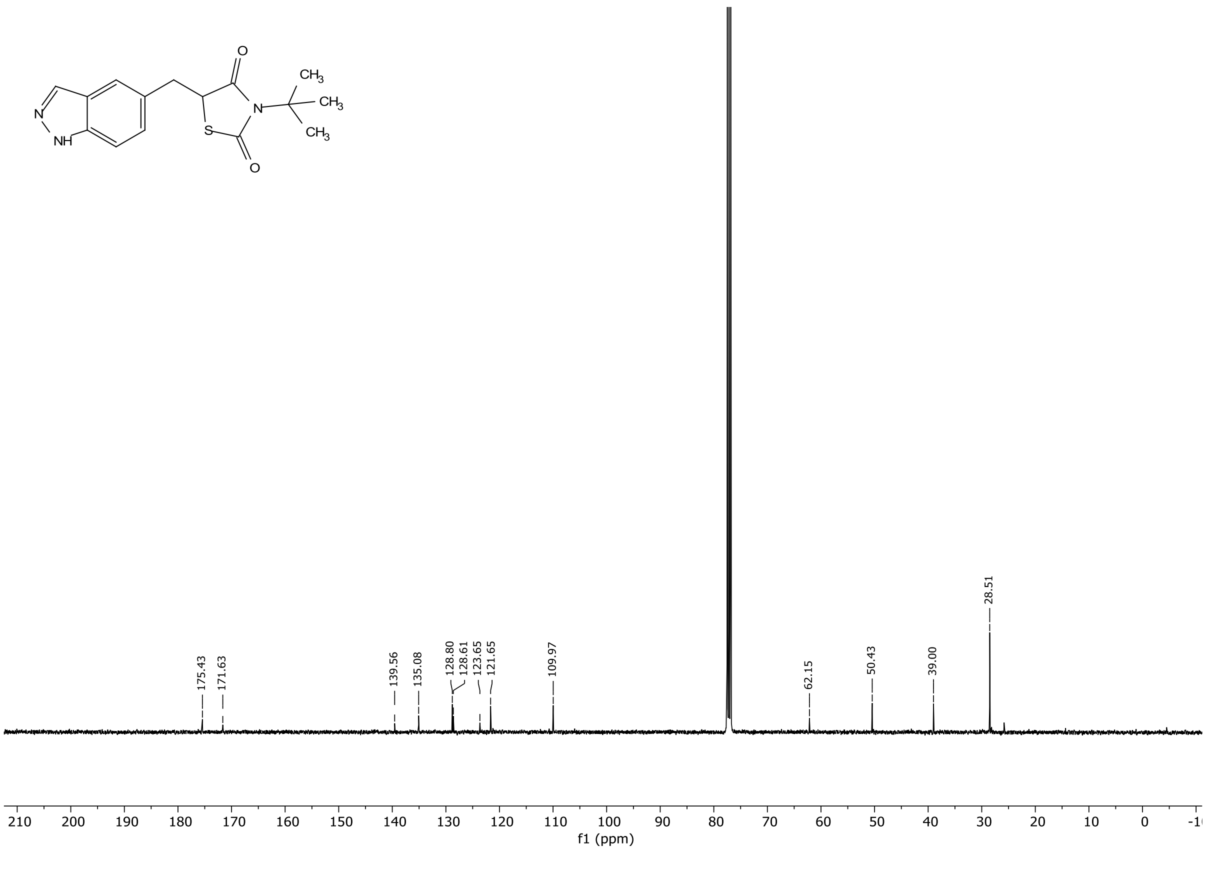

28, ^{1}H NMR (400 MHz, $CDCl_3$)

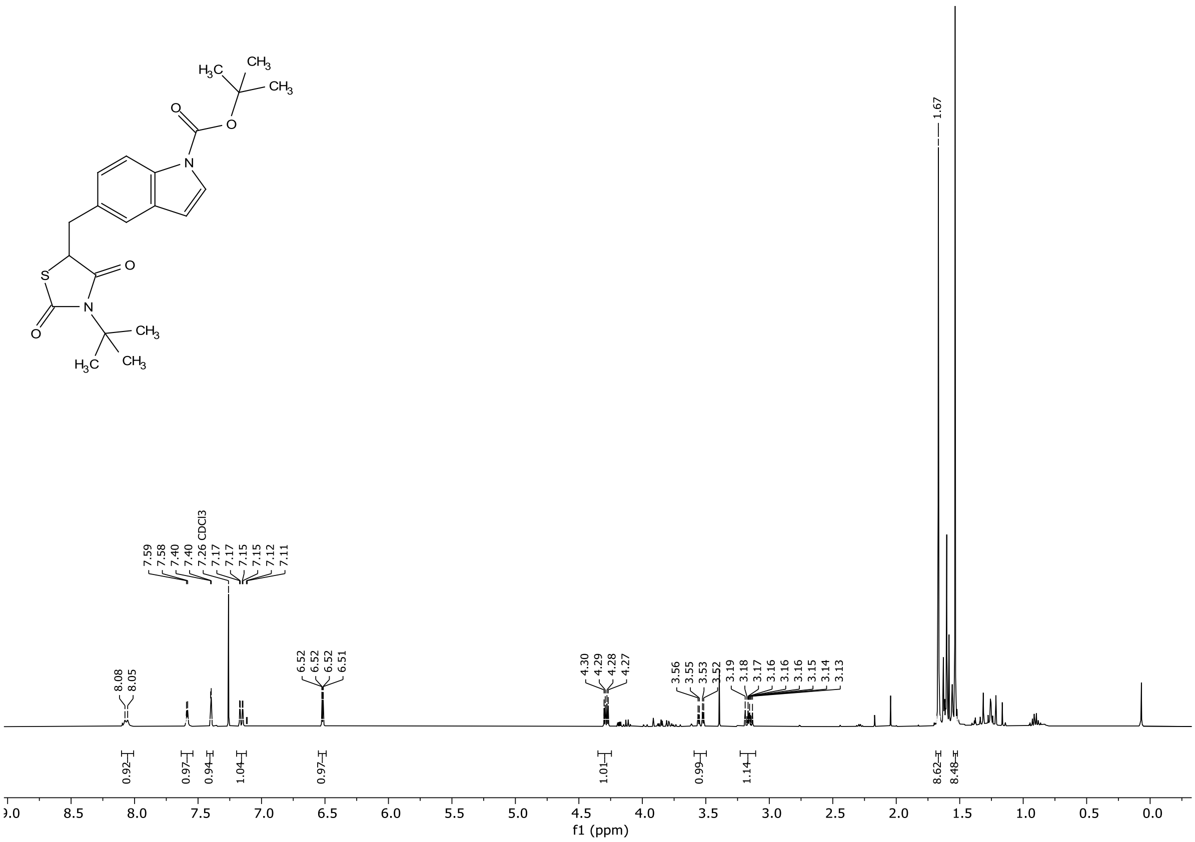

28, **^{13}C{1H} NMR** (101 MHz, $CDCl_3$)

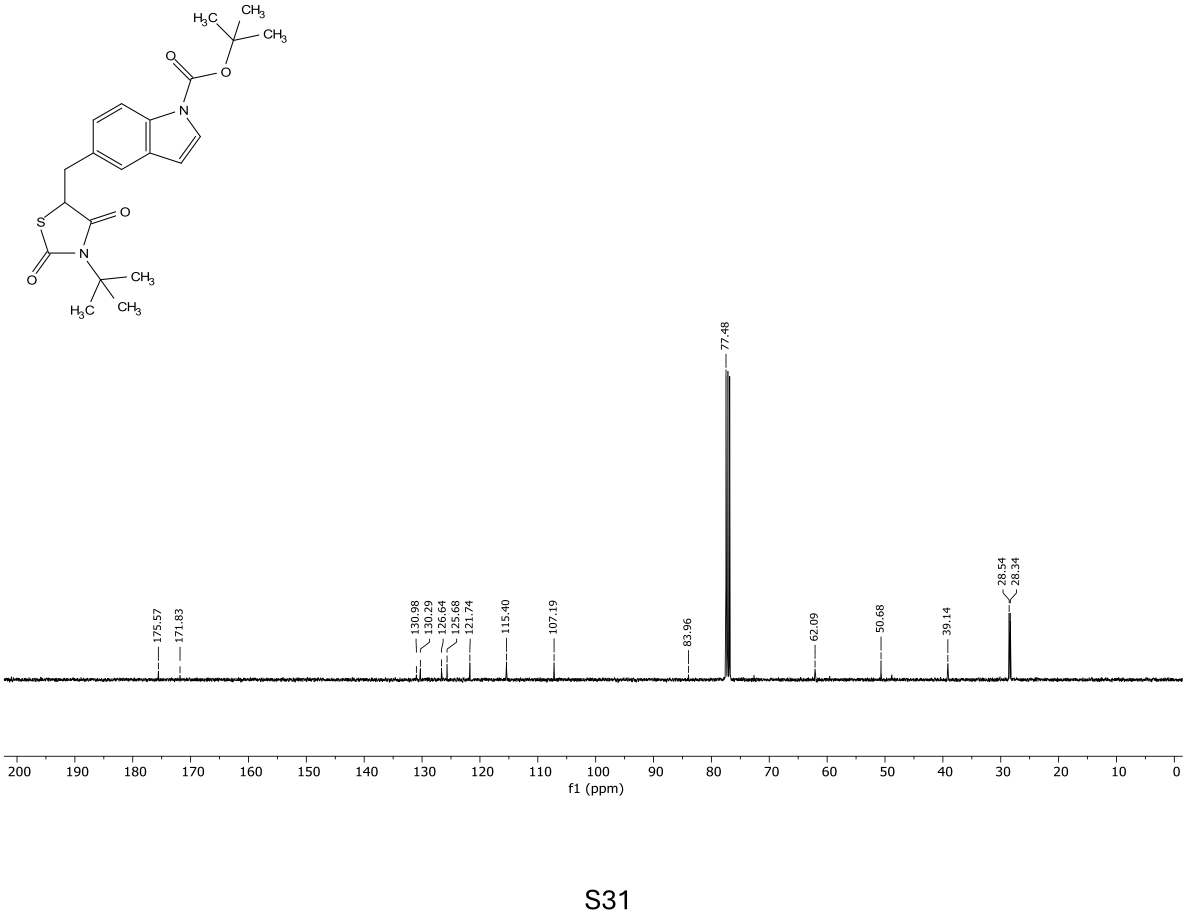

29, ^{1}H NMR (400 MHz, $CDCl_3$)

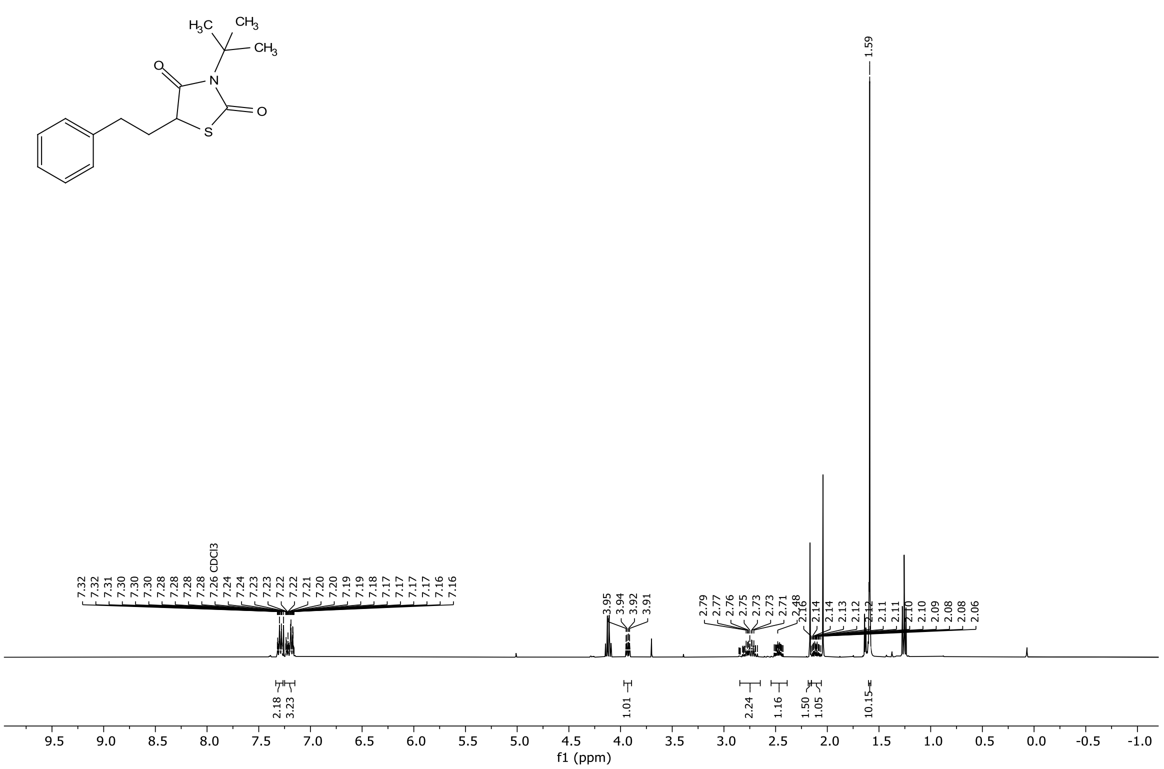

29, ^{13}**C{1H} NMR** (101 MHz, $CDCl_3$)

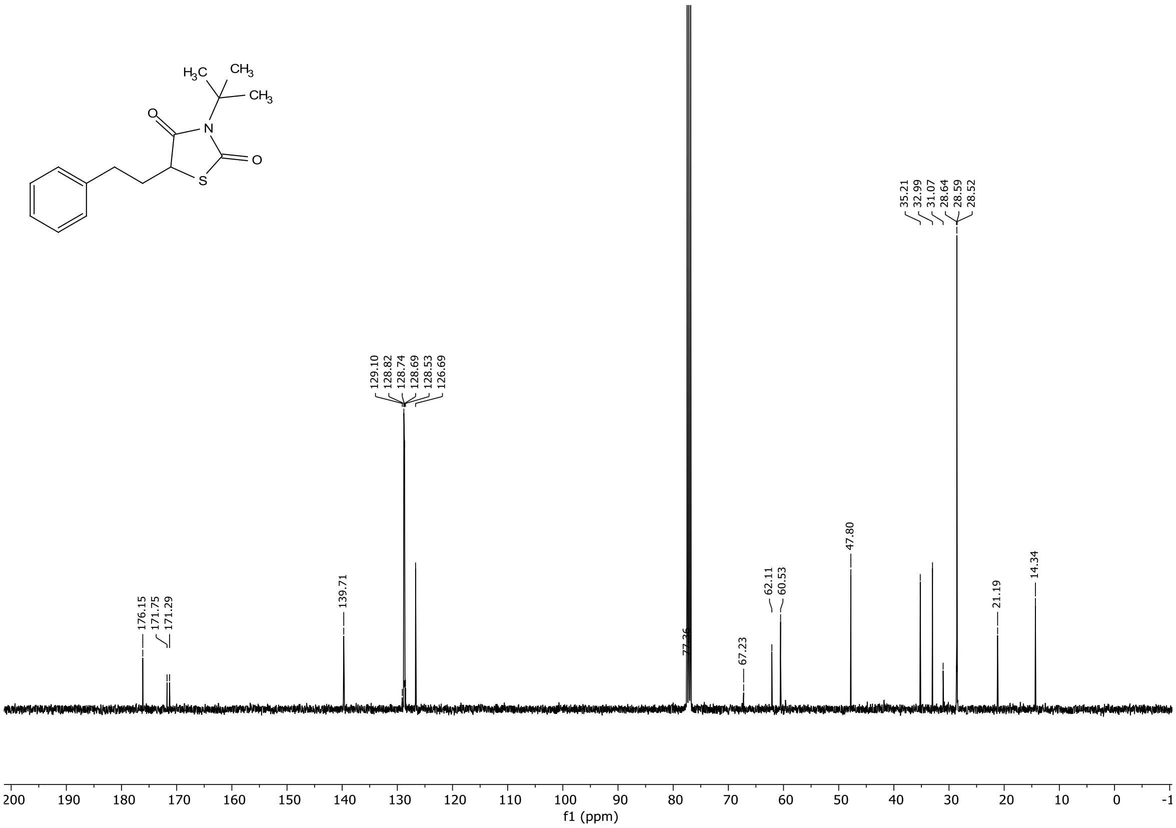

30, ^{1}H NMR (400 MHz, $CDCl_3$)

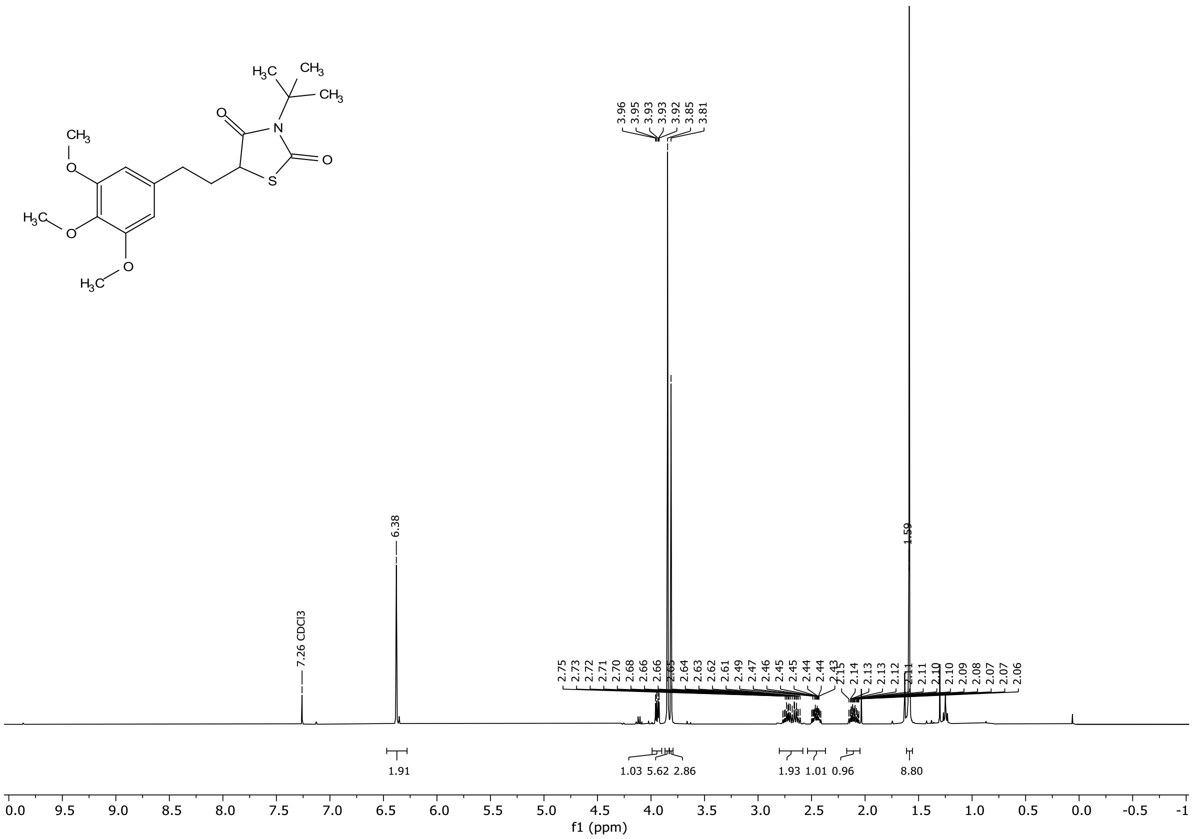

30, **^{13}C{1H} NMR** (101 MHz, $CDCl_3$)

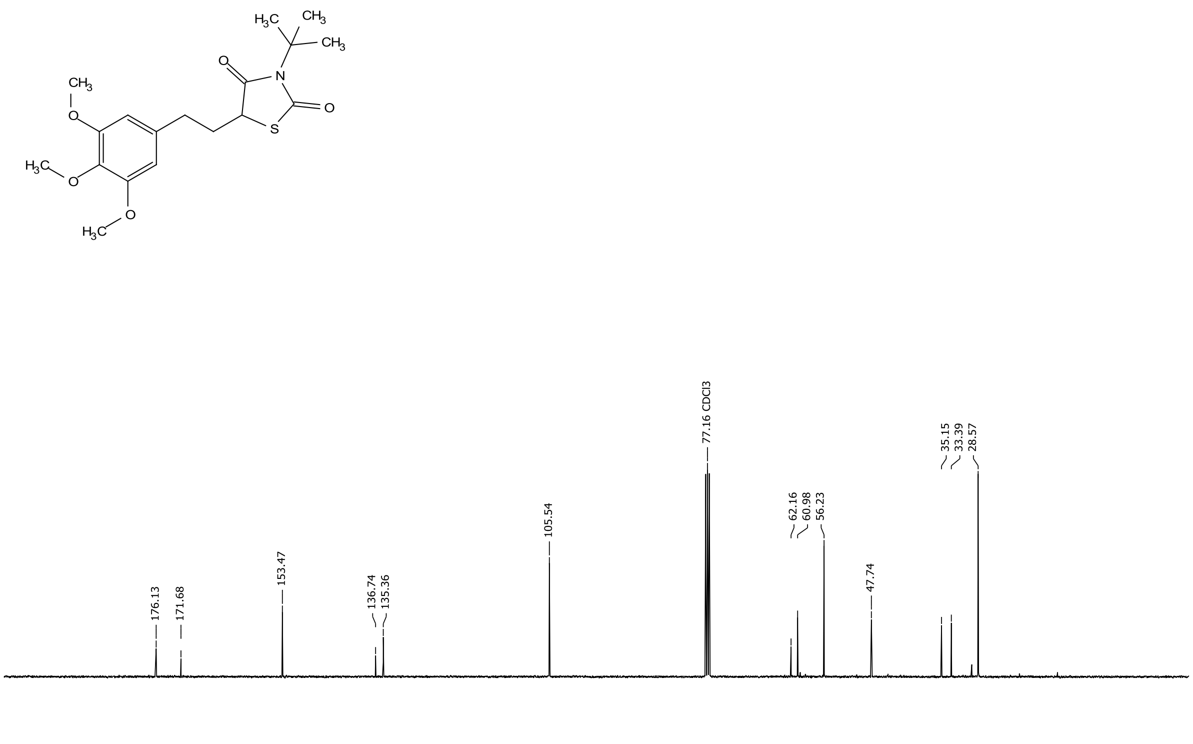

31, ^{1}H NMR (400 MHz, $CDCl_3$)

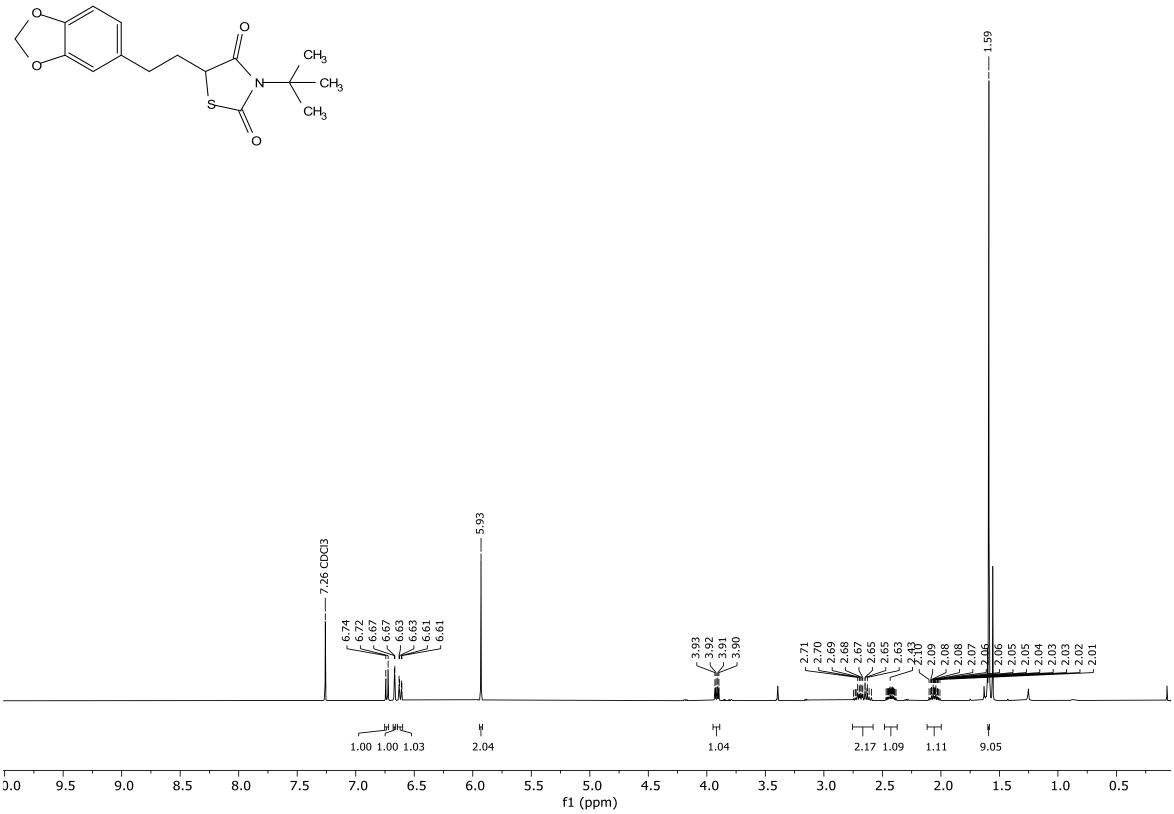

31, **^{13}C{1H} NMR** (101 MHz, $CDCl_3$)

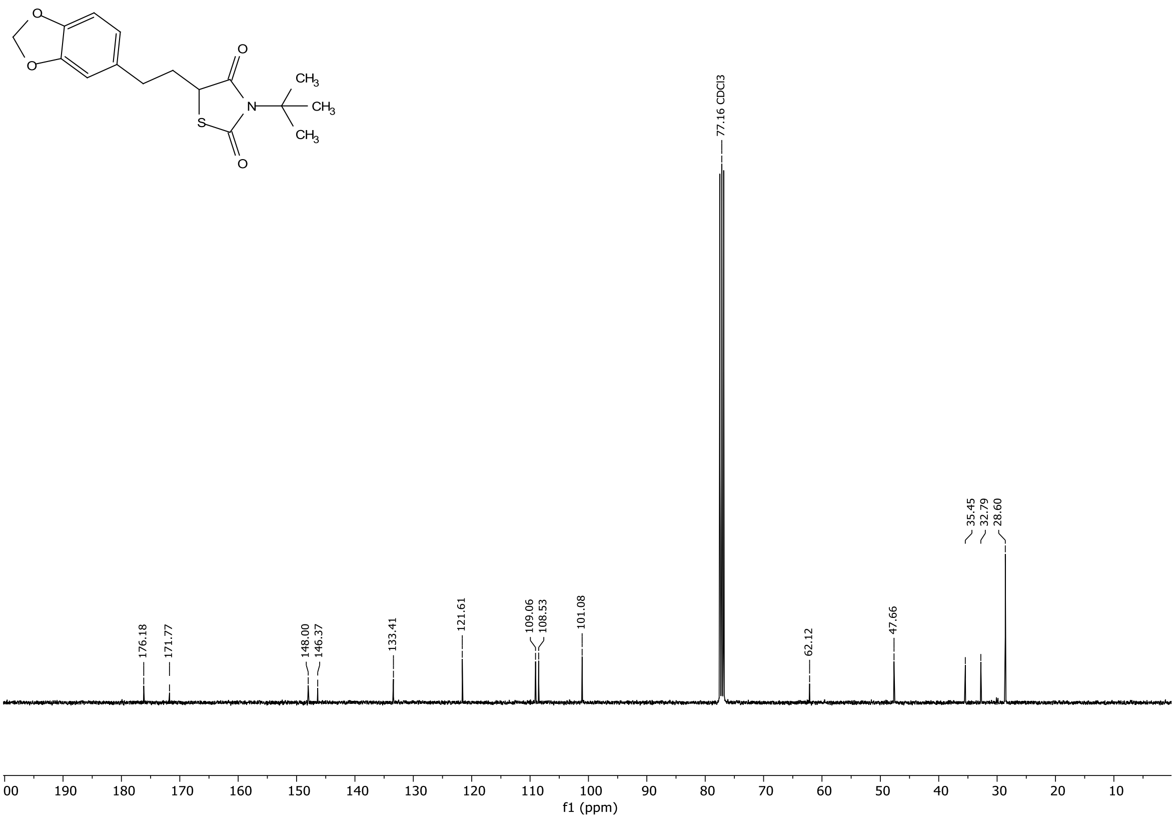

32, ^{1}H NMR (400 MHz, DMSO-d_6)

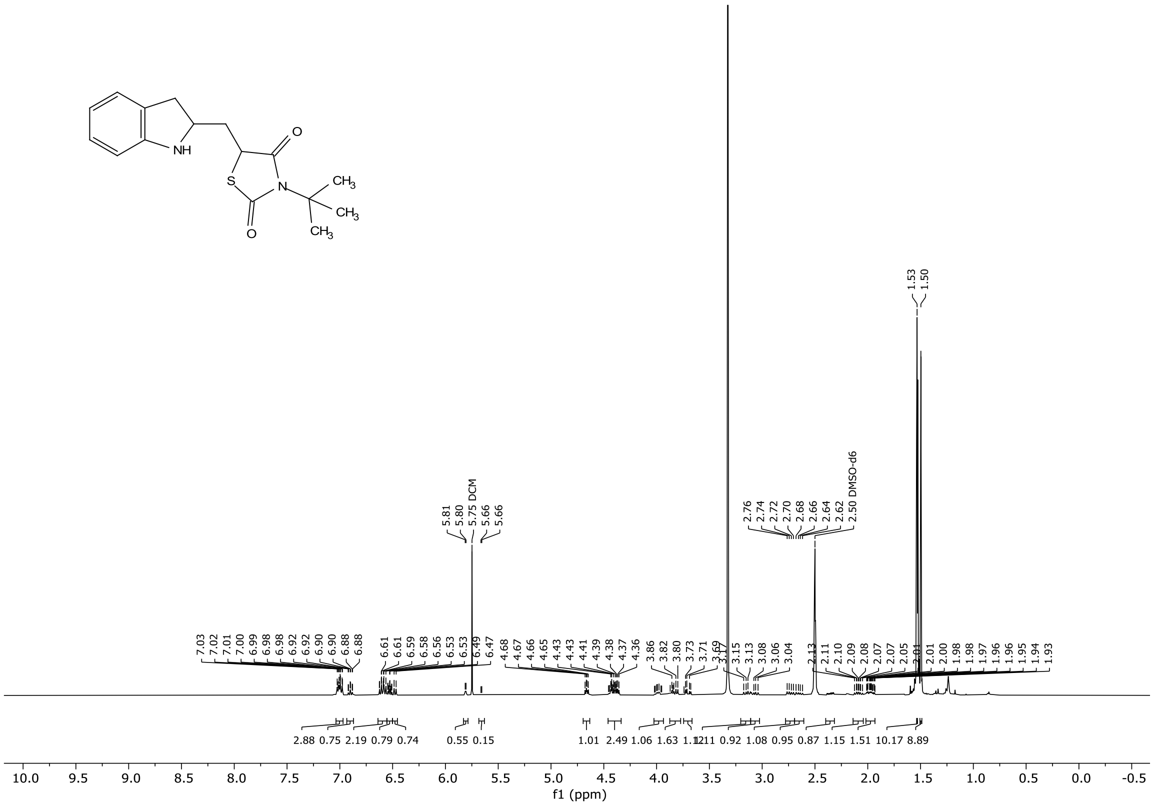

32, **^{13}C{1H} NMR** (101 MHz, $CDCl_3$)

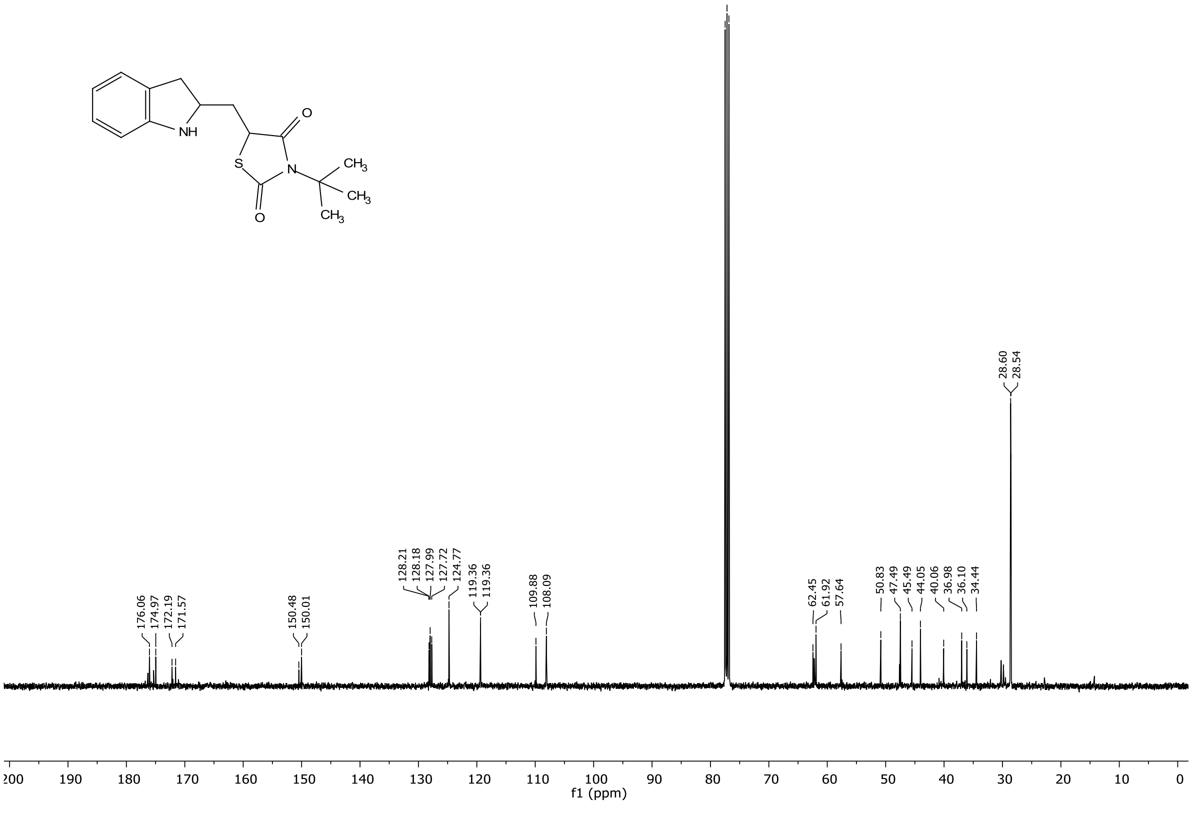

33, ^{1}H NMR (400 MHz, $CDCl_3$)

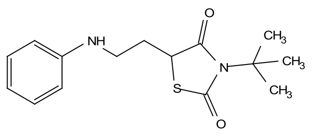

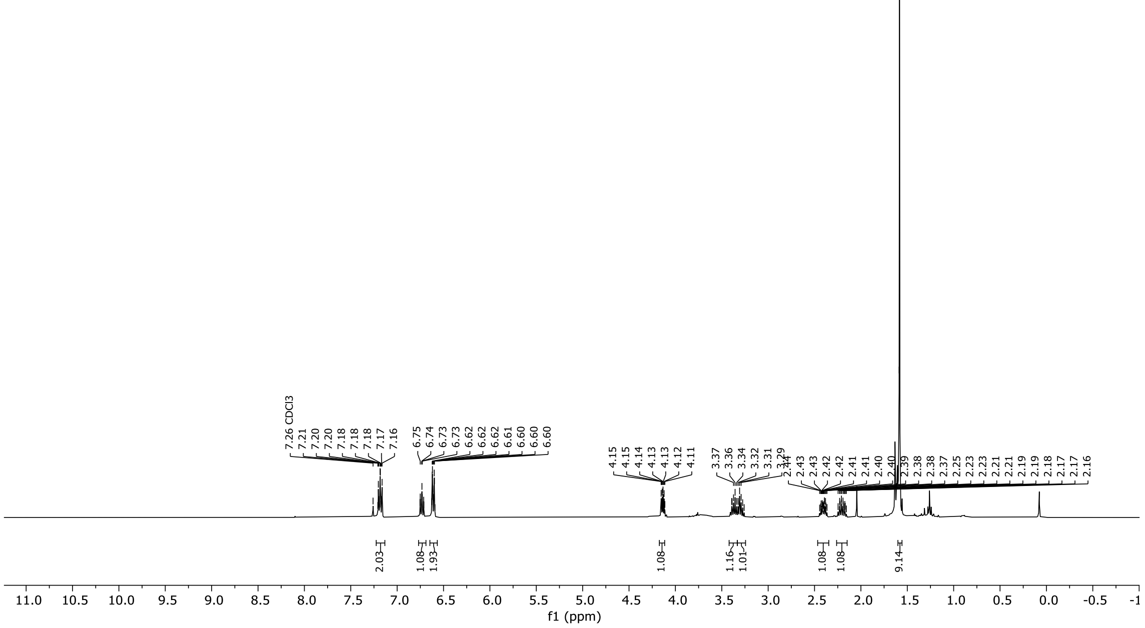

33, **^{13}C{1H} NMR** (101 MHz, $CDCl_3$)

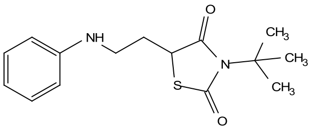

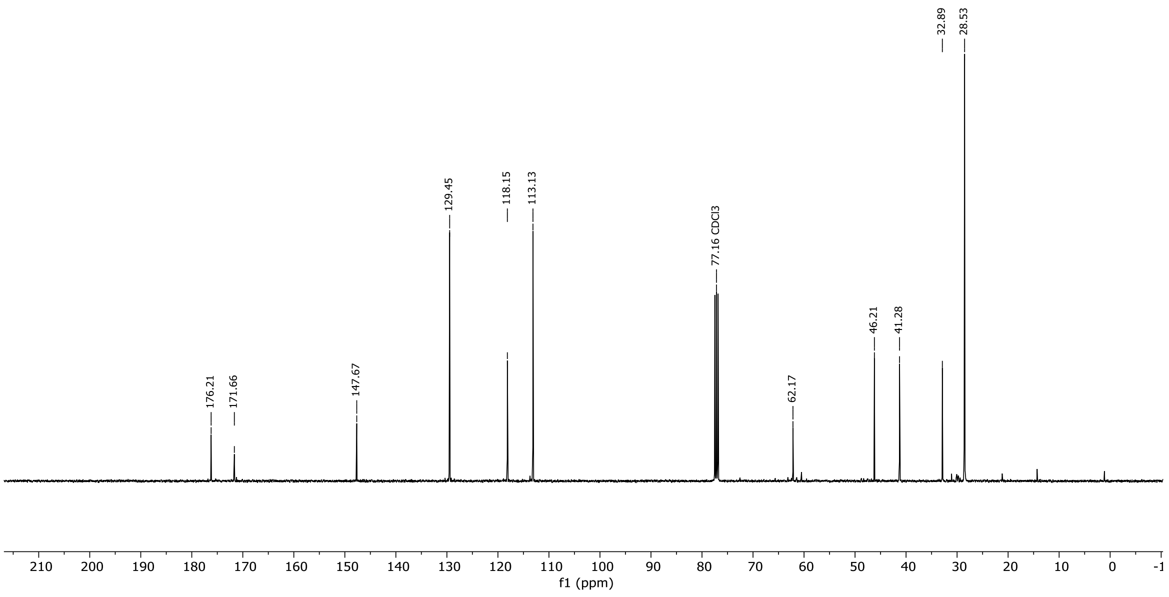

34, ^{1}H NMR (400 MHz, $CDCl_3$)

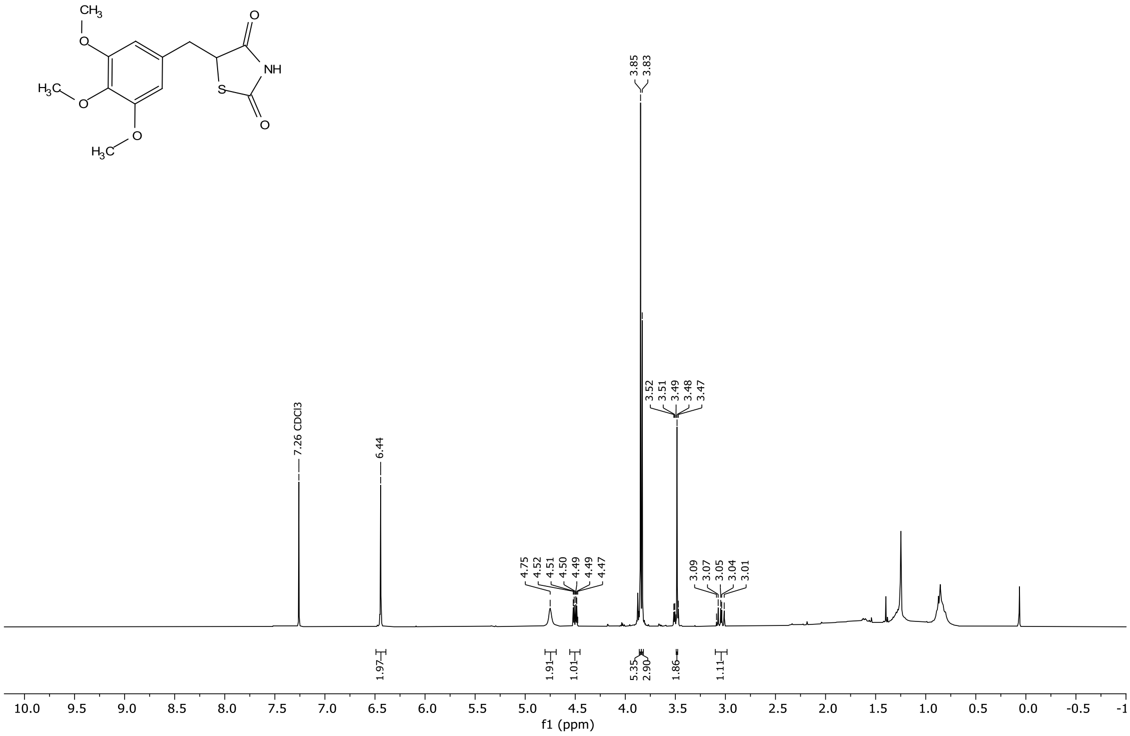

34, **^{13}C{1H} NMR** (101 MHz, $CDCl_3$)

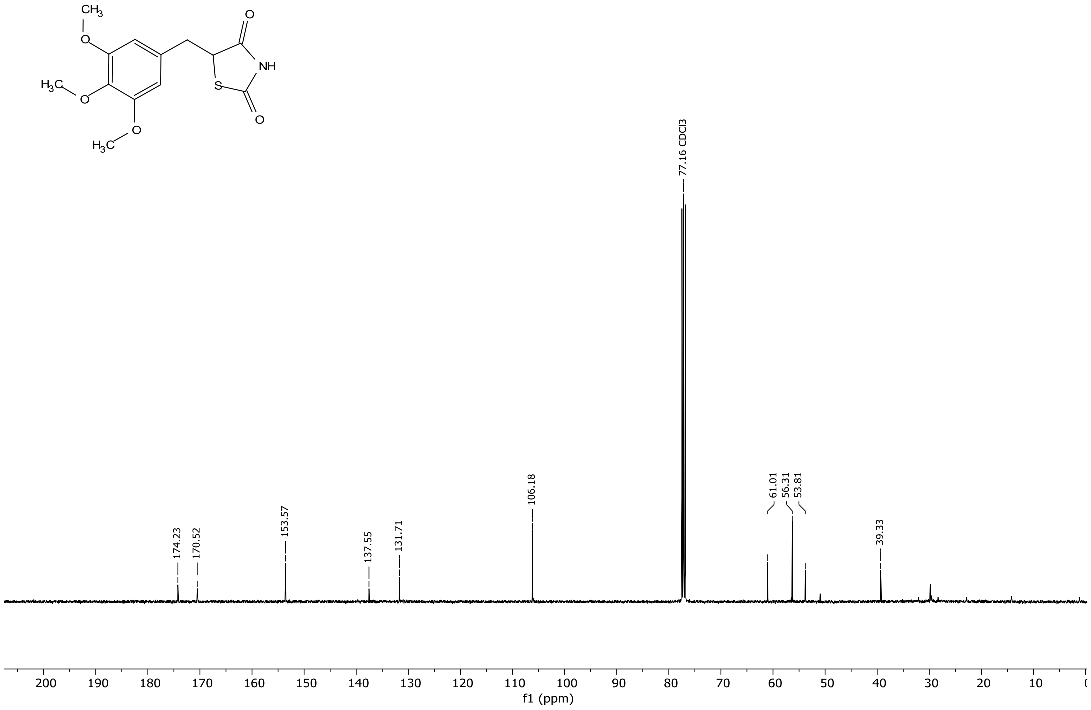

35, ^{1}H NMR (400 MHz, $CDCl_3$)

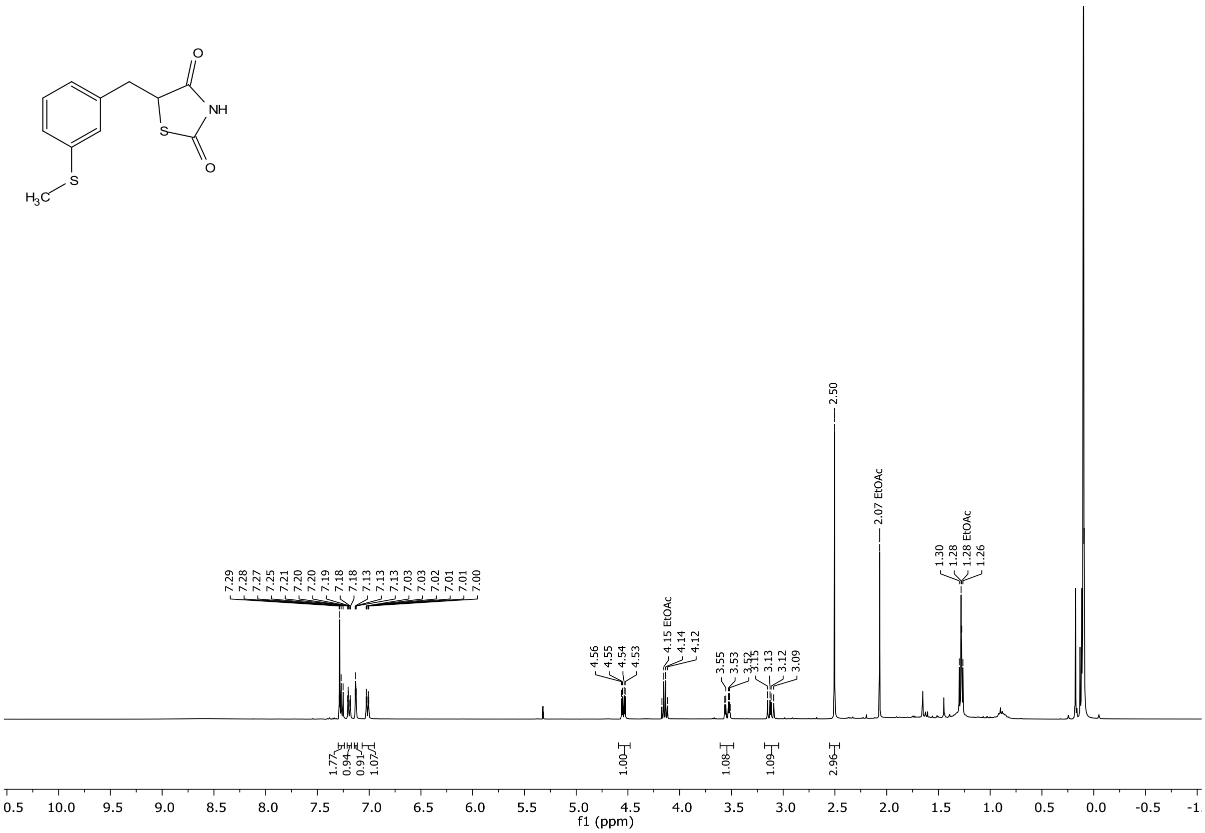

35, ^{13}**C{1H} NMR** (101 MHz, $CDCl_3$)

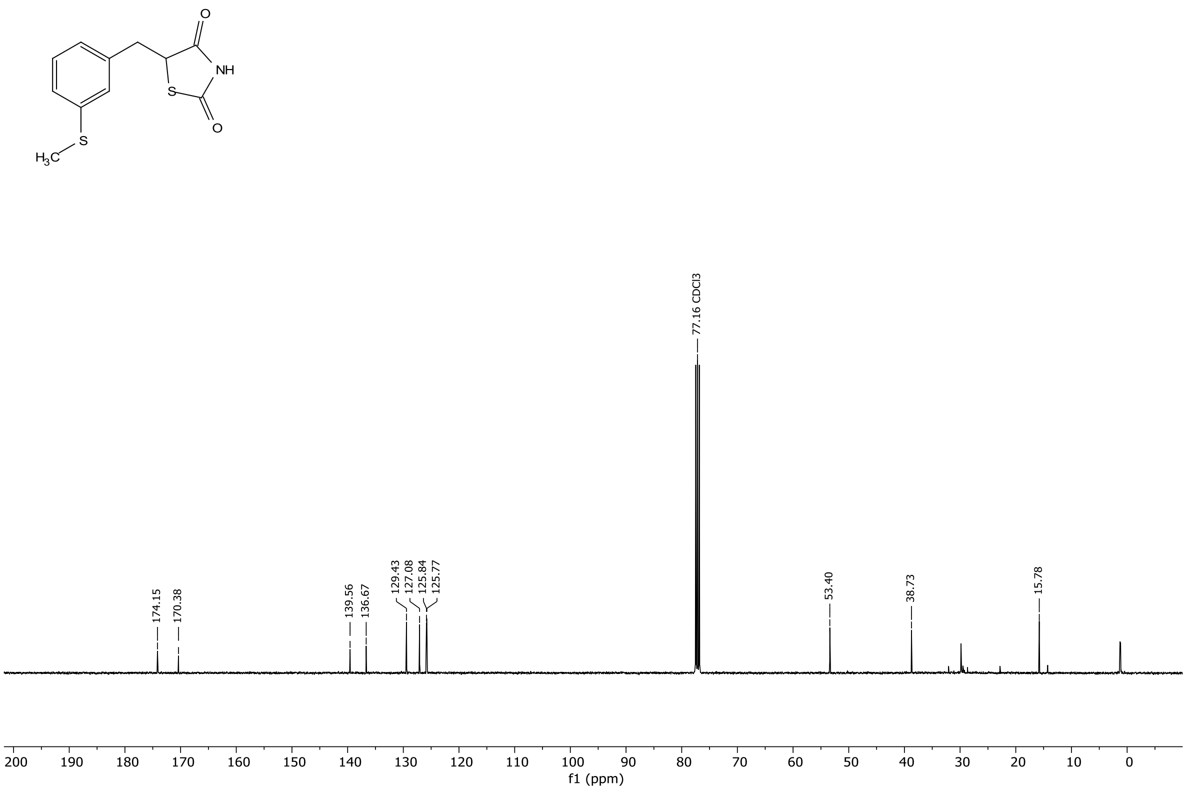

36, ^{1}H NMR (400 MHz, Acetone-d_6)

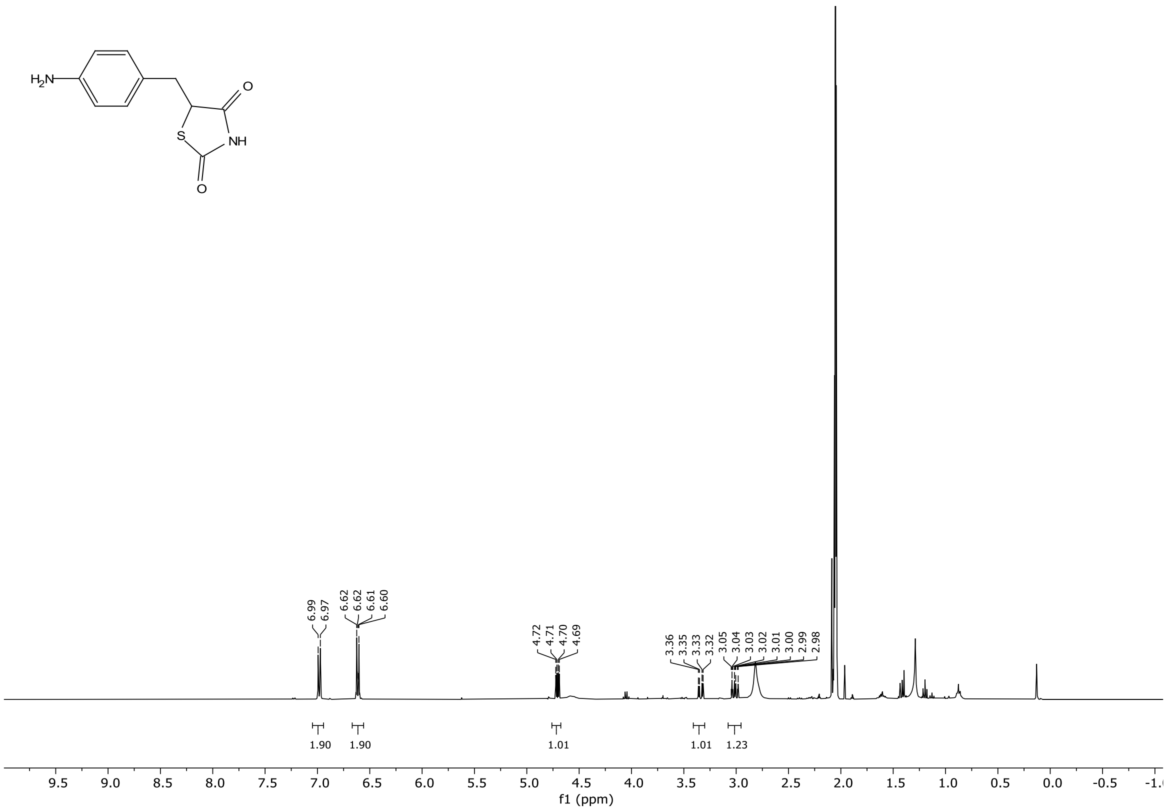

36, **^{13}C{1H} NMR** (101 MHz, Acetone-d_6)

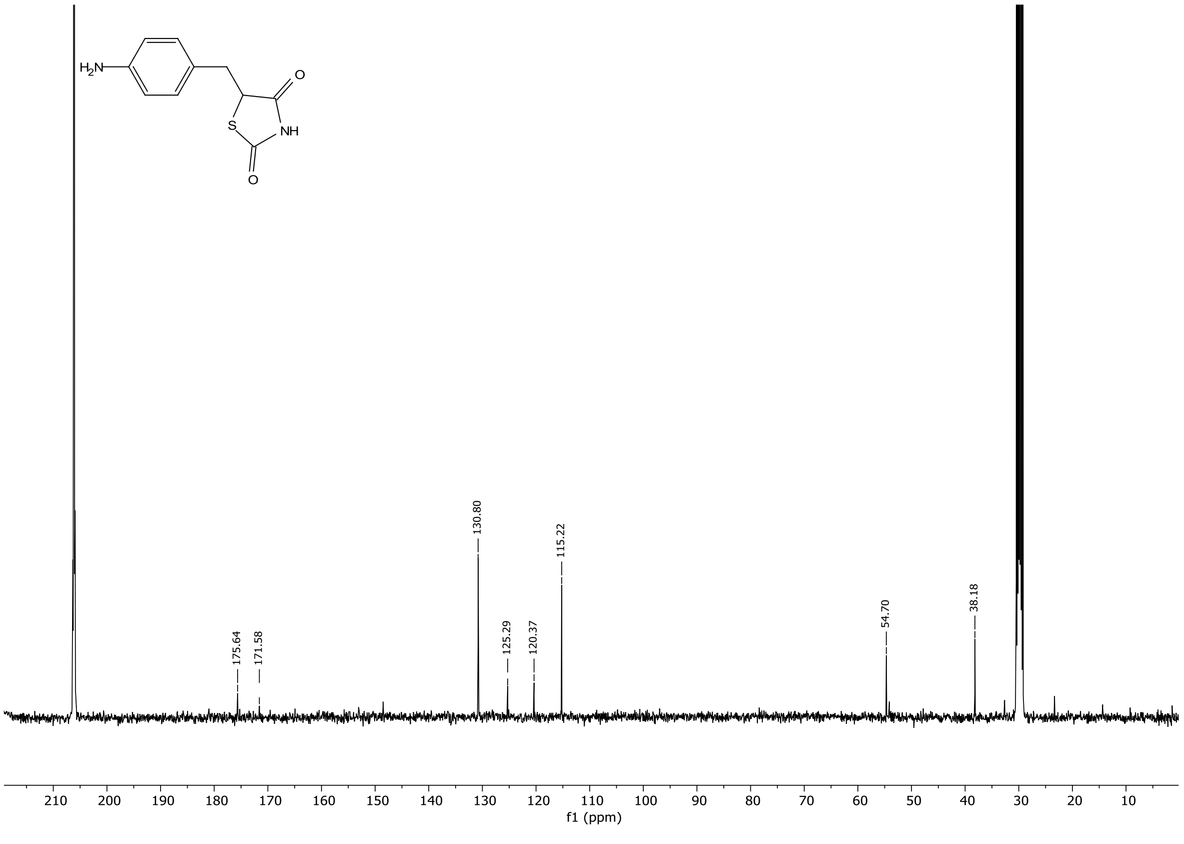

37, ^{1}H NMR (400 MHz, d_4-MeOH)

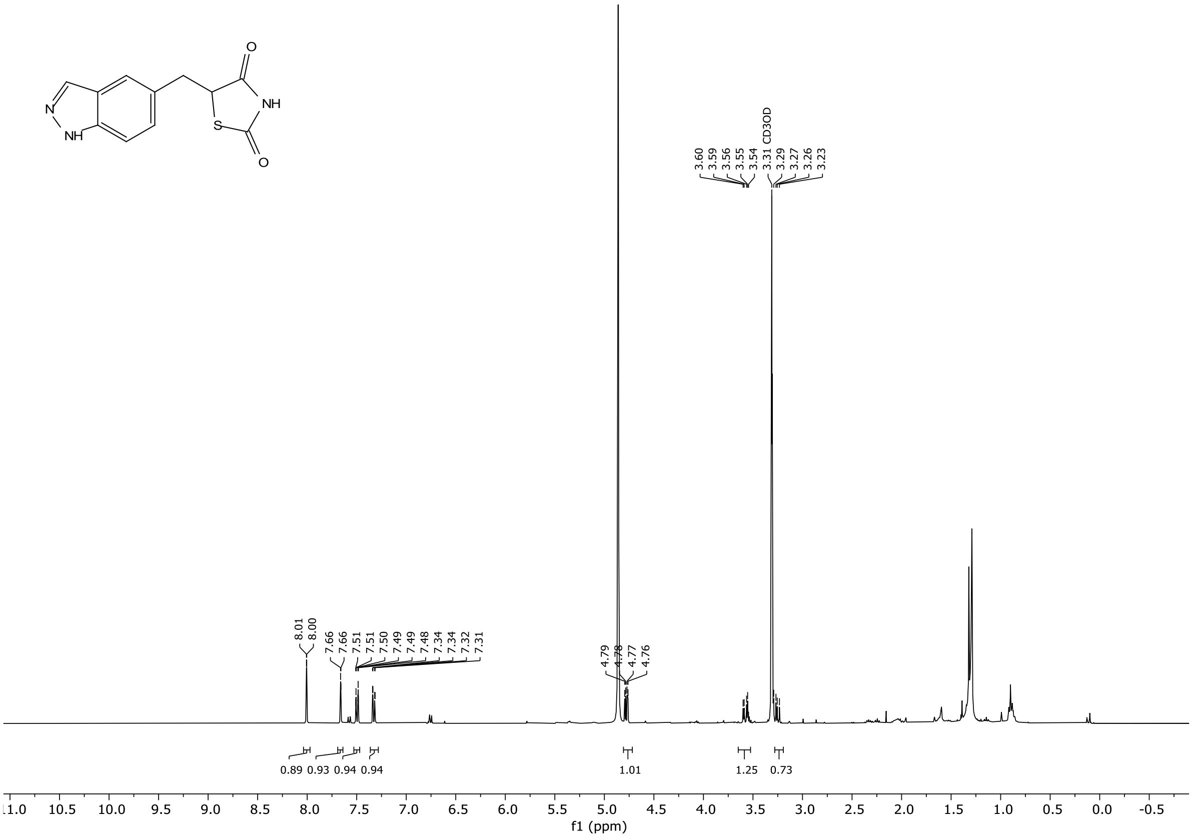

38, ^{1}H NMR (400 MHz, Acetone-d_6)

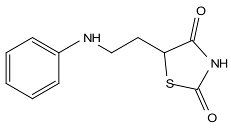

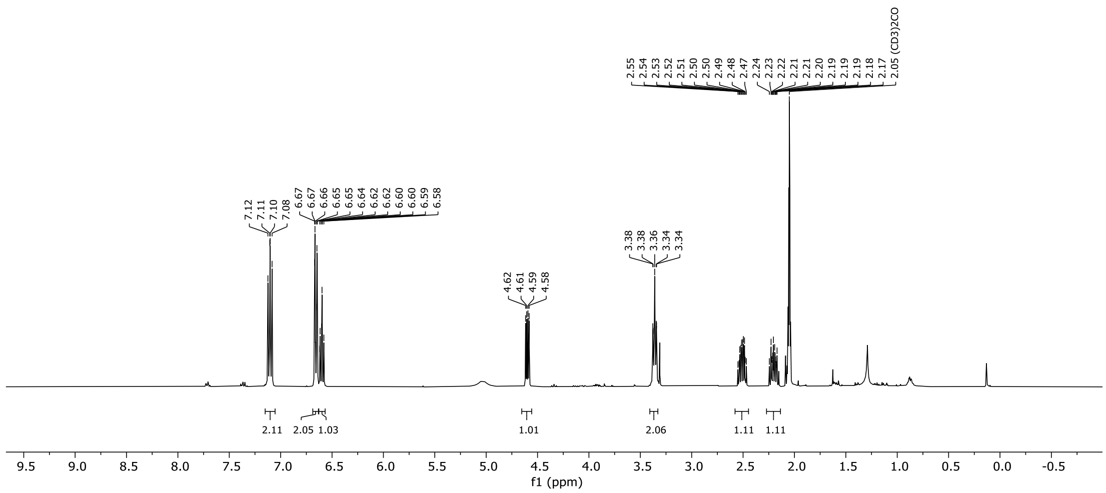

38, **^{13}C{1H} NMR** (101 MHz, Acetone-d_6)

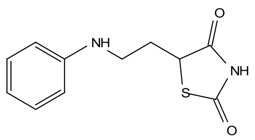

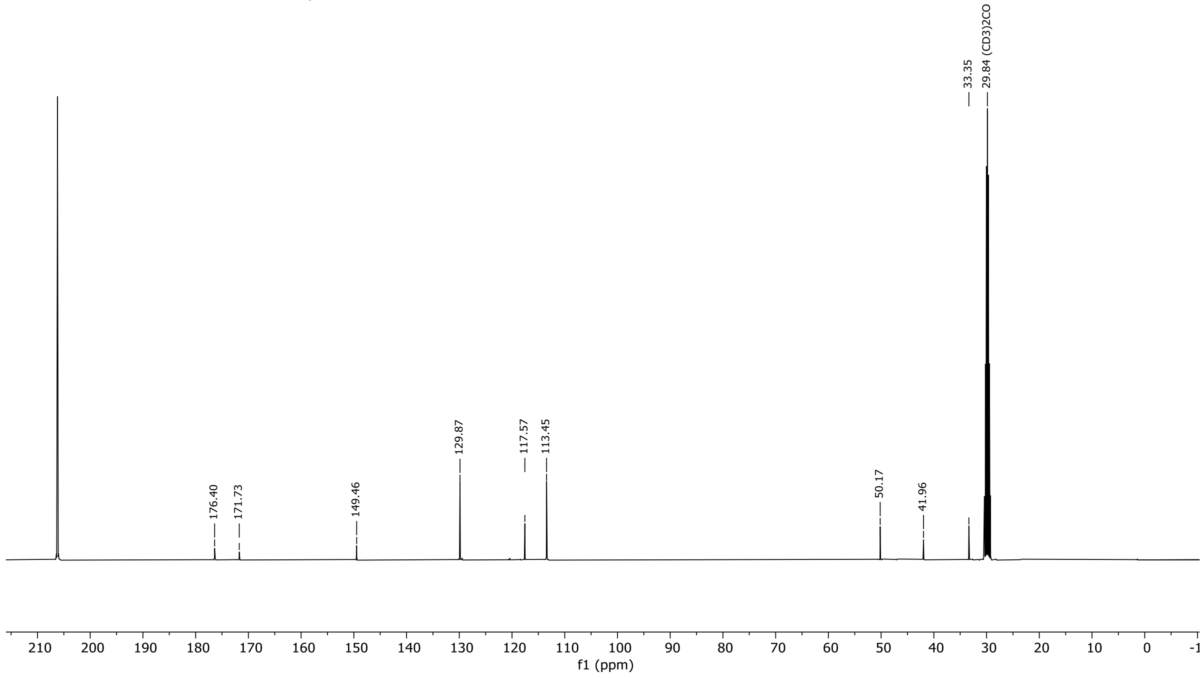

41, ^{1}H NMR (400 MHz, $CDCl_3$)

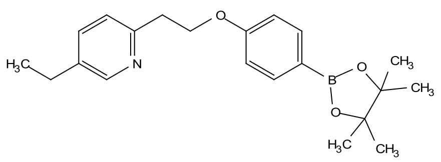

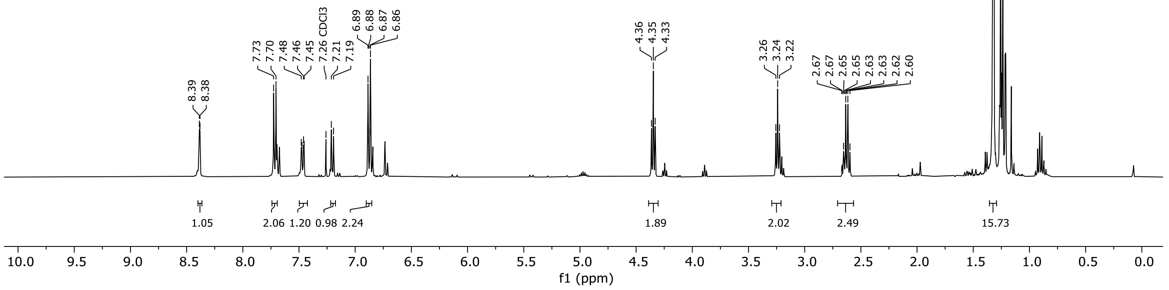

41, **^{13}C{1H} NMR** (101 MHz, $CDCl_3$)

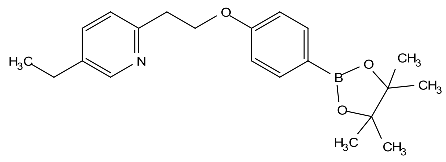

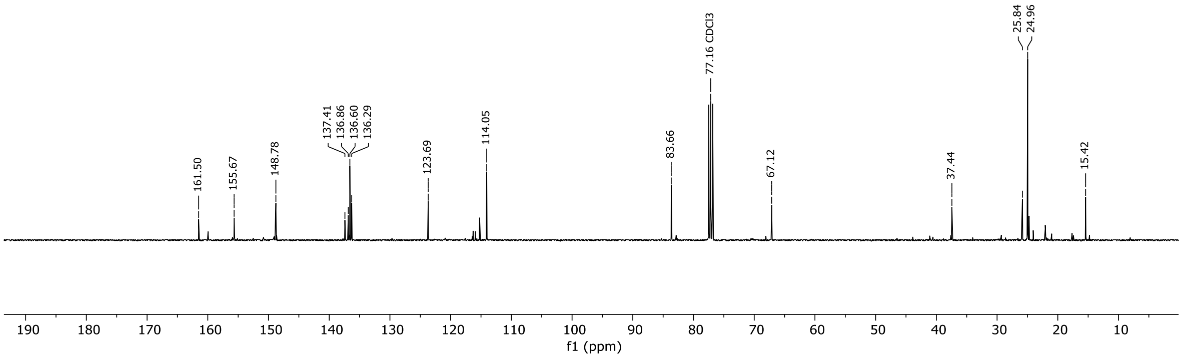

42, ^{1}H NMR (400 MHz, Acetone-d_6)

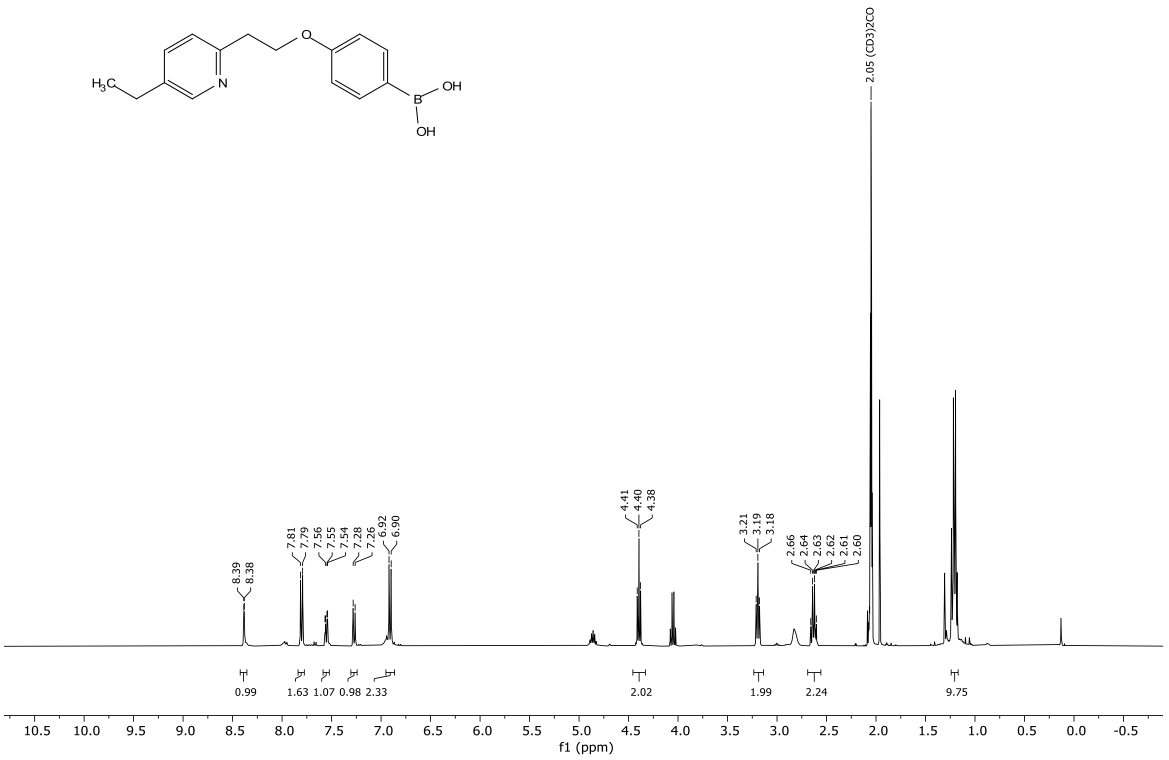

43, ^{1}H NMR (400 MHz, $CDCl_3$)

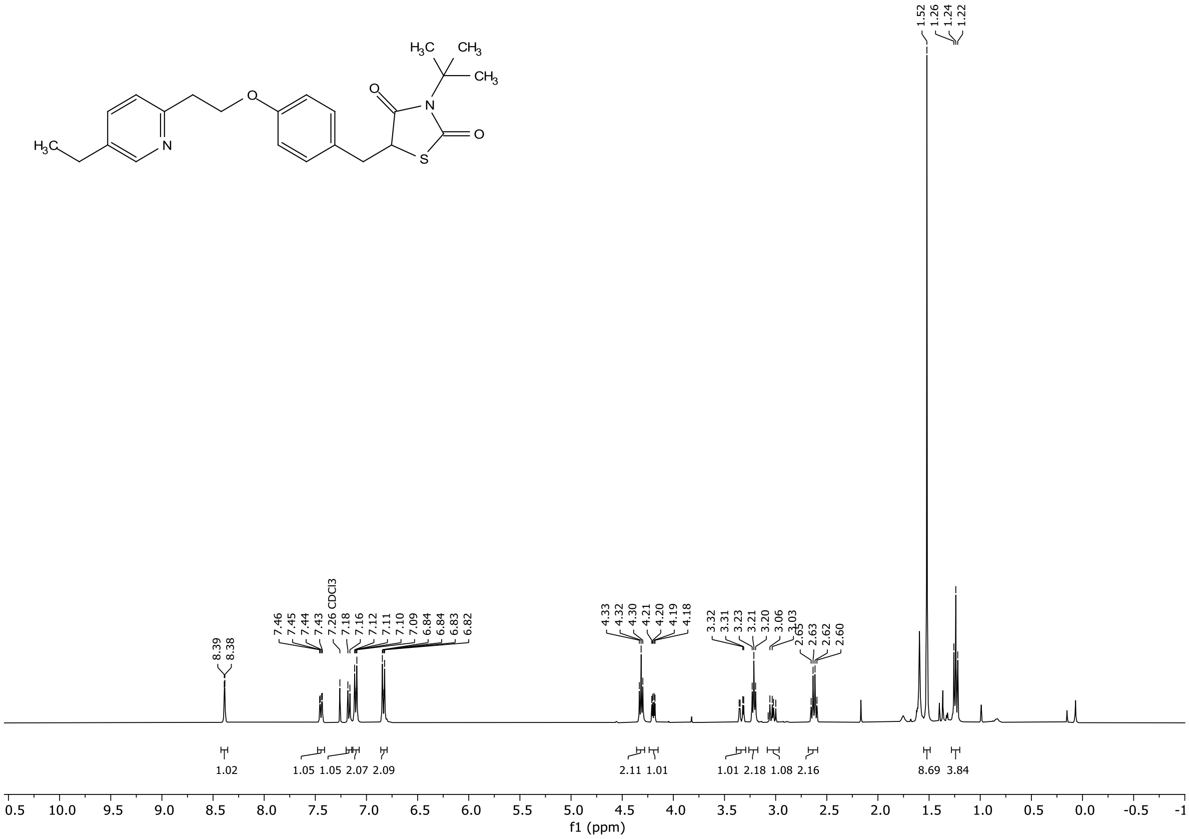

43, **^{13}C{1H} NMR** (101 MHz, $CDCl_3$)

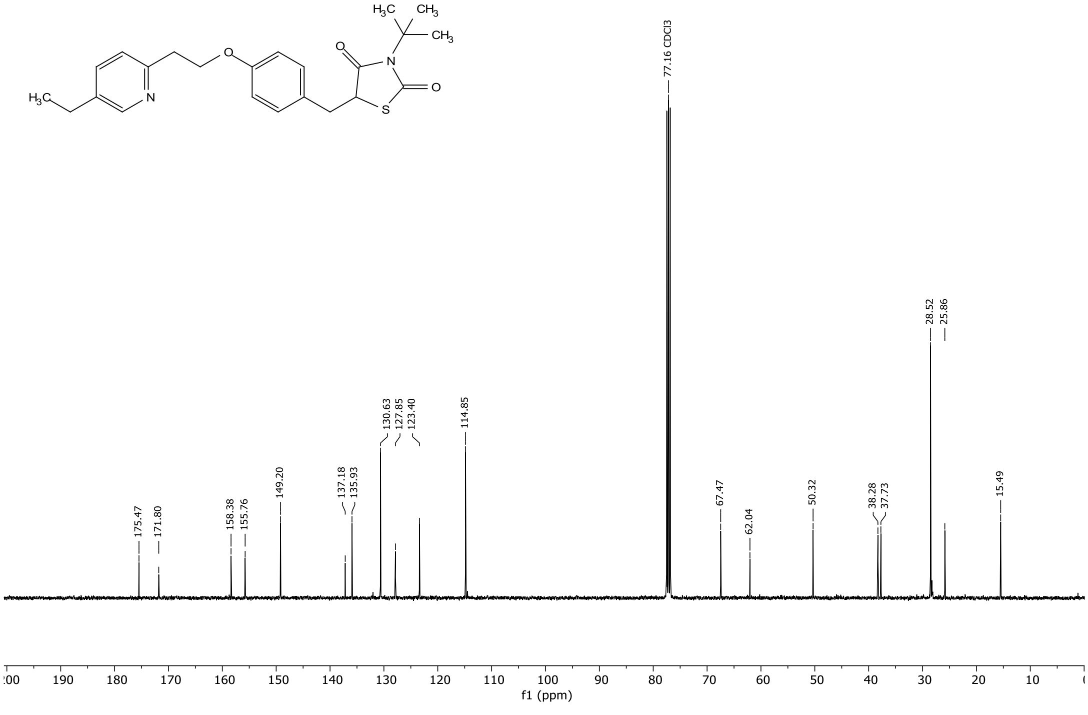

44, ^{1}H NMR (400 MHz, DMSO-d_6)

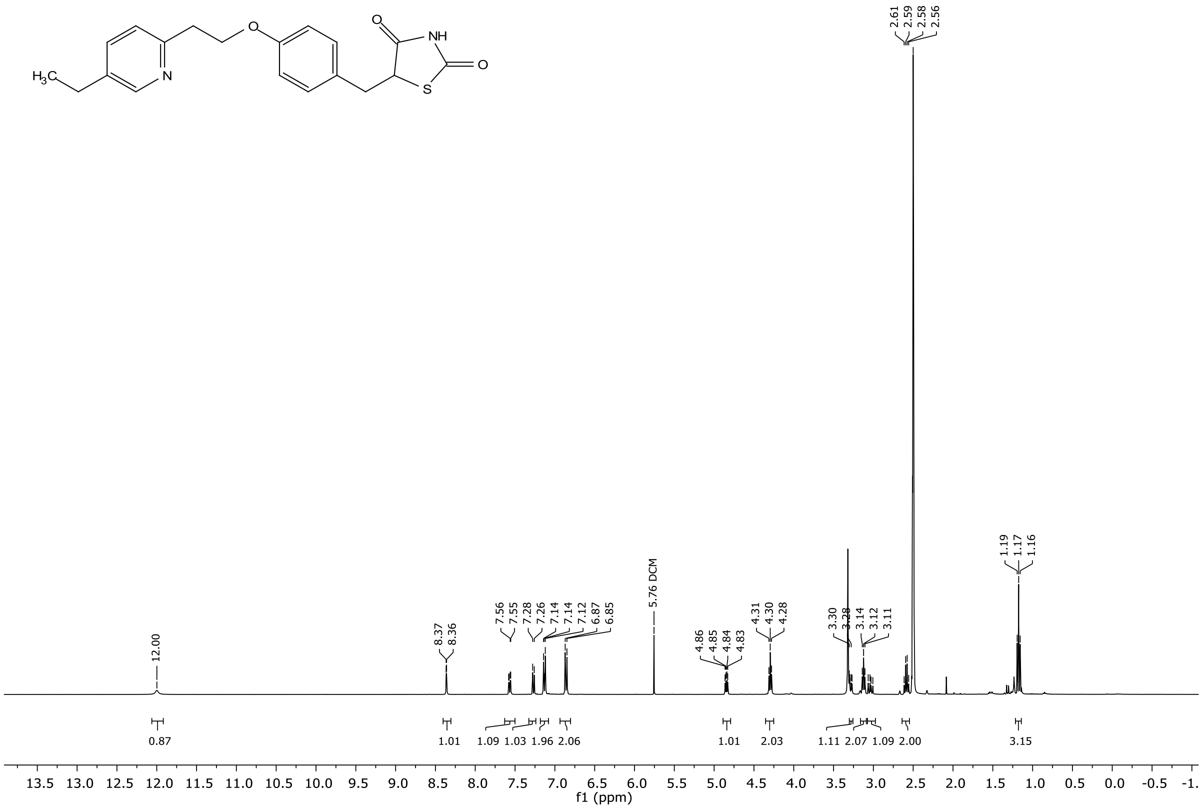

44, ^{13}**C{1H} NMR** (101 MHz, DMSO-d_6)

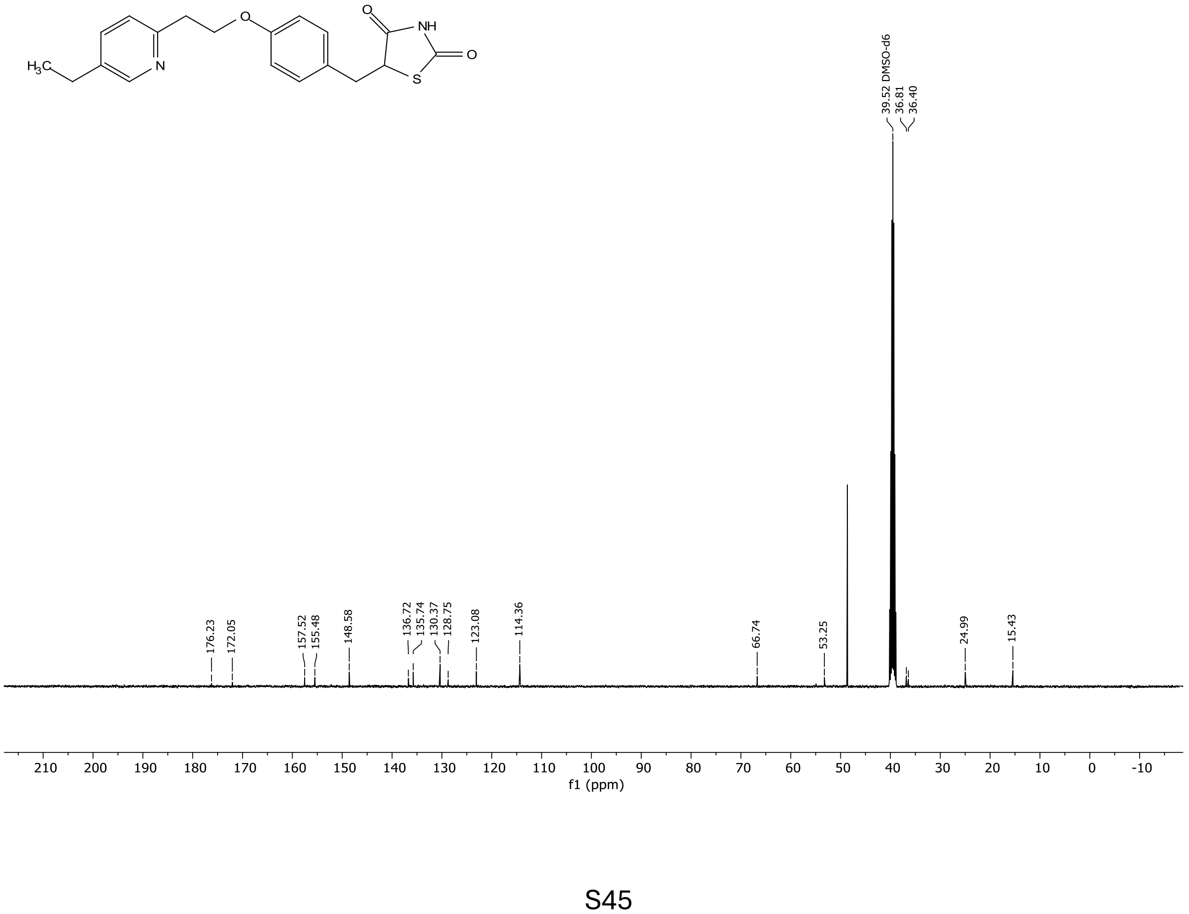

45, ^{1}H NMR (101 MHz, $CDCl_3$)

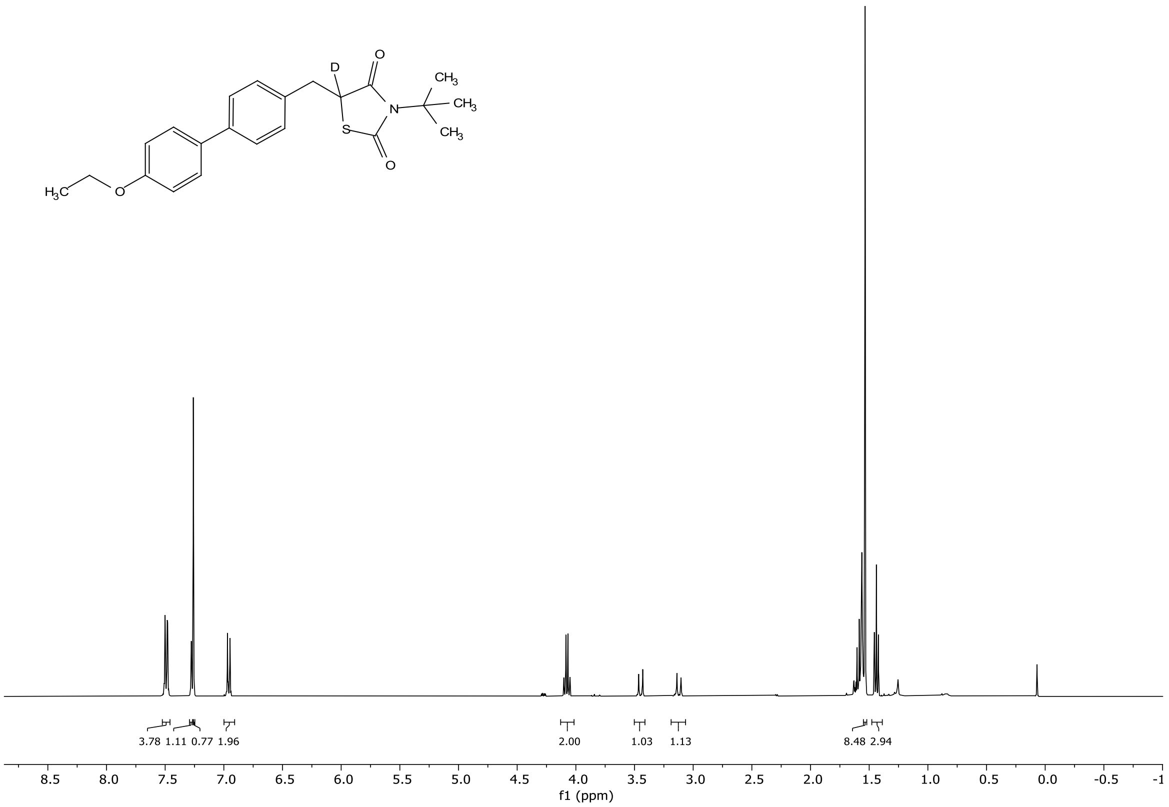

45, **^{13}C{1H} NMR** (101 MHz, $CDCl_3$)

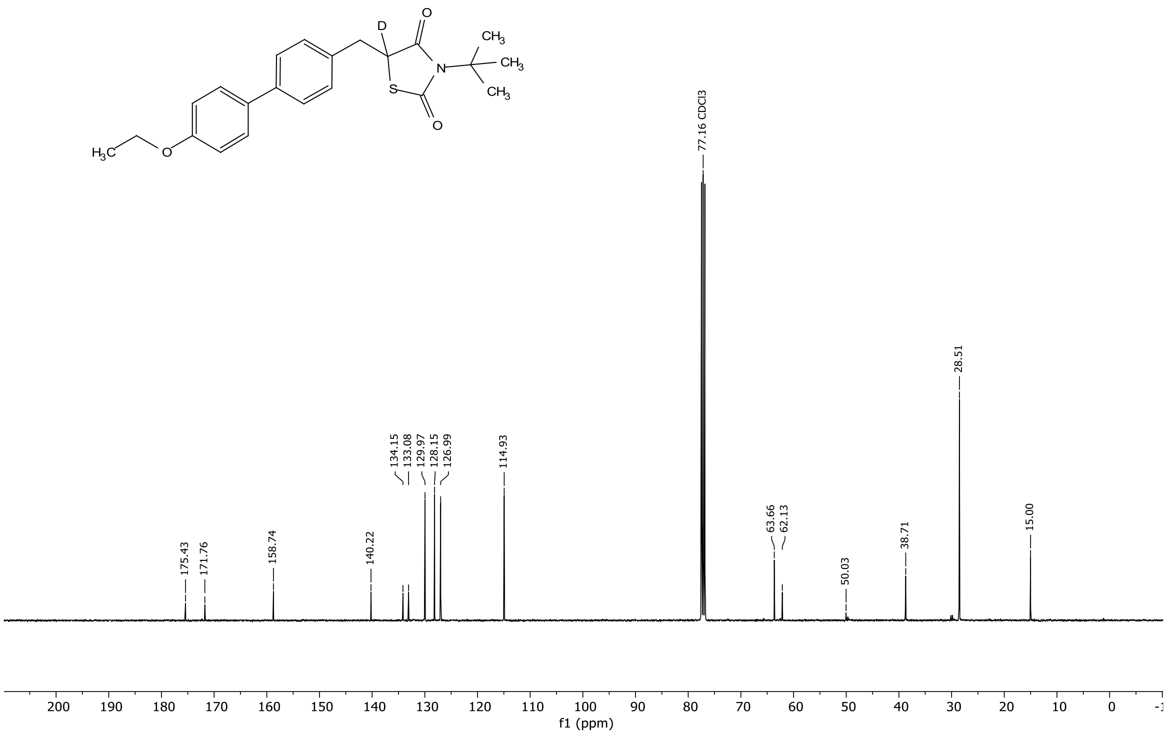

Supplement: Supplementary file 1 [file jo5c01306_si_001.pdf]
